# Evolutionary basis of male same-sex sexual behavior by multiple pheromone switches in *Drosophila*

**DOI:** 10.1016/j.cub.2026.02.046

**Published:** 2026-03-20

**Authors:** Youcef Ouadah, Thomas H. Naragon, Hayley Smihula, Emily L. Behrman, Mohammed A. Khallaf, Yun Ding, David L. Stern, Joseph Parker, David J. Anderson

**Affiliations:** 1 Division of Biology and Biological Engineering, Tianqiao and Chrissy Chen Institute for Neuroscience, and Howard Hughes Medical Institute, Caltech, Pasadena, CA 91125, USA; 2 Division of Biology and Biological Engineering, Caltech, Pasadena, CA 91125, USA; 3 Janelia Research Campus, Howard Hughes Medical Institute, Ashburn, VA 20147, USA; 4 Department of Evolutionary Neuroethology, Max Planck Institute for Chemical Ecology, Jena 07745, Germany; 5 Department of Biology, University of Pennsylvania, Philadelphia, PA 19104, USA; 6 Present address: Department of Biochemistry and Biophysics, UCSF, San Francisco, CA 94158, USA; 7 Present address: Department of Biological Sciences, Dartmouth College, Hanover, NH 03755, USA; 8 Present address: Molecular Physiology of Somatic Sensation Laboratory, Max Delbrück Center for Molecular Medicine, Berlin 13125, Germany; 9 Present address: Department of Zoology and Entomology, Faculty of Science, Assiut University, Assiut 71515, Egypt; 10 Present address: Stowers Institute for Medical Research and Howard Hughes Medical Institute, Kansas City, MO 64110, USA; 11 Lead contact

## Abstract

Male same-sex sexual behavior (SSB) is widespread among animal species, but its proximate (mechanistic) and ultimate (evolutionary) explanations remain unclear. A prevailing view is that SSB reflects impaired sex recognition, especially in insects. By unbiased behavioral screening, we identified a *Drosophila* species, *D. santomea*, in which males seldom attack and spontaneously court males vigorously, in addition to females. Behavioral, chemical, and optogenetic neuronal manipulations indicate that *D. santomea* males can distinguish conspecific sex and retain functional aggression circuitry. Instead, male SSB reflects three evolved pheromonal changes affecting two separate signaling systems, resulting in both reduced pheromone production and behavioral valence reversal. One of these occurs unexpectedly in females and may have evolved to prevent hybridization with an interfertile, geographically overlapping sibling species. Remarkably, male SSB and similar pheromonal changes also selectively co-occur in *D. persimilis*, a geographically and phylogenetically distant species and member of another sympatric sibling pair, implying evolutionary convergence in the two young taxa. The results identify a pheromonal mechanism for rapid social evolution in *Drosophila* and suggest a plausible evolutionary origin for male SSB as arising in concert with female adaptations that ensure reproductive isolation during speciation.

## In brief

Ouadah et al. discover a *Drosophila* species in which males spontaneously court rather than attack other males. Their social evolution is traced to three changes in pheromone signaling, which connect the emergence of male same-sex sexual behavior to the need in females to maintain reproductive isolation from an interfertile sibling species.

## Graphical Abstract

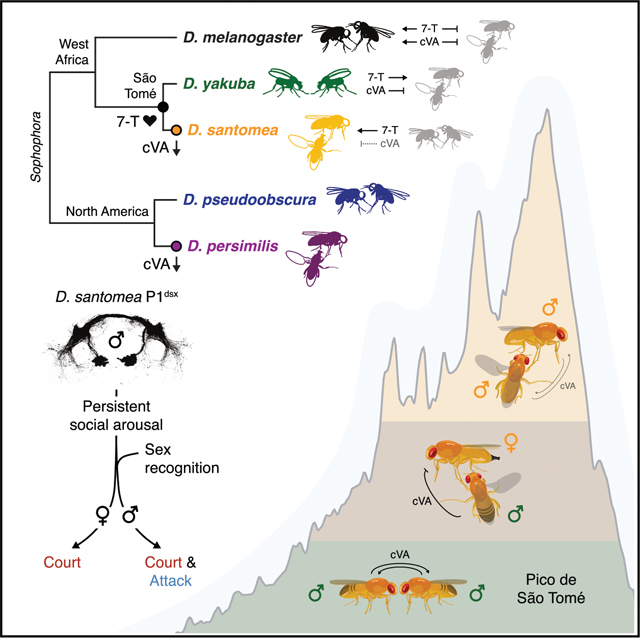

## INTRODUCTION

Social behaviors, including aggression, courtship, mating, parenting, and cooperation, are remarkably diverse in the animal kingdom^1^ and can vary greatly between entire orders^2^ or even closely related sibling species.^3^ The fitness benefits of social behaviors have received considerable theoretical attention,^4–6^ and underlying genetic, hormonal, neural circuit, pheromonal, and sensory mechanisms have been identified in several model organisms.^7–9^ Among insects, species of the genus *Drosophila*^10^ have provided powerful model systems for studying social behaviors,^11–16^ as exemplified by a comprehensive understanding of the biology of male courtship and mating in *D. melanogaster*.^17–19^ The study of interspecies variation between *Drosophila* species has identified flexible genetic^20–23^ and neural^24–27^ substrates of rapid courtship evolution and revealed general principles of reproductive function and isolation.^28^ However, this fruitful approach has rarely been applied to other social behaviors.

Aggression is a highly adaptive and virtually universal intrasexual social behavior among males and females of sexually reproducing metazoan species.^29^ It serves to establish and maintain dominance in social hierarchies; protect resources, such as mates, food, or nesting sites, from conspecific competitors^30^; and in humans, promote individual professional or social success as well as defend against territorial incursion.^31^ Interestingly, aggression is tightly linked to courtship and mating at the behavioral as well as neural circuit levels^32^ (e.g., in vertebrates, males often become more aggressive during periods when females are sexually receptive^33^). Perhaps for this reason, relative levels of aggressive and sexual behaviors can vary widely between species and have yielded differing adaptive strategies, as in the well-known case of chimpanzees and bonobos.^34^ However, virtually nothing is known about the underlying molecular, cellular, and circuit-level adaptations that underlie such interspecific behavioral differences.

To address this problem, we sought to identify phylogenetic patterns of variation in intermale aggression and courtship in *Drosophila* and, by leveraging experimental strengths of the genus, reveal their proximate and ultimate evolutionary causes.^35^ Through an unbiased screen, we discovered that *D. santomea* males, a West African island endemic,^36^ show very little aggression and instead vigorous sexual behavior toward other males, including multiple species-typical motor and acoustic features of female-directed courtship. By contrast, male-male courtship in *D. melanogaster* and other close relatives is infrequent among wild-type adults (although it can be increased by various genetic mutations^37^). *D. santomea* males have not lost the intrinsic capacities for sex recognition or intense aggression. Rather, three evolved pheromonal changes from *D. melanogaster* affecting the two cuticular hydrocarbon (CHC) pheromones, (Z)-7-tricosene (7-T) and (Z)-11-*cis*-vaccenyl acetate (cVA), have selectively biased *D. santomea* intermale social behavior heavily toward courtship over aggression. The changes modify both the level of pheromone production and the valence of behavioral responses, affect both females and males, and have occurred before and after divergence from the sympatric sibling species *D. yakuba*. We also identified a second, distantly related species, *D. persimilis*, which shows spontaneous male-male courtship and similar pheromonal changes. *D. santomea* and *D. persimilis* each naturally reside in sympatry with an interfertile sibling species,^38,39^ and in both taxa, females must detect and discriminate against courtship advances by heterospecific suitors to avoid the fitness cost of producing sterile hybrid male progeny.^40,41^ These results define a convergent pheromonal mechanism that serves as the proximate cause for male same-sex sexual behavior^42^ (SSB) in *Drosophila* and suggest that the trait may have ultimately evolved together with adaptations in female behavior that promote reproductive isolation between interfertile species in geographic zones of sympatry.

## RESULTS

### Evolutionary variation in male-male social behavior within the genus *Drosophila*

**Figure 1.**
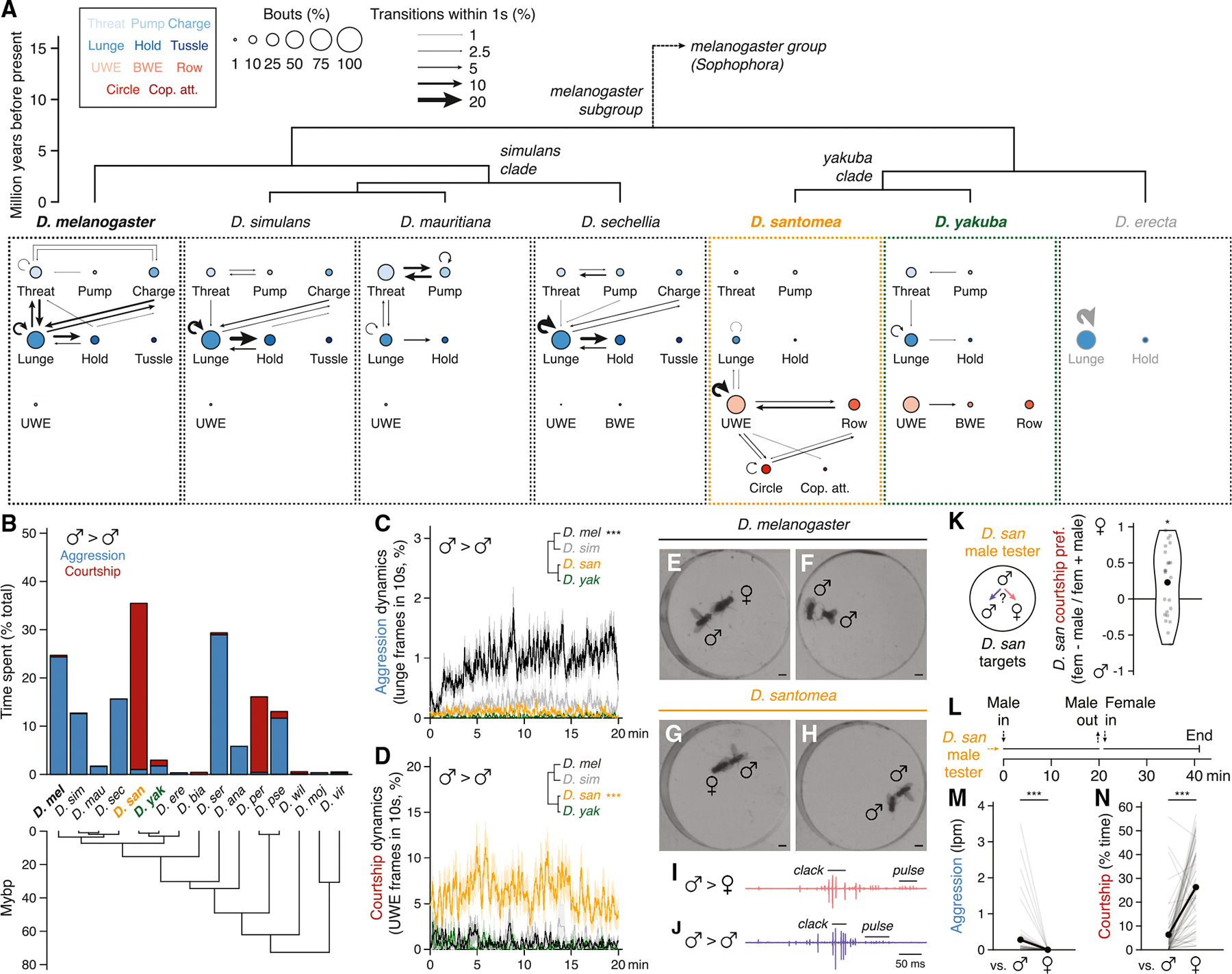
Evolutionary variation in male-male social behavior within the genus *Drosophila* (A) Ethograms from 20 min male-male interactions over food depicting aggressive and courtship actions scored in three representative conspecific pairs per species across the *melanogaster* subgroup. Nodes are bout counts of a given action type, with sizes representing frequency normalized to summed bouts scored across all actions (expert annotation). Edges indicate action transitions, with weights representing the fraction of bouts of a given action (arrow origin) for which a second action (arrow destination) occurred within 1 s. Eleven total actions in each ethogram are arranged in four rows: first, three aggressive threat actions (threat, pump, and charge)^58^; second, three aggressive contact-mediated actions (lunge, hold, and tussle)^47^; third, three courtship actions utilizing the wings and typically directed toward females (UWE, BWE, and row)^14^; and fourth, two courtship actions involving locomotory maneuvers (circle, copulation attempt). Nodes indicating aggressive actions are filled with blue shades and courtship actions with reds. Species arrangement and divergence times according to a compilation of available phylogenies.^59–61^ Note the emergence of male-directed courtship and reduction of aggression in *D. santomea* (orange) and, to a lesser extent, its sympatric sibling species *D. yakuba* (green). *D. erecta* ethogram gray coloration indicates that very few social actions of any kind were observed. UWE, unilateral wing extension; BWE, bilateral wing extension; Cop. att, copulation attempt. See Table S2 for wild-type strains used in figures throughout. (B) Stacked bar plots showing cumulative time spent by paired males in any aggressive (blue) or courtship action (red) as a fraction of the 20 min total interaction. Additional *Sophophora* subgenus species for which male-male interactions were scored for the same action set are included. Note the unusually high time dedication to male-directed courtship by *D. santomea*. *D. serrata* aggression scoring is possibly inflated due to very long holds after each lunge. (C and D) Expressivity and temporal dynamics of male-male aggression (C) and courtship (D) in *D. melanogaster* (black), *D. simulans* (gray), *D. santomea* (orange), and *D. yakuba* (green). Bold traces indicate the mean fraction of frames annotated as lunge (for aggression, C) or UWE (for courtship, D) by automated classifiers in a 10 s sliding window. Envelopes represent standard error of the mean (SEM). Note the high aggression in *D. melanogaster* male-male pairs and high courtship in *D. santomea* throughout the 20 min interactions (significance by Kolmogorov-Smirnov tests). See Table S4 for relevant statistics related to figures throughout. Significant *p* values adhere throughout to the convention *p < 0.05, **p < 0.01, and ***p < 0.001. (E–H) Video stills of *D. melanogaster* (E and F) and *D. santomea* males (G and H) interacting with conspecific female (E and G) or male targets (F and H). *D. melanogaster* court females (E, male shown in UWE) and attack males (F, shown mid-lunge). *D. santomea* court both sexes using a similar set of motor patterns (G and H, males shown in UWE for both) and rarely attack. Scale bars, 1 mm. (I and J) Representative *D. santomea* courtship song traces showing pulse and clack emitted toward both females (I, pink) and males (J, purple). (K) Courtship preference index exhibited by *D. santomea* male testers toward conspecific female and male targets presented simultaneously in 10 min trio assays. Index calculated as the difference in time spent courting each sex normalized by the sum. Individual data points are shown over a violin plot with a large dotted mean. Significance compared with a null median of zero (indicating no preference) was determined by one-sample Mann-Whitney U test. *D. santomea* shows a slight but significant preference for courting females over males. (L) Scheme for assessing social behavioral differences by *D. santomea* male tester flies paired consecutively with a male then female conspecific target for 20 min each. (M and N) Aggression (M, measured as lunges per minute) and courtship (N, fraction of total interaction time performing UWE) exhibited by *D. santomea* male testers toward male then female targets in sequential pairings. Data points derived from the same tester are paired, and distribution means are shown as paired black dots. Aggression toward males is never also observed toward females and significantly increased courtship rates are shown toward females (paired Mann-Whitney U tests). See also Figures S1–S3 and Tables S2 and S4.

In the presence of food or a female, pairs of single-housed *D. melanogaster* males will fight,^43–46^ employing a stereotyped set of contact- and non-contact-mediated motor actions including lunging, tussling, and wing threat.^47,48^ We assessed aggression (and other intermale social behaviors) comparatively in 15 *Drosophila* species using food as the stimulating resource,^49^ screening for qualitative and quantitative variation in male social engagement, frequency, and motor pattern. We sampled species densely within the *melanogaster* subgroup (7 species tested of 9 assigned^50^) to monitor potential behavior variation at close phylogenetic distances to *D. melanogaster* with high resolution. At larger evolutionary distances, we sampled sparsely (typically one species per subgenus group or subgroup) to broadly survey genus social evolution, apart from including both members of the *D. persimilis*/*D. pseudoobscura* sibling species pair. We standardized assay conditions but occasionally varied the food source over which flies competed to account for host specializations.^51^ Fly behavior was measured in dyadic male-male interactions by combining saturated manual scoring of 11 social actions on a subset of representative recordings (Figures 1A, 1B, and S1A) with automated behavior classifications^52–54^ for the remainder (Figures S2A and S2D). Separate behavior classifiers detected either lunge, a major consummatory attack action in males,^55–57^ or unilateral wing extension (UWE), a cross-genus indicator of male sexual behavior,^12–14^ as proxies for aggression and courtship, respectively.

Aggression among *D. melanogaster* males was the most prominent and intense of any *melanogaster* subgroup species tested (Figures 1A–1C, S2B, and S2C). Species in the neighboring *simulans* clade (*D. simulans*, *D. mauritiana*, and *D. sechellia*) also fought, though less frequently, using a similar set of actions (Figures 1A–1C, S2B, and S2C). Varying foods usually had little effect on the behavior of specialists, with the notable exception of *D. sechellia*, which fought significantly more over *Morinda* than the standard apple juice (Figure S2G). Male-male interactions in *D. melanogaster* and *simulans* clade species all included very little recognizable courtship, and the few bouts we observed never progressed to attempted copulations (Figures 1A and 1B). Thus, intermale aggressive actions in the subgroup branch that includes *D. melanogaster* are almost entirely distinct from the courtship that males perform toward females (Figures 1E, 1F, S3A, and S3B).

The other branch of the *melanogaster* subgroup, which includes *D. erecta*, *D. yakuba*, and *D. santomea*, exhibited dramatically different male-male dynamics. *D. erecta* males hardly interacted or locomoted, even on a *Pandanus* substrate^62^ (Figures 1A, 1B, S1B, S2G, and S2H). *D. yakuba* and *D. santomea*, by contrast, exhibited low levels of aggression and multiple distinct, clearly recognizable courtship actions (Figures 1A and 1B). Male-male courtship in both sibling species was visually similar to that toward females (Figures 1G, 1H, S3A, and S3B), but the two differed markedly from each other in penetrance and expressivity. Courtship and attack between *D. yakuba* males were both infrequent, whereas *D. santomea* males courted other males considerably more than they fought them and far more than did *D. yakuba* or any other subgroup species (Figures 1B–1D). High levels of male-directed courtship and low aggression were conserved in three additional *D. santomea* strains (Figures S2B, S2C, S2E, and S2F), were not limited to the initial moments of an encounter when sex identification might be incomplete^63^ (Figure 1D), and sometimes culminated in copulation attempts (Figure 1A). Courtship audio recordings^64^ confirmed that *D. santomea* males sang to males as they did to females, emitting both “pulse” and “clack” songs^65–67^ with similar (though not identical) acoustic features (Figures 1I, 1J, and S3C–S3L).

Thus, under our conditions, we find a striking difference in intermale social behavior between *D. melanogaster* and *D. santomea*. *D. melanogaster* and *simulans* clade relatives attack males with varying frequencies using a conserved set of motor actions, whereas *D. santomea* courts both males and females with nearly indistinguishable visual and acoustic patterns. Importantly, *D. santomea* male-directed courtship is not due to sex misidentification. *D. santomea* males exhibited a slight but significant preference for courting females over males in a trio assay (Figure 1K), and individual males that courted and sometimes also attacked other males immediately switched to exclusive and more vigorous courtship when encountering a female (Figures 1L–1N).

### The abundant and monomorphic cuticular pheromone 7-tricosene is sexually attractive to *D. santomea* males

**Figure 2.**
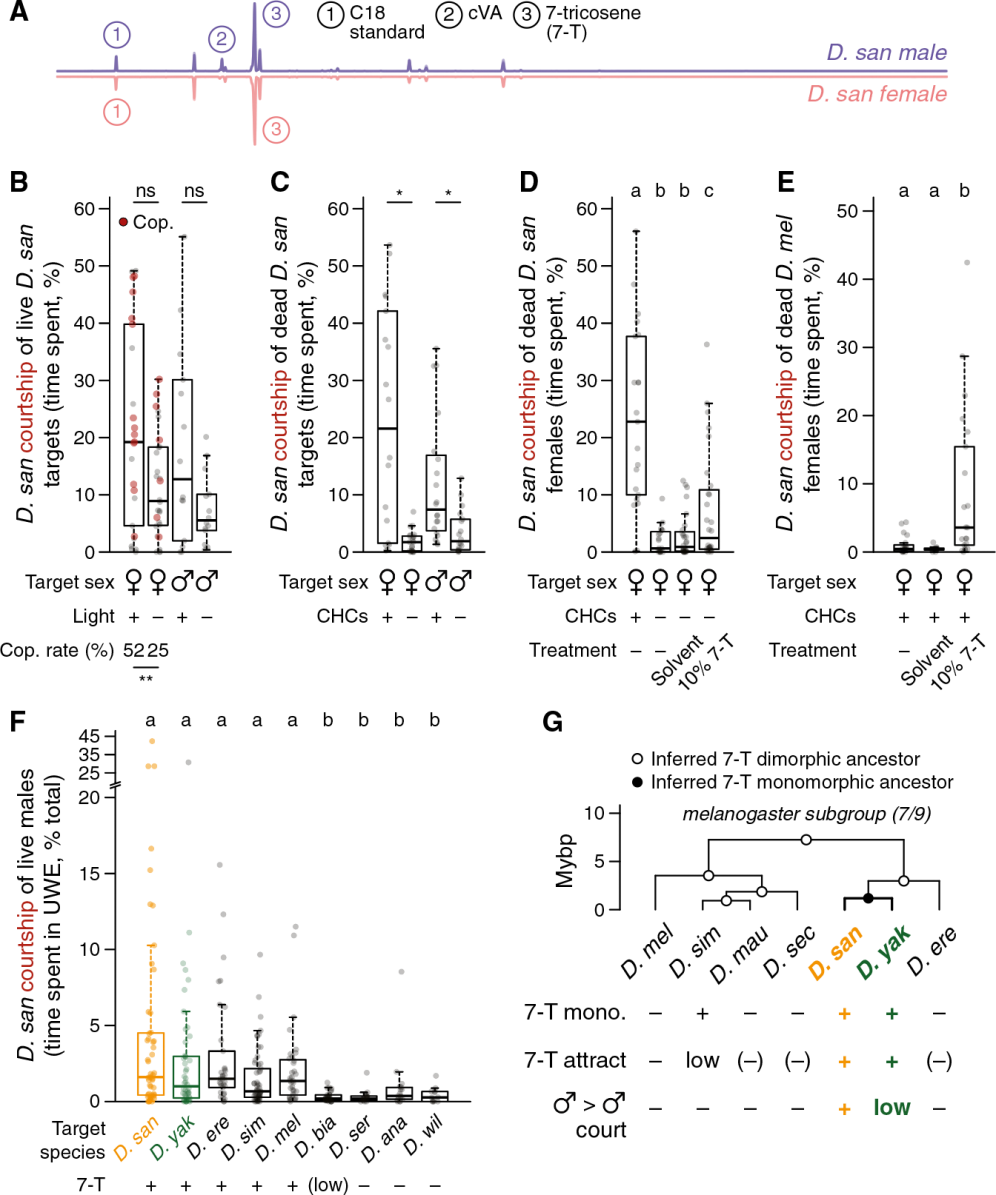
The abundant and monomorphic cuticular pheromone 7-tricosene is sexually attractive to *D. santomea* males (A) Mirrored gas chromatograms representing male (purple, top) and female (pink, bottom) *D. santomea* cuticular hydrocarbon (CHC) profiles. Individual measurements were made on hexane extracts from 10 pooled, age-matched adult flies (3 male, 2 female replicates). Peaks representing the internal standard (C18, 1), male-specific (Z)-11-*cis*-vaccenyl acetate (cVA, 2), and monomorphic (Z)-7-tricosene (7-T, 3) are indicated. (B) Fraction of time *D. santomea* males spend courting conspecific females or males in ambient light vs. dark conditions during 10 min interactions. Boxplots show the full distribution range within whiskers (excluding statistically identified outliers) and second and third quartiles within boxes, with medians in bold. Individual data points (including outliers) overlaid as gray or, for males courting females that successfully copulated, red dots. Outliers are retained in all summary metrics and statistical comparisons here and throughout. Note the slight but non-significant decreases in courtship toward both sexes in the dark (Mann-Whitney U tests), although thecopulation rate with females is significantly reduced (binomial test). (C) Fraction of time *D. santomea* males spend courting conspecific dead females or males with or without endogenous CHCs (removed by hexane wash) during 10 min interactions. Significantly reduced courtship without CHCs for both target sexes (Mann-Whitney U tests). (D) Similar to (C), with a 10% 7-T add-back (20 μg/fly) or hexane solvent control to conspecific dead females after CHC removal. 7-T treatment partially restores attractiveness to *D. santomea* male testers (lettered statistical groupings assigned by post-hoc Dunn’s tests following significant Kruskal-Wallis). Cohen’s *d* effect size for 10% 7-T addition (fourth) vs. no addition after CHC wash (second), 0.692 (95% confidence interval [CI] −0.379 to 0.821, *p* = 0.401). Cohen’s *d* effect size for solvent addition (third) vs. no addition after CHC wash (second), 0.221 (95% CI 0.076–1.31, *p* = 0.042). Cohen’s *d* effect size for 10% 7-T addition (fourth) vs. solvent addition (third), 0.617 (95% CI 0.069–1.17, *p* = 0.039). (E) Fraction of time *D. santomea* males spend courting dead *D. melanogaster* females (low endogenous 7-T) perfumed with 10% 7-T or solvent control (hexane). 7-T addition elicits courtship from *D. santomea* males. Significance by Dunn’s tests. (F) Fraction of time *D. santomea* males spend courting conspecific (orange) or heterospecific males (*D. yakuba*, green; all others black) during 20-min interactions. 7-T presence in each species from GC-MS and phylogeny shown below; statistical groupings by Dunn’s tests above. Note the correlation between 7-T presence and elevated *D. santomea* courtship. (G) Summarized indications of 7-T monomorphism,^68,69^ evidence for 7-T as a sexual attractant,^24,26,76,80^ and binarized male-male courtship expression in 7 of the 9 *melanogaster* subgroup species arranged by phylogeny (*D. teissieri* and *D. orena* omitted). “(−)” indicates dimorphic species for which 7-T is not expected to be sexually attractive but has not yet been formally tested. Pheromone status of common ancestors at phylogeny branch points inferred by parsimony. See also Figure S4 and Tables S1, S2, and S4.

Five of the nine *melanogaster* subgroup species (*D. simulans*, *D. santomea*, *D. yakuba*, *D. teissieri*, and *D. orena*) are considered pheromonally monomorphic because the same CHC is the most abundant compound on the cuticles of females and males.^68–70^ For all five, the defining monomorphism is of 7-T (Figures 2A, S4A, and S4B), a nonvolatile compound detected via contact chemosensation.^71–74^ *D. santomea* courts in the dark^75^ (although copulation rate is reduced) (Figure 2B), and removing CHCs in bulk from either females or males strongly diminishes their attractiveness (Figure 2C), implying the existence of a courtship-promoting pheromone(s) shared between sexes. That 7-T is an aphrodisiac signal for *D. santomea* males is supported by monomolecular add-back following bulk CHC removal from *D. santomea* females (Figure 2D), ectopic addition to intact females of species lacking endogenous 7-T (*D. melanogaster* and others) (Figures 2E and S4C–S4F), and positive correlation between 7-T presence and courtship elicited from *D. santomea* males in a variety of heterospecific male-male pairings (Figure 2F). Thus, 7-T appears necessary and is sufficient to evoke sexual attraction in *D. santomea* males. The inversion from 7-T suppressing courtship in *D. melanogaster* (where it is male specific)^71,76–80^ to promoting courtship in *D. santomea* is shared with *D. yakuba*,^26^ suggesting that the 7-T valence switch arose in tandem with or soon after the switch to pheromonal monomorphism in the *D. santomea*/*D. yakuba* common ancestor on mainland Africa^50^ (Figure 2G).

### Selectively low cVA abundance on *D. santomea* males amplifies intermale courtship

**Figure 3.**
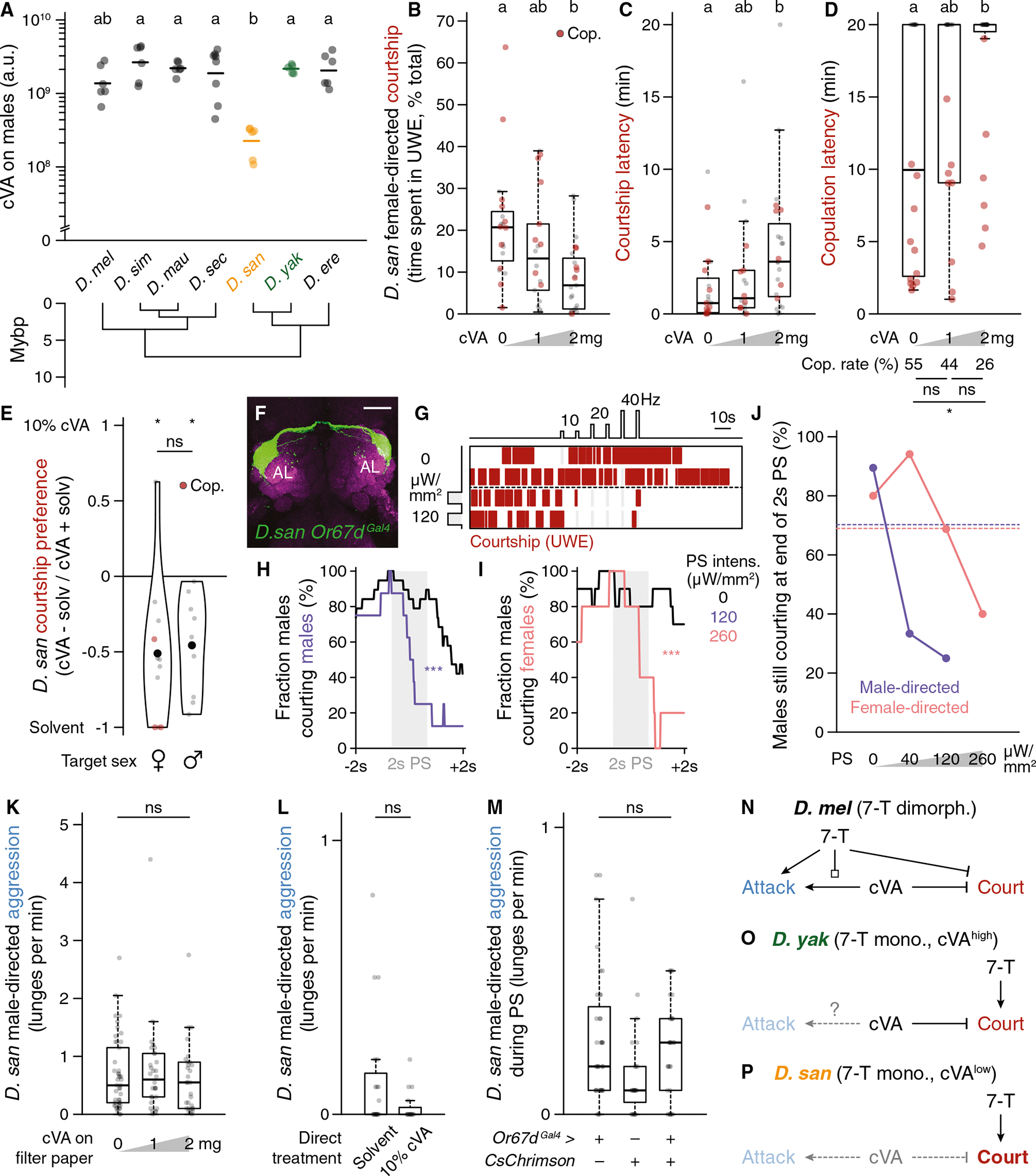
Selectively low cVA abundance on *D. santomea* males amplifies intermale courtship (A) cVA abundance on males of the *melanogaster* subgroup measured in single flies (5–8 individuals per species) by TD-GC-MS. Statistical groupings by Dunn’s tests; a.u., arbitrary units. Note the low cVA level selectively in *D. santomea* males. Data derived from Khallaf et al.^70^ with permission from the authors. (B–D) *D. santomea* sexual behavior during 20 min interactions with females in the presence of increasing cVA on a piece of filter paper nearby (0, 1, or 2 mg). (B) Fraction of time spent courting. (C) Latency to first courtship bout. (D) Latency to copulation. Statistical significance by Dunn’s tests, binomial tests for copulation rates. Note the decreased courtship, increased latency, and decreased copulation rate at the highest cVA abundance (2 mg). (E) Preference index for *D. santomea* courtship toward 10% cVA (20 μg/fly) vs. solvent control (acetone) perfumed live target female or male conspecific flies during 20-min trio interactions. The index is calculated as the difference in time spent courting each target normalized by the sum. Significance between distributions and between each distribution and a null median of zero (indicating no preference) determined by two- and one-sample Mann-Whitney U tests, respectively. *D. santomea* male testers exhibit clear aversion to cVA-performed targets of either sex. All three observed copulations occurred with solvent control females. (F) Photomicrograph showing the labeling pattern of *Or67d*^*Gal4*^ in the antennal lobes (AL) of a *D. santomea* male. Immunostaining for Gal4-dependent cytoplasmic tdTomato pseudocolored green with Bruchpilot synaptic counterstain (using nc82 monoclonal antibody) in magenta. Uniglomerular labeling pattern in each AL matches the expected size and position of cVA-sensitive DA1.^90,100^ Scale bar, 25 μm. See Table S3 for genotypes of transgenic strains used in figures throughout. (G) Courtship rasters (red) for a single-housed *D. santomea* male carrying *Or67d*^*Gal*4^ and Gal4-dependent CsChrimson paired with a group-housed wild-type conspecific male target. Two representative sham trials (above dash) and two photostimulation (PS) trials (below dash) are shown. 2 s PS blocks repeat every 10 s for a total of six activations after a 1 min baseline. PS frequencies increase in pairs from 10 to 40 Hz within each trial and increase in intensity across trials. PS immediately interrupts spontaneous courtship by tester males in experimental but not sham trials. (H and I) Quantification of (G) showing the fraction of *Or67d*^*Gal4*^ tester males courting males (H) or females (I) during 6 s time windows surrounding PS blocks (H, purple; I, pink) or sham controls (black). Data were filtered for blocks where tester males showed spontaneous courtship within 0.5 s of PS onset. Data from all three frequencies were grouped since they gave similar results but were separated by intensity: 120 (H) or 260 μW/mm^2^ (I). Most tester males cease courtship by PS offset in experimental trials (significant for both target sexes by Kolmogorov-Smirnov tests). (J) Summary of courtship suppression effects in *Or67d*^*Gal4*^ tester males as a function of PS intensity with male (purple) and female (pink) targets. Baselines (horizontal dashed lines) were calculated as the fraction of spontaneous courtship bouts in sham trials lasting more than 2 s. Note a strong effect with male targets at low intensities but a comparable effect only at the strongest intensity with females. (K) *D. santomea* male aggression during 20 min interactions between single-housed males in the presence of increasing cVA on filter paper. Not significant by Kruskal-Wallis test. (L) *D. santomea* male aggression toward males directly perfumed with 10% cVA or solvent control (acetone) during 20 min trio interactions. Not significant by Mann-Whitney U test. (M) Aggression in *D. santomea* males carrying *Or67d*^*Gal4*^ and Gal4-dependent CsChrimson or genetic controls (with either tdTomato in place of CsChrimson or an “empty” Gal4 driver^101^ in place of *Or67d*^*Gal4*^) during 12 min photoactivation trials. Pairs of group-housed males are exposed to six 30 s PS blocks with fixed intensity and monotonically increasing frequency (1–40 Hz), with a 2 min baseline and 1 min interblock intervals. Measurements represent cumulative lunge counts from all six PS blocks. Note the insufficiency of *Or67d*^*Gal4*^ activation to induce attack (no significance between genotypes by Kruskal-Wallis test). (N–P) Logic models for control of male social behaviors by 7-T and cVA in *D. melanogaster* (7-T dimorphic, N), *D. yakuba* (7-T monomorphic, O), and *D. santomea* (7-T monomorphic with reduced cVA, P). In *D. melanogaster*, where 7-T inhibits intermale courtship, 7-T also gates the effect of cVA to promote aggression via Gr32a.^76^ A switch in 7-T function from suppressing to stimulating courtship in monomorphic species is predicted to reduce or eliminate the aggression-promoting effect of cVA without impacting its courtship-suppressing effect, as evident in *D. santomea*. Intermale courtship promoted by 7-T in *D. santomea* is further amplified due to reduced inhibition by cVA. Edge terminating in a white box (N) indicates a permissive “gating” effect by 7-T on cVA-induced aggression, in addition to its direct aggression-promoting effect. See also Figure S5 and Tables S2, S3, and S4.

If *D. santomea* sexual attraction uses an abundant and monomorphic signal (7-T), then it would seem unsurprising that male-directed courtship is a corollary outcome of this relatively simple pheromone arrangement. However, intermale courtship in *D. yakuba* and other 7-T monomorphic species is rare^81^ (Figure 2G), hinting that *D. santomea* males have either gained an additional courtship-promoting signal absent in the other species or lost a courtship-inhibiting signal otherwise present. Consistent with the latter, we found that *D. santomea* males produce significantly less cVA than other subgroup members (84%–92% reduction, 89% less than *D. yakuba*) (Figures 3A and S5A–S5G). cVA is a semi-volatile olfactory pheromone produced in the ejaculatory bulb of male drosophilids^82^ and has pleiotropic social roles, including promoting aggregation^83–85^ and aggression^86–89^ and suppressing courtship,^80,90–92^ either natively on males or after transfer to females during copulation.^93–95^ Behavioral dose-response curves with cVA applied onto a piece of filter paper indicated that cVA indeed suppresses courtship in *D. santomea* (Figures 3B–3D), with the primary effect being to increase latency to initiation (Figure 3C). In trios, *D. santomea* males showed nearly universal preference for courting solvent control-treated males or females over flies directly coated with cVA (Figure 3E). Triggering fictive cVA sensation in *D. santomea* males by phasic optogenetic activation of Or67d-expressing olfactory sensory neurons (OSNs)^90,96–98^ was also sufficient to interrupt natural courtship toward conspecific partners of both sexes (Figures 3F–3I and S5H–S5J), as is observed in *D. melanogaster* males paired with females.^99^ Interestingly, the photostimulation light intensity needed to pause ongoing courtship by *D. santomea* was lower with male targets than with females (Figure 3J), consistent with some courtship suppression even by the low endogenous amount of cVA present on males.

Unlike its courtship-inhibiting function, cVA’s aggression-promoting function in *D. melanogaster* does not appear to be conserved in *D. santomea* males. cVA addition experiments, either onto filter paper or directly onto live target males, showed no effect of inducing aggression (Figures 3K and 3L), nor did strong and direct photoactivation of Or67d OSNs (Figure 3M). Pairings with unmanipulated heterospecific male partners presenting high endogenous cVA levels also failed to significantly increase *D. santomea* attack behavior (Figures S5K–S5M). The ineffectiveness of cVA to promote aggression in *D. santomea* males may be due to the 7-T valence switch described above^76^ (Figures 3N–3P) or could reflect a reduced behavioral sensitivity to cVA relative to *D. melanogaster*.

Sexual attraction promoted by monomorphic 7-T and low levels of the male-specific anti-aphrodisiac cVA together provide a pheromonal basis for selectively enhanced courtship among *D. santomea* males. One curious additional effect was observed, presumably also due at least in part to the low cVA abundance in *D. santomea* males. When paired with males of other *melanogaster* subgroup species that find cVA sexually aversive^26,70,90^ and show minimal sexual behavior in conspecific male-male pairs (*D. melanogaster*, *D. simulans*, and *D. yakuba*) (Figures 1A, 1B, and 1D), *D. santomea* males elicited 200%–580% higher levels of courtship and 53%–91% reduced levels of aggression from the heterospecific partners relative to the amount those species showed in conspecific settings (Figures S5N–S5R). Thus, *D. santomea* male attractiveness extends even across species boundaries to multiple other drosophilids.

### Central aggression circuits in *D. santomea* can evoke intense attack when photoactivated

*D. santomea* intermale sexual behavior is frequent but does not fully saturate the time that two males interact (Figure 1B). Even so, very little attack occurs outside of courtship periods. This observation, in addition to the species-specific ineffectiveness of cVA to promote aggression, prompts the question of whether *D. santomea* aggression circuitry is conserved and functional.

**Figure 4.**
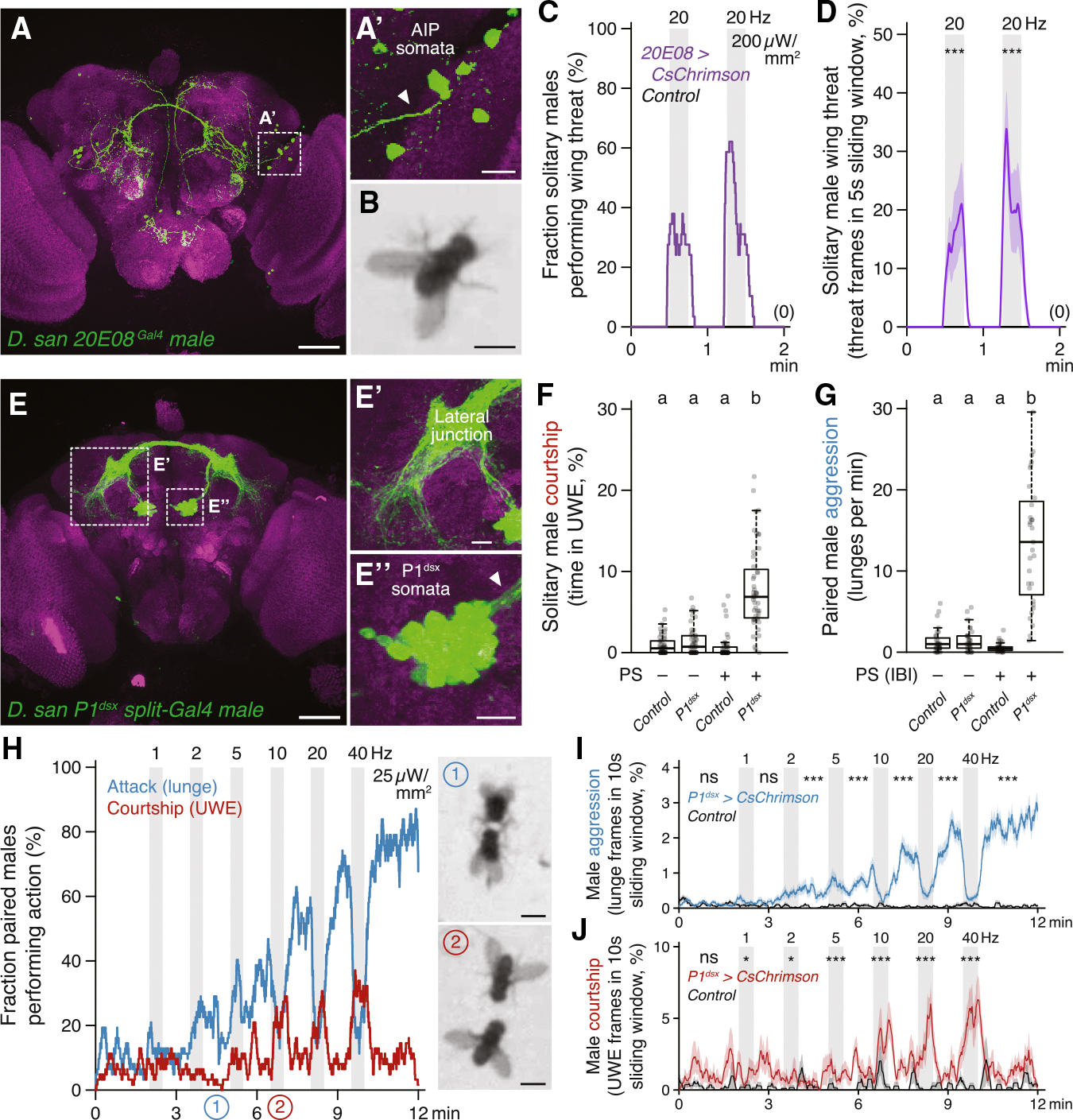
Central aggression circuits in *D. santomea* can evoke intense attack when photoactivated (A) Photomicrograph showing the labeling pattern of *20E08*^*Gal4*^ in the central brain of a *D. santomea* male. Immunostaining for Gal4-dependent cytoplasmic tdTomato (green) with synaptic counterstain (nc82, magenta). Close-up (A′) shows AIP somata and axons (arrowhead) from the boxed region. Scale bars, 50 μm (A) and 10 μm (A′). (B) Video still of bilateral wing elevation (threat) elicited by PS in a solitary *D. santomea* male carrying *20E08*^*Gal4*^ and Gal4-dependent CsChrimson. Scale bar, 1 mm. (C and D) Quantification of PS effects in solitary *20E08*^*Gal4*^ tester males (purple) and genetic controls (homozygous parental lines) exposed to two 15 s PS blocks with 30 s baseline and interblock interval (IBI). (C) Fraction of flies showing threat (penetrance). (D) Mean fraction of frames with flies showing threat in a 5 s sliding window with SEM envelopes (expressivity). Significant induction of threat during PS in *20E08*^*Gal4*^ by Mann-Whitney U tests. (E) Photomicrograph showing *P1*^*dsx*^ split-Gal4 (*71G01*^*DBD*^*;dsx*^*AD*^) labeling in the central brain of a *D. santomea* male. Immunostaining for Gal4-dependent cytoplasmic tdTomato (green) with synaptic counterstain (nc82, magenta). Close-ups show a characteristic region of *P1*^*dsx*^ neuropil (lateral junction, E′) and somata and axons (E″) from the boxed regions. Scale bars, 50 μm (E) and 10 μm (E′ and E″). (F and G) Courtship elicited in solitary (F) and aggression in paired (G) *D. santomea* males carrying *P1*^*dsx*^ split-Gal4 and Gal4-dependent CsChrimson or a genetic control (males missing only the *dsx*^*AD*^ hemidriver) during 12 min photoactivation trials. In both cases, group-housed males are exposed to six 30 s PS blocks with fixed intensity and monotonically increasing frequency (1–40 Hz), with a 2 min baseline and 1 min IBIs (as in H). PS− represents the 2 min baseline period prior to the first PS. PS+ cumulatively represents all six PS blocks (for courtship, G) or all five IBIs and the period following final PS (for aggression, F). See behavior dynamics in (H) for justification of aggression quantification during IBIs. Note the significant PS induction for both actions (Dunn’s tests). (H) Fraction of flies showing attack (lunge, blue) and courtship (UWE, red) in same-genotype pairs of *P1*^*dsx*^ males during six PS blocks with fixed intensity and increasing frequencies. Attacks mostly during light-off periods are punctuated by time-locked courtship during high-frequency PS blocks. Video stills at right show aggressive tussling following 2 Hz PS (1) and simultaneous courtship by both tester males during 10 Hz PS (2). Scale bars, 1 mm. (I and J) Expressivity and temporal dynamics of attack (I) and courtship (J) elicited by PS in *P1*^*dsx*^ male pairs (I, blue; J, red) or the genetic control (black). Means shown with SEM envelopes. Significant attack induction during IBIs and after final PS (I) or courtship during PS compared with control (J) by Mann-Whitney U tests. See also Figure S6 and Tables S2, S3, and S4.

Multiple aggression-promoting neurons have been identified in *D. melanogaster* males,^102,103^ among which are P1/pC1 (and subsets therein)^54,99,104–109^ and AIP (also called CL062).^58,110,111^ We generated transgenic reagents necessary to access P1/pC1 (referred to here as P1^dsx^ neurons to reflect the *71G01*^*DBD*^*;dsx*^*AD*^ split driver combination used) and AIP in *D. santomea* by combination CRISPR-Cas9 knockin^112,113^ and phiC31 recombination into existing landing site lines^114,115^ and tested photoactivation effects in single or paired flies using CsChrimson as the opsin effector.^116^ In comparison to *D. melanogaster*,^58^ AIP neurons had the expected position and morphology in *D. santomea* males (Figure 4A), and photoactivation in solitary flies elicited bilateral wing elevation (i.e., threat display), the major non-contact-mediated form of aggression in *Drosophila*,^44,47,48^ time-locked to the onset and offset of photostimulation (Figures 4B–4D, S6A, and S6B). P1^dsx^ neurons were similarly present with a morphology resembling that in *D. melanogaster*^117,118^ and *D. yakuba*^26^ (Figure 4E), and photostimulation evoked reliable time-locked courtship measured by UWE in solitary *D. santomea* males (Figures 4F and S6C–S6F).

In pairs of *D. santomea* males both experiencing titrated photoactivation, P1^dsx^ stimulations induced a mix of aggressive and courtship actions similar to the effects seen in *D. melanogaster*^54,105^ (Figures 4G–4J and S6G–S6I). Lunge attacks were detected during low-frequency stimulations (significantly at 2 Hz) and escalated in intensity as the stimulus frequency increased (Figures 4H and 4I). Fighting remained persistently elevated following the final photostimulation period for the duration of the recording. 30-s high-frequency stimulations (>10 Hz) at 1-min intervals interrupted the otherwise monotonic increase in attack intensity with temporary phases of courtship and locomotor arrest time-locked to photostimulation (Figures 4H, 4J, S6J, and S6K). Mixed-genotype pairs (in which one of the two photoactivated males was replaced with a wild-type male prepared to be passive during the assay by group housing) confirmed that stimulated aggression was fly-autonomous and not exclusively caused by social feedback (Figure S6L). However, these experiments also revealed some contextual flexibility in the expression of optogenetically evoked attack. P1^dsx^ photoactivated flies in mixed pairs required slightly higher stimulation frequency to initiate attack (5 instead of 2 Hz; Figures 4I and S6L), and their attack intensity remained ~50%–60% lower than in same-genotype pairs throughout the trials (Figure S6M). Interestingly, P1^dsx^ photoactivated males in mixed pairs showed a selective increase in attack but not courtship latency (Figures S6N and S6O), resulting in a shift from nearly equal balance between flies exhibiting attack or courtship first in same-genotype pairs to a majority of cases where courtship preceded attack in mixed pairs (Figure S6P). This suggests that passive male opponents can, to a limited degree, favor the expression of courtship instead of attack by *D. santomea*.

Major nodes within at least two distinct aggression-promoting circuits (P1^dsx^ and AIP) are therefore conserved in *D. santomea*, and both contact- and non-contact-mediated agonistic behaviors can be robustly evoked by their photoactivation. This rules out that the paucity of spontaneous attacks in *D. santomea* male pairs under naturalistic conditions derives from an evolved loss of function of (at least these) aggression circuits. P1^dsx^ experiments reveal that *D. santomea* aggression can be elevated to supernormal intensity and exhibit persistence under optimal photoactivation conditions (~23-fold higher lunge rate observed after 40 Hz P1^dsx^ photostimulation compared with the average during spontaneous interactions; Figures 4I vs. 1C), but the direct effect of courtship dominates and strongly suppresses attack while P1^dsx^ photostimulation is ongoing, as in *D. melanogaster*.^32^ An opponent whose pheromone bouquet activates *D. santomea* P1^dsx^ neurons, especially a non-aggressive one, would therefore be expected to elicit courtship instead of attack as long as the activation perdures.

### A switch from cVA sexual attraction to aversion in *D. santomea* females

**Figure 5.**
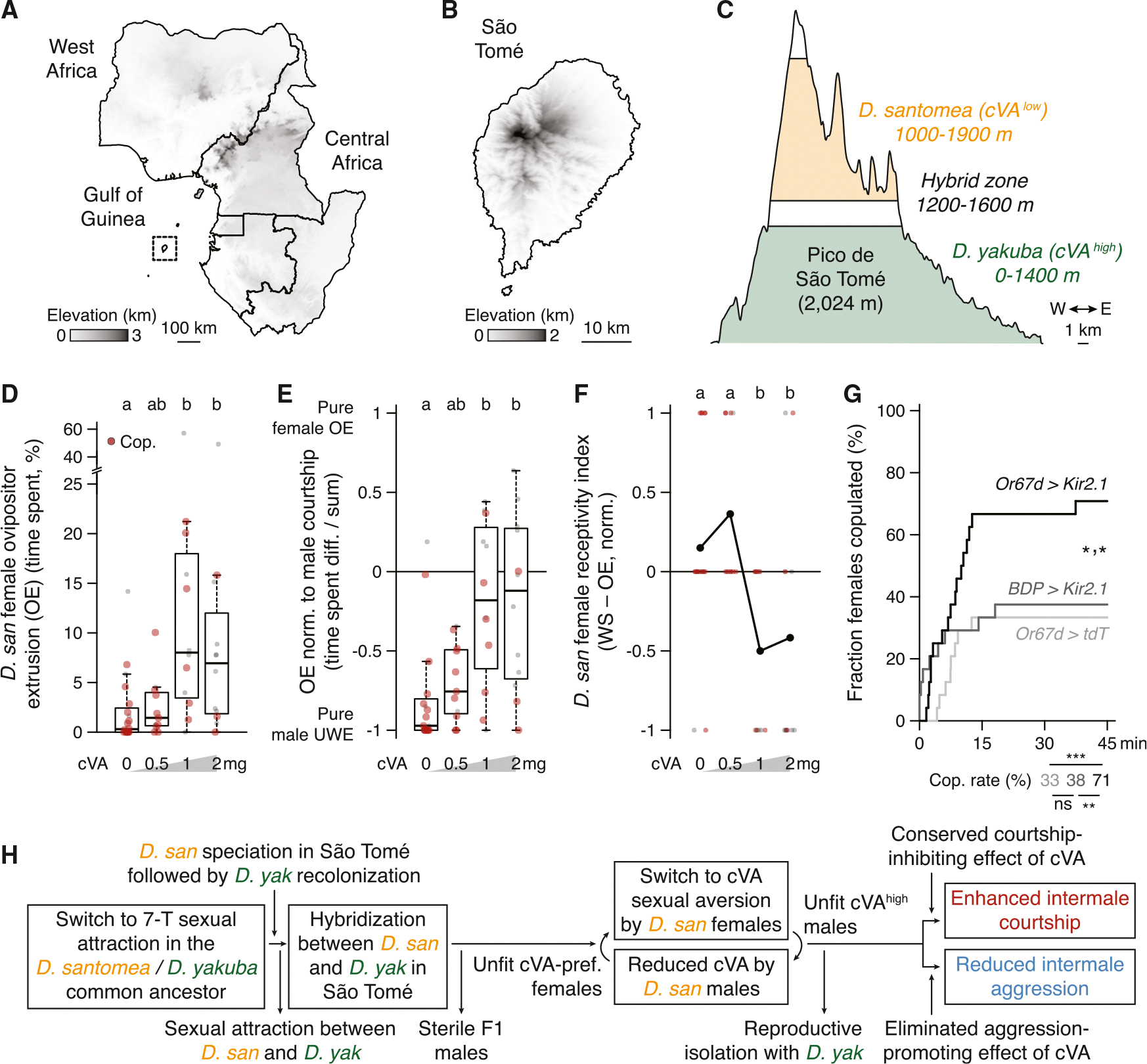
A switch from cVA sexual attraction to aversion in *D. santomea* females (A) Elevation map of equatorial Central and West Africa with the volcanic island of São Tomé in the Gulf of Guinea boxed. Data from Runfola et al.^123^ and Terrain Tiles (Mapzen, accessed Aug. 24, 2025). (B) Elevation map of São Tomé. Note the central summit of Pico de São Tomé. (C) Latitudinal elevation profile of Pico de São Tomé showing *D. santomea* (orange) and *D. yakuba* (green) ranges separated by a hybrid zone where both pure species and F1 hybrid males are found.^119^ (D and E) Behavioral dose-response curves of *D. santomea* female ovipositor extrusion (OE) to increasing cVA on filter paper during conspecific male courtship. (D) Female OE as a fraction of total observation time. (E) Female OE normalized to male courtship (difference divided by the sum of time spent by female in OE and time spent by male in UWE). Red dots, females that copulated. The lowest cVA dose induces an intermediate effect, while both higher doses induce significant increases in OE expression (Dunn’s tests). (F) *D. santomea* female “receptivity index” as a function of increasing cVA. For each female, an index value of 1, 0, or −1 is possible. Observations of female wing spreading (WS, an acceptance behavior) and courtship-normalized OE (rejection, from E) were each binarized as present or absent (WS > 0, OE(norm) > −0.25), and the overall index per dose is calculated as the difference between the two binary values (WS-OE) normalized by the number of females scored. Note significantly decreased sexual acceptance again at the two higher cVA doses (Dunn’s tests). (G) Cumulative copulation latencies and rates by *D. santomea* tester females carrying *Or67d*^*Gal4*^ and Gal4-dependent Kir2.1 (black) or genetic controls (gray) grouped with an equal number of wild-type conspecific males for 45 min in large chambers. BDP, empty Gal4 driver^101^; tdT, tdTomato. 24 total females of each genotype are tested (two trials each of 12 single-housed males × 12 group-housed females). Significance by Kolmogorov-Smirnov tests comparing latencies or binomial tests comparing rates. (H) Evolutionary model for the emergence of high intermale courtship and low intermale aggression in *D. santomea*, incorporating three key pheromone changes inferred to occur prior to or following *D. santomea* speciation in São Tomé. A common sexual attractant for *D. santomea* and *D. yakuba* males (7-T) generates the behavioral potential for unfit hybridization in sympatry due to F1 hybrid male sterility.^36^ *D. santomea* females evolve sexual aversion to cVA and *D. santomea* males reduce cVA, thereby maintaining efficient intraspecific mating while also maintaining reproductive isolation from *D. yakuba* (the two events cannot be definitively ordered). Reduced cVA and monomorphic 7-T release high intermale courtship in *D. santomea*. Reduced efficacy of cVA to promote aggression further suppresses fighting. Placement of *D. yakuba* secondary colonization after allopatric *D. santomea* speciation (instead of speciation in sympatry) from Cariou et al.^38^ See also Figure S7 and Tables S2, S3, and S4.

*D. santomea* has an interesting natural history^36,38,119^ (Figures 5A–5C). They are endemic to the equatorial West African island of São Tomé, located 280 km from Gabon in the Gulf of Guinea. *D. yakuba* also inhabits São Tomé, and the two sibling species largely segregate along the elevation gradient from the volcanic island’s warm, dry, and open perimeter (*D. yakuba*’s range) to its cool, humid, forested summit (*D. santomea*’s). A sympatric zone is formed at their mid-elevation boundary, where both species and rare F1 hybrids can be collected that adhere to Haldane’s rule for sterility in the heterogametic sex (i.e., males).^120^ Since *D. santomea* and *D. yakuba* males court females of both species promiscuously,^41,121^ male offspring sterility strongly pressures females to discriminate against heterospecific matings and maintain reproductive isolation.^28^ In flies this challenge would normally be expected to be met by females detecting species-specific acoustic features of male courtship song,^122^ but although *D. santomea* and *D. yakuba* songs are distinct,^65,67^ *D. santomea* females are still able to distinguish males of each species even if they are muted by wing amputation.^66^ These observations implicate additional species-identifying cues.

The cVA addition experiments above demonstrate that *D. santomea* males show increased courtship latency and reduced copulation rates in the presence of high cVA (Figures 3C and 3D), but we noticed that the effects may not derive exclusively from alterations to the males’ behavior. *D. santomea* females exhibited reduced innate attraction to cVA in a place preference assay compared with *D. melanogaster* (Figures S7A–S7I), and cVA exposure with courting males dramatically increased expression of ovipositor extrusion (Figures 5D and S7J), a sexual rejection behavior typically seen in recently mated, unreceptive *Drosophila* females.^124–127^ Significant rejection occurred at a lower cVA dose than was required to reduce male courtship (1 vs. 2 mg; Figures 5D vs. 3B), and the effect persisted even after normalization to the amount of male courtship observed (Figure 5E). Incorporating observations of wing spreading, a distinct female behavior signaling sexual acceptance,^128^ into a combined index of *D. santomea* receptivity also captured the cVA-dependent rejection response (Figure 5F). Strikingly, Or67d OSN silencing via ectopic expression of the inward rectifying potassium channel Kir2.1^129^ in *D. santomea* females paired with wild-type conspecific males resulted in ~2-fold increased copulation rates compared with controls (Figure 5G). Sensation of endogenous cVA on *D. santomea* males therefore normally limits *D. santomea* female sexual responses.

Thus, the effect of cVA to promote sexual receptivity in *D. melanogaster* females^90,130,131^ is reversed in *D. santomea*. This unexpected switch represents a third pheromonal change accrued in *D. santomea*, along with attraction to 7-T and reduced cVA abundance in males, relative to *D. melanogaster* and other *melanogaster* subgroup species. We hypothesize that the cVA valence reversal may help *D. santomea* females identify and avoid unwanted hybridization with sympatric, high cVA-expressing *D. yakuba* males (Figures 3A and S5A–S5G), thereby maintaining reproductive isolation in São Tomé (Figure 5H). A question arises then of whether the increased intermale courtship that results from cVA reduction is simply a costly but necessary accommodation by *D. santomea* males to the changing olfactory preferences of conspecific females. According to this view, SSB is inherently “non-reproductive” and may be detrimental to fitness or, at best, a waste of energy (an idea explained but not endorsed in Bailey and Zuk^42^). We next explored whether male-male courtship might have any adaptive value in *D. santomea*.

### P1^dsx^ activation or natural male-directed courtship induces a competitive mating advantage in *D. santomea* males

**Figure 6.**
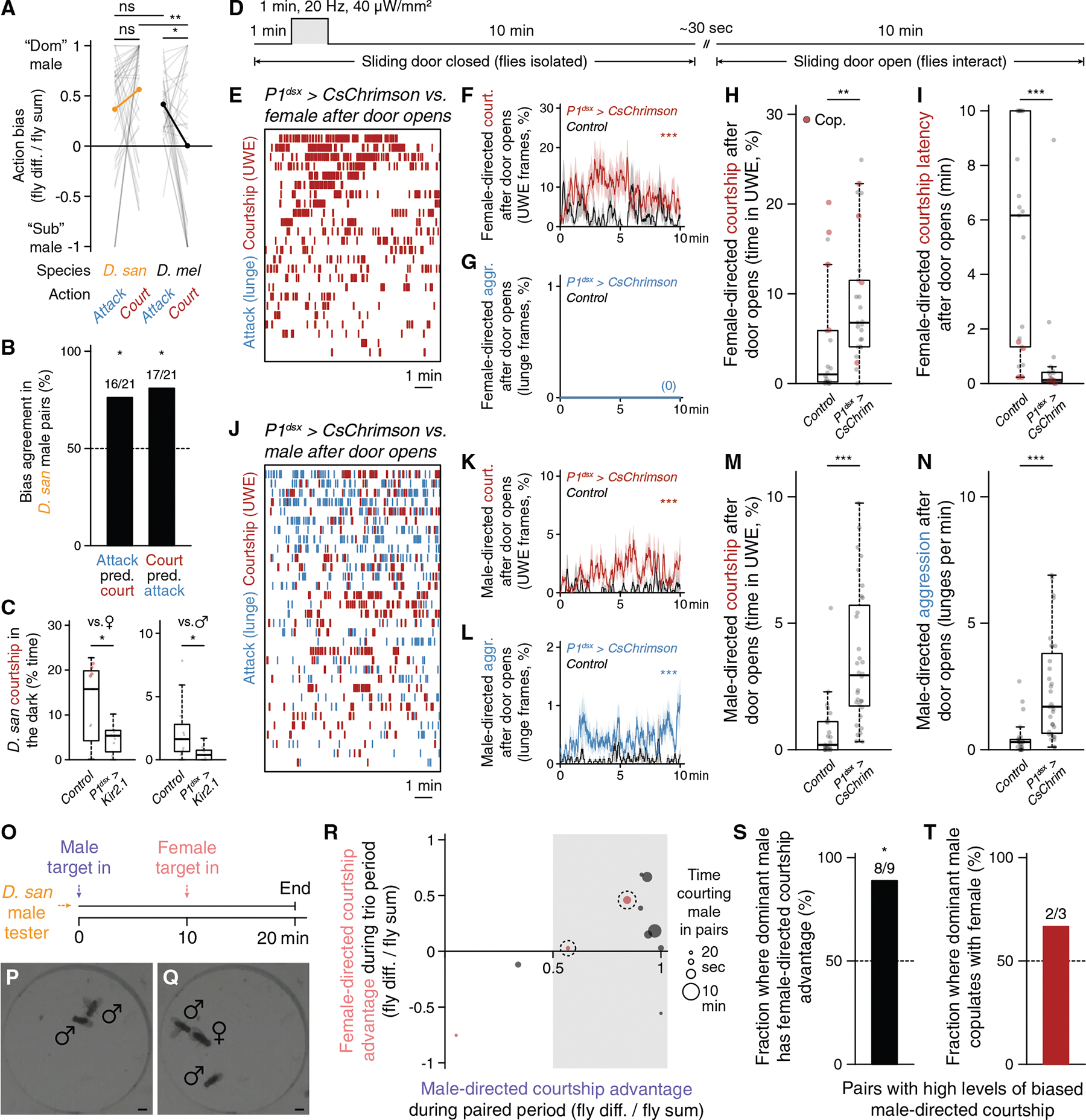
P1dsx activation or natural male-directed courtship induces a competitive mating advantage in D. santomea males (A) Spontaneous bias (“one-sidedness”) observed between flies in male-male *D. santomea* and *D. melanogaster* pairs for attack and courtship behaviors. Indices derived from the same fly pair are connected as lines, and distribution means are shown as paired orange (*D. santomea)* or black dots (*D. melanogaster*). Significance by paired (within species) or unpaired (across species) Mann-Whitney U tests. Note the high courtship bias in *D. santomea.* (B) Fraction of *D. santomea* male-male pairs with high attack bias where the same fly also showed a majority of courtship (left), or with high courtship bias where the same fly also showed a majority of attack (right). Significance compared with random chance (50%) by binomial tests. (C) Courtship in the dark by single-housed *D. santomea* males carrying *P1*^*dsx*^ split-Gal4 and Gal4-dependent Kir2.1 or a genetic control (males missing *dsx*^*AD*^) paired with group-housed wild-type conspecific female (left) or male targets (right). Red dots, males that copulated (only observed in controls). Significant courtship reduction in *P1*^*dsx*^ tester males toward both sexes by Mann-Whitney U tests. (D) Scheme for assessing persistent behavioral effects of *D. santomea P1*^*dsx*^ split-Gal4 activation. After a 1 min baseline, isolated *P1*^*dsx*^ tester males or genetic (non-activated) controls experience 1 min PS and a 10 min delay. Dividers (“sliding doors”) are then removed to allow paired flies to interact spontaneously for an additional 10 min. Pairings are between a *P1*^*dsx*^ or control tester male and either a wild-type conspecific female (E–I) or male group-housed partner (J–N). (E) Behavior rasters for *P1*^*dsx*^ tester males paired with wild-type conspecific females during the 10 min interaction phase. Red, courtship (UWE); blue, attack (lunge, none observed). (F–I) Expressivity and temporal dynamics of female-directed courtship (F, H, and I) and aggression (G) by *P1*^*dsx*^ tester males compared with genetic controls during the interaction phase. Means shown in (F) and (G) with SEM envelopes. Note the increased courtship expression (F and H) and decreased courtship latency (I) without any coincidence of attack. Significance by Kolmogorov-Smirnov (F and G) or Mann-Whitney U tests (H and I). (J–N) Behavior rasters (J), temporal dynamics (K and L), and expressivity of male-directed courtship (M) and aggression (N) by *P1*^*dsx*^ tester males compared with genetic controls during the interaction phase. Means shown in (K) and (L) with SEM envelopes. Note the significant increases for both social actions. (O) Scheme for determining a sexual priming effect of male-directed courtship. Two single-housed wild-type *D. santomea* males are paired for 10 min before a wild-type female is added for an additional 10 min. Male-directed courtship dominance is measured, and the identity of the dominant courter is ascertained in the paired period and then compared with female-directed courtship dominance in the trio. (P and Q) Video stills of a male courting another male first in the paired period (P) and then courting a female in the trio (Q). Scale bars, 1 mm. (R) Quantification of the sexual priming effect (O–Q). Female-directed courtship dominance in later trios (*y* axis) is plotted as a function of male-directed courtship dominance in earlier pairs (*x* axis). Points above the *x* axis represent cases where the same male exhibited male-directed courtship dominance in pairs and female-directed courtship dominance in trios. Dot size (scale on right) represents the total amount of male-directed courtship in pairs. Data were filtered for pairs with at least 20 s of courtship during the male-male phase, no matter the bias. Red dots, males that copulated. Gray box, region reflecting cases where males showed high levels of courtship dominance in pairs. Note the enrichment of points above the *x* axis in cases of high male-male courtship dominance. (S and T) Categorization of effects in (R) on female-directed courtship dominance (S) and copulation advantage (T). (S) All but one male exhibiting male-directed courtship dominance in pairs (89%, 8 of 9) also exhibited female-directed courtship dominance in trios (significant by binomial test). (T) Two out of three copulations observed were by the male exhibiting male-directed courtship dominance in pairs (circled red dots in R). The exception was from a “submissive” male in which courtship bias was very weak (uncircled red dot at bottom left in R). See also Figures S8–S10 and Tables S2, S3, and S4.

In our original comparative screen and various additional experiments, paired *D. santomea* males were derived from the same stock and prepared for dyadic behavior assays identically but nevertheless exhibited a surprising degree of courtship bias (one-sidedness) between members of the pair (Figures 6A and S8). That is, it was usually the case that just one male performed most of the total courtship observed, similar to biases observed during intermale aggression in *D. melanogaster*.^55,56,132,57^ In pairs where fighting also took place, the two actions were most often attributable to the same fly (Figure 6B), hinting that biased courtship and attack might both contribute to agonistic social dominance in *D. santomea*.

*D. santomea* males do not appear to be territorial over food in the laboratory (Figure S9), but we reasoned that the dominant courting male in dyadic pairs might be afforded a courtship advantage in subsequent female encounters due to persistent social arousal.^32^ Given that activity in homologous P1^dsx^ neurons is required for both female- and male-directed courtship in *D. santomea* (Figure 6C), we first confirmed that the long-lasting, behaviorally latent internal state that is evoked by transient activation of P1/pC1 neurons in *D. melanogaster*^54,133–135^ is conserved in *D. santomea* (Figure S10A). Persistent effects of transient P1^dsx^ photoactivation on later social behavior were assessed using a delayed interaction (“sliding door”) assay (Figure 6D). *D. santomea* P1^dsx^ tester or genetic control males were loaded into behavior chambers on the opposite side of a removable barrier from a wild-type target conspecific female or male made passive by group housing. A 1-min P1^dsx^ photostimulation was presented during the initial isolation phase, during which solitary tester males exhibited stimulus-locked UWE (Figures S10B–S10E). Barriers were removed 10 min following stimulation offset, after which paired tester and target flies were allowed to interact for an additional 10 min. P1^dsx^ tester males showed vigorous courtship, but not aggression, upon encountering target females, exceeding that observed in controls and with greatly reduced latency to initiation (Figures 6E–6I and S10F). When encountering a target male, P1^dsx^ tester males exhibited a mixture of courtship and aggression at levels that both also exceeded the controls (Figures 6J–6N and S10G). Thus, brief P1^dsx^ photoactivation induces a persistent internal state in *D. santomea* that can enhance subsequent social interactions for at least a period of multiple minutes, resulting in courtship to both sexes but aggression exclusively to males. This further supports that *D. santomea* males can distinguish conspecific sexes and adjust social behaviors accordingly even when highly aroused.

Next, we investigated whether a naturalistic male-male social interaction, rather than optogenetic P1^dsx^ activation, would induce a persistent arousal state transferrable to subsequent interactions with females (i.e., a “priming” effect). Wild-type tester males were exposed to two consecutive social encounters: first in a pair with another male, during which one-sided courtship was often performed by one of the males, and second in a trio with the same two males and a newly added female (Figures 6O–6Q). Courtship bouts by the two males were tracked in both periods (accounting for target sex in trios) to assess whether dominance in male-directed courtship during the first encounter would translate to dominance in female-directed courtship in the second. *D. santomea* males that dominated courtship in the paired male setting exhibited a significant courtship advantage toward females in the trio phase of the experiment, compared with their sub-dominant male partners (Figures 6R and 6S). Copulations were rare (3/9 trios), but two of the three observed were by the previously dominant male (Figure 6T). (In the third case, the male pair exhibited very weak dominance.) Thus, an episode of male-directed courtship in *D. santomea* increases the likelihood of courtship with a subsequently encountered female, suggesting a carry-over effect of a state of P1^dsx^-evoked social arousal that generalizes to mating behavior with both sexes.

### Convergent social behaviors and pheromones in *D. persimilis* males

**Figure 7.**
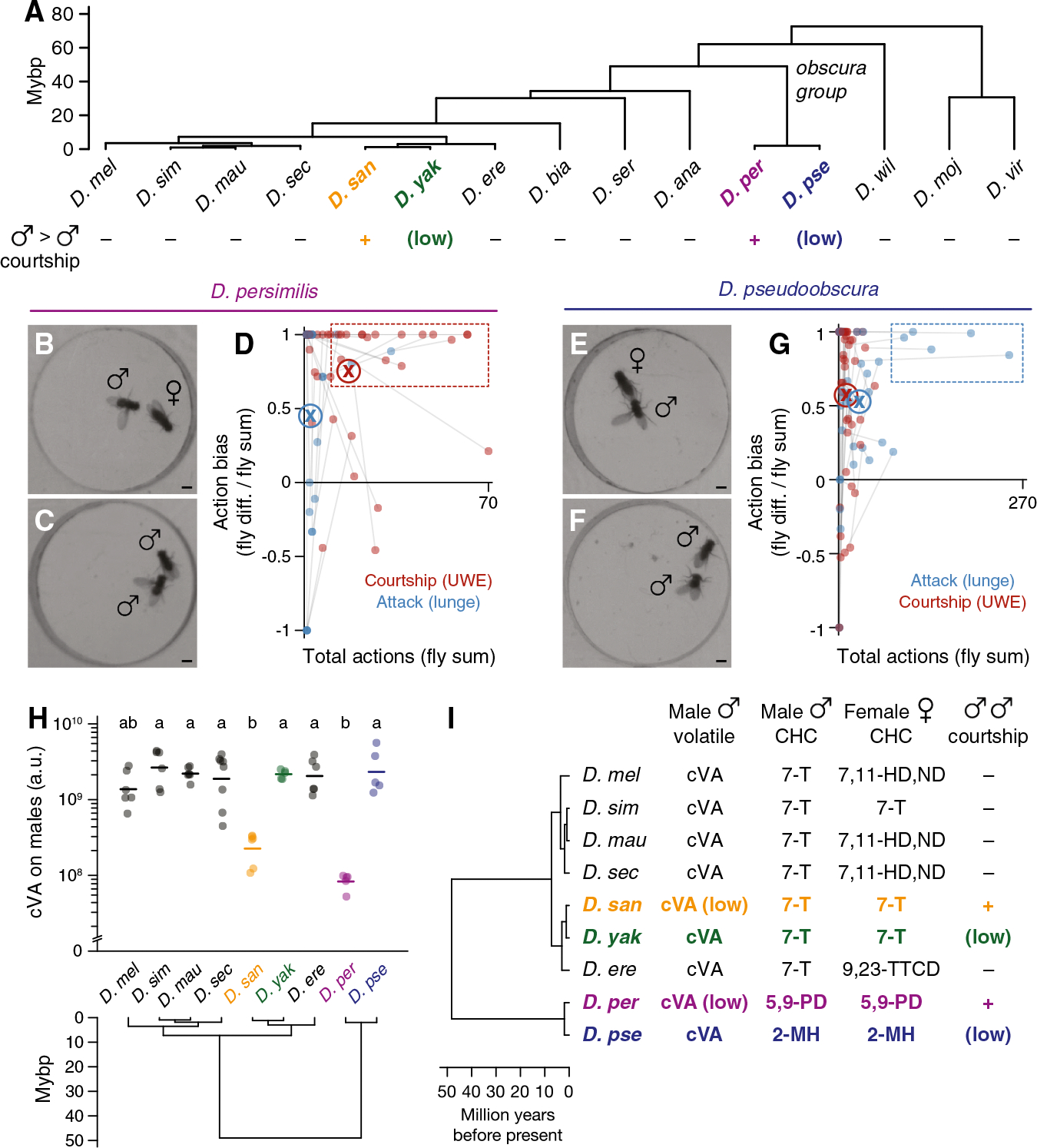
Convergent social behaviors and pheromones in *D. persimilis* males (A) Summarized indications of male-male courtship presence or absence in *Drosophila* species arranged by phylogeny. Note the sibling species *D. persimilis* (purple) and *D. pseudoobscura* (blue) in the obscura group with a similar behavioral pattern to *D. santomea*/*D. yakuba*. (B and C) Video stills of *D. persimilis* males courting conspecific female (B) and male targets (C). Images selected to represent species-typical action selection. Scale bars, 1 mm. (D) Social action biases in *D. persimilis* male-male pairs. Male-directed courtship (red dots) is more common than attack (blue dots) and often shows a strong bias to one fly in the pair (boxed region). Attack and courtship measurements from the same pair connected by thin lines. Circled X indicates the mean value for the color-coded action. (E–G) Video stills (E and F) and social action biases (G) for *D. pseudoobscura*. Note the female-directed courtship (E) and biased male-directed attack (F and G). Scale bars, 1 mm. (H) cVA abundance on males of the *melanogaster* subgroup and *D. persimilis*/*D. pseudoobscura* by TD-GC-MS.^70^ Statistical groupings by Dunn’s tests; a.u., arbitrary units. cVA levels are low in *D. persimilis* and high in *D. pseudoobscura*, as in *D. santomea*/*D. yakuba*. (I) Summary of relevant pheromones and social behaviors for the *melanogaster* subgroup (excluding *D. teissieri* and *D. orena*) and the *D. persimilis*/*D. pseudoobscura* sibling species pair. CHC monomorphism is common to multiple species, but low cVA abundance and high male-male courtship are selectively shared by *D. santomea* (orange) and *D. persimilis* (purple). Information compiled from this study and prior sources.^70,81,139^ See also Figure S11 and Tables S2 and S4.

Our behavioral screen also uncovered a second species highly prone to intermale courtship, *D. persimilis* (Figures 1B and S1A). *D. persimilis* and *D. santomea* last shared a common ancestor ~50 million years ago, and their natural ranges are ~13,000 km apart (São Tomé and western North America, respectively), but they share affinities for cool, high-elevation, montane regions, and each is a member of a sibling species pair with which they geographically overlap.^36,38,39^ Intermale courtship appears to be selectively derived in both taxa, since neither sibling species shows comparable SSB expression (Figure 7A). Importantly, males of *D. persimilis* and *D. pseudoobscura* (*D. persimilis*’s sibling) both court females interspecifically, but females show strong discrimination against hybridization, including with wingless males.^40,136,137^ *D. persimilis* male-directed courtship also shares visual resemblance with the female-directed form and is most often biased to just one male within each pair (Figures 7B–7D, S11A, and S11C–S11F). *D. pseudoobscura* males instead show the more genus-typical social patterns of male-directed aggression and female-directed courtship^138^ (Figures 7E–7G and S11B–S11E). Finally, like *D. santomea*, *D. persimilis* males have converged on the two key pheromonal features of CHC monomorphism^70,139^ and low cVA expected to reduce or eliminate major chemical barriers to male-male courtship (Figures 7H and 7I). Similar phylogenies and natural histories therefore correlate with convergent pheromonal signaling and high male SSB expression in two distantly related and geographically separated drosophilids.

## DISCUSSION

Here, we identify a *Drosophila* species that exhibits spontaneous, vigorous, and predominant SSB among males, despite working sex recognition and the capacity for aggression. We uncover three evolutionary changes in pheromone signaling (relative to *D. melanogaster*), which in combination elucidate a proximal mechanism of SSB emergence. First, *D. santomea* males, like their *D. yakuba* sibling,^26^ exhibit monomorphic expression and sexual attraction to 7-T, rather than repulsion.^71–74,76–80^ Second, *D. santomea* males exhibit a ten-fold reduction relative to *D. yakuba* and other males of the *melanogaster* subgroup in abundance of the male-specific anti-aphrodisiac olfactory pheromone cVA.^80,90–94^ Third, *D. santomea* females exhibit a behavioral valence switch in their cVA response from increased^90,130,131^ to decreased sexual receptivity. Together, the combined 7-T and cVA changes can explain selective amplification of *D. santomea* intermale courtship at the expense of aggression. Our results illustrate how multiple switches in the behavioral valence or abundance of pheromones in both sexes can collectively drive rapid intrasexual social evolution, culminating in highly penetrant male SSB. Although definitive evolutionary causes for these pheromonal changes are difficult to resolve retrospectively, strong selection pressure on *D. santomea* females to maintain reproductive isolation from *D. yakuba* in their overlapping habitat in São Tomé (West Africa)^36,38,119^ offers a unifying and plausible ultimate explanation for their appearance and fixation. A similar pheromonal pattern and high SSB expression are also evident in *D. persimilis* from North America, whose females must ensure reproductive isolation from the sympatric sibling species *D. pseudoobscura*.^39^ Thus, we identify convergent evolution of pheromonal and behavioral responses to similar selection pressures in two distant *Drosophila* species.

### A layered evolutionary path to elevated intermale courtship in *D. santomea*

*D. melanogaster* males^48^ and males of most *Drosophila* species surveyed here exhibit primarily female-directed courtship and male-directed aggression (though increased male-male courtship is evident in certain *D. melanogaster* mutants,^37^ strains,^140^ and experimental paradigms^141,142^). The sex specificity of these social behaviors in dimorphic *D. melanogaster* is controlled in part by male-specific 7-T and cVA, which cooperate to suppress intermale courtship and promote aggression.^73,76,80^ However, *D. santomea* and *D. yakuba* are monomorphic for 7-T,^68–70^ and males show 7-T sexual attraction rather than aversion.^26^ Despite high 7-T abundance in both sexes, intermale sexual behaviors in most monomorphic species (including *D. yakuba*) are minimal.^81^ *D. santomea* is the exception, and the key pheromonal difference which distinguishes its males from other *melanogaster* subgroup species is a selective and pronounced reduction in the abundance of cVA, an olfactory cue that in *D. melanogaster* suppresses male courtship.^80,90–94^ *D. santomea* male courtship behavior can be suppressed either by the addition of exogenous cVA or by direct photoactivation of OSNs expressing the cVA receptor Or67d,^90,96–98^ arguing that this correlation indeed reflects causation. Thus, the high level of courtship behavior among *D. santomea* males can be explained by two evolutionary changes in distinct cuticular and olfactory pheromone signaling pathways: a switch in the behavioral valence of 7-T (shared with *D. yakuba*) and a reduction in the abundance of cVA (specific to *D. santomea*).

A potential evolutionary explanation for male cVA reduction in *D. santomea* was lacking until our unexpected discovery of a second sexual valence switch (and third pheromonal change in total), this time in the behavioral response elicited by cVA in females from promoting^90,130,131^ to inhibiting mating receptivity. The switch may be related to interspecific sexual dynamics between *D. santomea* and *D. yakuba* at their geographic boundary in São Tomé. *D. yakuba* males court *D. santomea* females vigorously,^41,121^ and rare (sterile) F1 hybrid males can be collected within the natural sympatric zone.^36,38,119^ Thus, we speculate that the *D. santomea* female cVA valence switch arose due to ongoing selection pressure to maintain reproductive isolation from *D. yakuba*. If this explanation is correct, it would imply that *D. santomea* females use the high level of cVA on *D. yakuba* males as a sex- and species-identifying cue to ward off unwanted advances from heterospecific suitors (though it is possible that *D. yakuba* courtship song is also used by *D. santomea* females for species discrimination^65–67^). This female aversion to cVA would have led in turn to reduced cVA production by *D. santomea* males under sexual selection pressure.^143^

Two other evolutionary scenarios are also possible. *D. santomea* males may have initially reduced cVA abundance due to neutral drift or to pheromonally distinguish themselves from *D. yakuba* males,^144^ which in turn may have pressured *D. santomea* females to develop cVA aversion by sexual selection operating in the reverse direction. This seems less likely, however, because a reduction in cVA on *D. santomea* males without a concomitant valence switch in females might have temporarily favored cross-species hybridization with *D. yakuba*. Alternatively, *D. santomea* female cVA aversion and male cVA reduction may have evolved synchronously, potentially via genetic linkage.^145^

In any of these temporal scenarios, the ultimate outcome would still be an elegant pheromonal mechanism that maintains efficient intraspecific mating in *D. santomea* while minimizing unfit hybridization with *D. yakuba*. Interestingly, a similar sexual reversal from cVA promoting to inhibiting female receptivity has been reported in some other *Drosophila* species in which males do not produce cVA,^70^ including the agricultural pest *D. suzukii*.^146^ The evolutionary circumstances and selection pressures leading to cVA changes in those species are unclear, however, as are potential consequences for intermale social behaviors, including SSB.

Mechanistic explanations for the changes to cVA signaling in *D. santomea* females remain to be elucidated. In *D. suzukii*, female cVA valence reversal correlates with dramatic size reduction of the olfactory glomerulus targeted by Or67d OSNs (DA1),^146^ but this mechanism does not seem to be shared with *D. santomea*, where DA1 size and position appear similar to closely related species.^100^ In *D. melanogaster*, cVA promotes female receptivity through activation of DA1-lvPN^88^ and pCd neurons^147^ in the central brain. Whether behavioral valence inversion in *D. santomea* females is reflected in a physiological inversion of the effect of cVA on these neurons, from excitatory to inhibitory, is an interesting question for further investigation. A challenge also remains in resolving the genetic and biochemical nature of reduced cVA abundance in males and determining whether the divergent *D. santomea* and *D. persimilis* lineages achieved cVA reductions by similar means. A biosynthetic logic for cVA production in the male ejaculatory bulb has been proposed^82^ and one elongase identified,^148^ but additional enzymes necessary to generate cVA from fatty acid precursors are not known. *Cyp6a20* is a potential degradative enzyme for cVA, which is expressed by non-neuronal cells in Or67d-expressing sensillae (at1)^149,150^ and may be another target of selection.

### Mechanisms for suppressing aggression in *D. santomea* males

*D. santomea* males show little spontaneous attack. However, photoactivation of aggression-promoting neural circuits in the central brain is sufficient to elicit multiple contact- or non-contact-mediated agonistic actions (e.g., threat displays, lunging, and tussling) with supernormal intensity, ruling out a lack of vigor or selective atrophy of central aggression circuitry as explanations for the low level of spontaneous aggressive behavior. Because *D. santomea* males can recognize conspecific sexes, they must be able to detect male- and/or female-specific cues. Evidently, male-specific aggression-promoting cues (including cVA^86,87^) are either insufficient to activate aggression circuits or their action is overridden by coincident male-derived courtship-promoting cues (e.g., 7-T). Notably, *D. santomea* males only attack each other during the light-off periods of phasic P1^dsx^ photoactivation experiments, as also observed in *D. melanogaster*.^54,105^ This OFF-activation pattern of aggression may reflect a post-inhibitory rebound mechanism following ON-activation courtship, perhaps resulting from reciprocal inhibition between courtship and aggression circuits.^32,104^ Apparently the effects of this rebound are long lasting, since the propensity for male-directed attack remains elevated even 10 min after P1^dsx^ neurons have been stimulated in a delayed interaction (sliding door) assay, as initially demonstrated in *D. melanogaster*.^54,134^ One key difference is apparent, however: unlike *D. melanogaster* males, which exclusively fight following P1/pC1 activation, *D. santomea* males also court the conspecific males they subsequently encounter. High 7-T and low cVA, or other sexually attractive male cues, may therefore occasionally override rebound aggression, switching the persistent effect of P1/pC1 (or P1^dsx^) activation on social arousal^54,133–135^ from promoting aggression to courtship. This interpretation is supported by the fact that no aggression and instead exclusive courtship is performed toward females in the same assay.

In addition to inhibiting male courtship, cVA and Or67d signaling can promote aggression among *D. melanogaster* males.^86–89^ If this were also the case in *D. santomea*, then cVA reduction alone could have parsimoniously explained both the elevated courtship and reduced fighting observed in *D. santomea* male-male dyads. However, we have so far been unable to verify this prediction: various experimental efforts to elevate cVA, including strong and direct photoactivation of Or67d OSNs, have been ineffective at increasing *D. santomea* aggression (although effective at suppressing intermale courtship). A possible explanation for these negative results is suggested by the established hierarchical control of male-male social behavior by 7-T and cVA in *D. melanogaster*.^76^ There, cVA can promote fighting but only if 7-T signaling remains intact. In flies lacking 7-T or the gustatory receptor Gr32a, cVA has no effect to promote aggression or to inhibit the extensive male-male courtship unmasked by genetic disruption of 7-T signaling. Thus, suppression of intermale courtship by 7-T is apparently a prerequisite for the aggression-promoting effect of cVA. By extension, since in *D. santomea* 7-T no longer suppresses courtship, then according to this hierarchical model, increasing cVA experimentally should not be able to promote fighting, as is observed. This hypothesis does not exclude the possibility that thus-far undetected evolutionary changes in aggression-promoting components of cVA central processing circuitry^88,104,134^ may also prevent this pheromone from promoting aggression in *D. santomea* males.

### Recent speciation and intermale sexual behavior

It seems unlikely to be a coincidence that the two cases of enhanced male SSB discovered here in *Drosophila* are each observed in members of sympatric sibling species pairs susceptible to unfit hybridization. This suggests that recent speciation may promote high SSB expression (or alternatively that high SSB expression may promote speciation), especially in the evolutionarily “younger” species inferred to be derived due to its more restricted geographic range. Interestingly, a significant negative correlation between SSB expression and taxon age was found in a phylogenetic analysis across all mammals, including the Hominidae.^151^ Our findings provide a general model that may explain at least some of these cases where sympatry and hybridization are features of sibling species’ natural history.^152,153^ Namely, where sympatry necessitates enforcement of reproductive isolation between interfertile species that produce unfit hybrid offspring^28^ (such as sterile F1 males), mechanisms that evolve to prevent interspecific mating in one sex may alter pheromone signaling pathways in a manner that also enhances SSB in the other. This route would be particularly accessible when single pheromones play dual roles to both promote mating between sexes and suppress mating within a sex, as cVA does in *Drosophila* males.^90^ Even in species that do not appear to rely heavily on pheromone signaling, the imperative of reproductive isolation may still release SSB by modifying other key social signals (e.g., visual appearance, song acoustics, movement patterns, hormone levels, etc.) that influence sexual behavior.^154^ From this perspective, SSB emergence may represent a form of “evolutionary hitchhiking”^155^ due to its close functional coupling to reproductive isolation during speciation.

Emergence by hitchhiking does not imply, however, that SSB is necessarily nonadaptive. *D. melanogaster* males robustly court very immature males^156,157^ (within a few hours of eclosion), which express lower amounts of cVA than adults.^158,159^ It has been suggested that receiving courtship attempts may help prepare young males to court females more intensely later in life.^160,161^ In *D. santomea*, an adaptive value of SSB expression among mature males may have accrued for either of two reasons, not mutually exclusive. First, we observe that males performing naturalistic male-male courtship (or courtship evoked by P1^dsx^ photostimulation) initiate courtship more quickly and sustain it more vigorously upon subsequently encountering a female. Males competing for female mates could therefore benefit from this persistent social arousal^54,133–135^ or priming effect. Alternatively, the strong courtship bias (one-sidedness) observed in dyadic male-male interactions and the fact that the same individual courting fly is also the one more likely to attack suggest that intermale courtship could have an adaptive function in mediating social dominance.^162–164^ In that case, *D. santomea* males may have evolved a social strategy in which the usual adaptive functions of aggression can instead be achieved by courtship dominance through male SSB, avoiding aggression’s injurious costs while maintaining its core competitive intent.^165^ Which of these explanations holds true for *D. santomea* and *D. persimilis*, and whether this logic may apply to other species more closely related to humans^166,167^ or to humans themselves,^168,169^ are compelling questions for future investigation.

### Limitations of the study

Our behavioral screen was conducted using standardized dyadic conditions and controlled environmental variables (temperature, humidity, chamber design, etc.), optimized to elicit aggression in *D. melanogaster* in a laboratory setting.^54,170^ Alternative laboratory or especially natural field conditions might therefore generate different results. The data implicating 7-T as a male aphrodisiac involve the addition of exogenous pheromones to dead females. Therefore, we cannot exclude that other components of the CHC bouquet and/or non-CHC pheromones contribute to *D. santomea* male sexual attraction to live conspecific females or males or that non-chemosensory signals also play a role.^24^ We have also so far been unable to demonstrate by calcium imaging that 7-T application, or contact with conspecific females or males, induces reliable physiological activation of P1^dsx^ somata or neuropil in restrained *D. santomea* males monitored by two-photon microscopy. We believe these negative results likely reflect technical issues rather than biology, since both 7-T and conspecific females carrying high endogenous levels of 7-T can activate P1^dsx^ neuropil in the sibling species *D. yakuba*.^26^ Finally, our evidence that cVA inhibits *D. santomea* male courtship derives from gain-of-function experiments involving exogenous cVA addition or optogenetic activation of Or67d OSNs, all of which are vulnerable to behavioral outcomes resulting from supraphysiological pheromone exposure (whether chemical or fictive). As discussed elsewhere,^86^ cVA pheromonal signaling may have distinct effects on courtship or other social behaviors at lower concentrations or under weaker activation conditions.

Following acceptance of our manuscript, a study from Green et al.^171^ was published demonstrating that male SSB is common in field crickets (*Gryllus* spp.) despite working sex recognition.

## RESOURCE AVAILABILITY

### Lead contact

Requests for further information and resources should be directed to and will be fulfilled by the lead contact, David Anderson (wuwei@caltech.edu).

### Materials availability

All constructs and fly strains generated in this study are available from the lead contact without restriction.

### Data and code availability

- All data have been deposited at the Caltech Research Data Repository (https://data.caltech.edu) and are publicly available as of the date of publication at https://doi.org/10.22002/f1q3g-q5r74.
- This paper does not report original code.
- Any additional information required to reanalyze the data reported in this paper is available from the lead contact upon request.

## STAR★METHODS

Detailed methods are provided in the online version of this paper and include the following:

### EXPERIMENTAL MODEL AND STUDY PARTICIPANT DETAILS

#### Fly stocks

All species, stocks, and crosses were reared at 24–25C and 50% humidity on a 12h:12h light cycle with standard Caltech fly media. Details about the flies used in each experiment (species, strain, sex, genetic background, and experimental and control genotypes) are listed in Tables S2 and S3. Table S2 provides identifiers for the 21 wildtype strains utilized and lists their usage by figure. Table S3 provides similar information for *D. santomea* transgenic lines. All wildtype strains were obtained from the National *Drosophila* Species Stock Center (Cornell College of Agriculture and Life Science, Ithaca, NY) except *D. melanogaster* Canton-S (Anderson lab, Caltech) and three *D. santomea* strains (David Stern lab, Janelia Research Campus).

### METHOD DETAILS

#### Generation of transgenic and knock-in alleles in *D. santomea*

Transgenic *D. santomea* driver and hemidriver lines using enhancer fragments to regulate Gal4 expression were made using phiC31 recombination^114^ into existing attP landing sites.^115^ White-mutant *D. santomea* attP sites 2251 (2R) and 2253 (3R) were targeted using Gal4 (pBPGUw), Gal4DBD (pBPZpGAL4DBDUw), or p65AD (pBPp65ADZpUw) vectors carrying an attB recognition site and mini-white selection marker.^101,172^ When available, ready-made constructs with desired enhancer sequences were obtained from Janelia. Otherwise enhancer sequences were obtained from Pfeiffer et al.,^172^ synthesized by Twist Bioscience (South San Francisco, CA) with flanking attL1/L2 recognition sites, and cloned upstream of transcriptional control cassettes in the backbone vectors using Gateway LR reactions (Thermo Fisher Scientific, Waltham, MA). Assembled constructs were verified by sequencing and restriction digest, maxi-prepped using endotoxin-free kits, and cleaned using ethanol precipitation before sending for injection. Rainbow Transgenic Flies, Inc. (Camarillo, CA) generated injection mixes containing the construct and phiC31 integrase mRNA (and sometimes also integrase plasmid to increase integration efficiency) and performed injections into *D. santomea* eggs in batches of 200–250 per desired transformant. Viable G0 (injected) flies were collected and crossed to a white-mutant strain^115^ (*2041/w-[3.2]*) before screening for yellow-eyed (transgene heterozygous) flies in the G1 progeny to confirm germline transmission. Heterozygotes were crossed together to generate transgene homozygotes as stable lines used for experiments or additional crosses.

Knock-in driver lines were generated using CRISPR/Cas9-mediated homology-directed repair (HDR).^112^ Donor construct assembly and sgRNA selection for *D. santomea dsx-p65AD* were performed previously^113^ but transformants positive for the selection marker (*MHC-DsRed*) had not been obtained. The same reagents were re-injected into *D. santomea 146/STO-CAGO 1482* (David Stern, Janelia) in a mixture with nls-Cas9-nls protein (PNA Bio, Thousand Oaks, CA) and one transformant with germline transmission of abdominal mCherry was successfully recovered. Crosses among G1 progeny yielded G2 flies with germline transmission of the transgene and the expected *dsx* genital phenotype, verifying the genomic insertion.

*D. santomea Or67d*^*Gal4*^ was generated using the Atalanta strategy.^173^ A backbone vector with two U6 promoters and an “empty payload” (pJAT32) was selected as the Gateway acceptor. Two donor sequences were synthesized by Twist Bioscience and cloned into the acceptor in a simultaneous double-Gateway reaction. The first (flanked by attL3/L4) is a *T2A-Gal4-stop(hsp70)* cassette (*Gal4-stop(hsp70)* from pBPGUw) designed to be inserted downstream from and as near as possible to the *D. santomea Or67d (3L:LOC120447699)* translational start codon. Placement of the adjacent homology arm ensures that insertion occurs in-frame with the residual 15 coding base pairs so that Gal4 is properly translated. The second (flanked by attL5/L6) is an *ie1-mCherry-stop(p10)* selection marker^26^ with inverted orientation relative to Gal4. Both donors also contain a 1 kb homology arm and U6-tRNA-sgRNA-tRNA array which, following LR recombination, are positioned downstream of each U6 promoter. Candidate sgRNAs were designed in Geneious Prime (Auckland, NZ) and a pair was selected which excises 99% of *Or67d* coding sequence, resulting in a null allele (upstream *AATGGCAAAAGTTGAGCCCG*, downstream *ACTGCTGGTTTCCAAAGGAG*). Successful Gateway products were verified by restriction digest and tiled sequencing. Injection into *D. santomea 146/STO-CAGO 1482* was performed by Rainbow Transgenic Flies using nls-Cas9-nls protein as the enzyme source and G1 progeny were screened for germline-transmitting abdominal mCherry. Multiple independent insertion lines were recovered as G2s, one of which was used throughout after anatomical verification of uniglomerular labeling in the antennal lobes of the size and position expected for Or67d OSNs.^90,100^ Note that in this strategy Gal4 expression is controlled exclusively by the *Or67d* native genomic regulatory context rather than an exogenous promoter.

#### Spontaneous behavior experiments with video recording

Experimental crosses were seeded with 10–12 virgin females and 5–6 males and flipped every 3–4 days. Experimental flies were collected mostly as virgins on the day of eclosion and reared either in isolation (single-housed, 1 individual per vial) or in same-sex groups (group-housed, 10–20 individuals per vial) for 5–6 days until testing. Group-housed (but not single-housed) flies were flipped into fresh vials 1–2 days before testing. For experiments requiring unambiguous determination of individual identity (e.g., in mixed-genotype or mixed-species male-male pairs), a small distal portion of one fly’s wing was clipped using a razor blade under light anesthesia. Whenever possible, the “target” fly (individual whose behavior was not being measured) was selected for wing clipping. Otherwise clips were balanced between the two interacting species or genotypes. Females were collected and group-housed as were males (mostly as virgins on the day of eclosion, 10–20 individuals per vial) and age-matched to the males they were tested with as courtship targets. As this was usually a period of 5–6 days, it is likely that even the few females that were not virgins upon collection had by the time of the experiment shed residual male pheromones potentially transferred during prior copulation (e.g., cVA). Group-housed females were flipped 1–2 days before testing. Housing for each experiment arranged by figure can be found in Tables S2 and S3 (“single” or “group” under the “Housing” heading).

Behavior experiments took place in the morning between 9:00AM (ZT0) and 1:00PM (ZT4) in a temperature and humidity-controlled room (24C, 50% humidity) with ambient white lighting. Single, pairs, or trios of flies interacted in 8-well acrylic circular chambers (~2.0 cm^2^ surface area, ~2.4 cm^3^ volume) designed and described previously.^54,170^ Walls of the chambers were coated with Fluon (Tar Heel Ants, Raleigh, NC) and lids with a siliconizing fluid (Thermo Fisher) to deter flies from climbing. Chamber floors were covered with a thin layer of freshly prepared “food” (2.5% (w/v) sucrose, 2.25% (w/v) agarose in Tropicana apple juice) and illuminated with an 855 nm backlight (Smart Vision Lights, Muskegon, MI). Experiments testing alternative foods replaced the apple with other juices acquired commercially (*Morinda*, fig) or from botanical gardens (*Marula*, *Pandanus*), or with water as a “no food” condition (sucrose was also omitted in this case). Flies were introduced one at a time into chambers by gentle aspiration through a small hole in the lid. Interactions were video recorded from above using a Point Grey Flea3 camera running at 30 Hz with a 780 nm long pass IR filter (Midwest Optical Systems, Palatine, IL). Experiments performed in 8-well chambers are listed as “small” under the “Chamber” heading in Tables S2 and S3.

For copulation tests with Or67d OSN-silenced *D. santomea* females and territoriality assays with wildtype *D. santomea* males, “large” chambers consisting of a central food patch (apple juice agarose) and a neutral surround (bare acrylic) were used. Chambers measure 20 cm^2^ surface area and 120 cm^3^ volume, and the circular food patch ~4.9 cm^2^ (25% surface area). Flies (12 females and 12 males for copulation tests, 2 males for territoriality tests) were gently aspirated into chambers and video recorded using identical lighting and camera settings as for small chambers described above.

#### Optogenetic photoactivation experiments

Flies were collected on the day of eclosion into vials containing food supplemented with 400 μM all *trans*-Retinal (Millipore Sigma, Burlington, MA) and reared in the dark for 5–6 days, with one flip into fresh supplemented vials 1–2 days before testing. Photoactivated flies were usually group-housed (10–20 males per vial), except in male Or67d OSN courtship suppression experiments which were single-housed to encourage spontaneous courtship prior to stimulation. Group-housed males paired together during behavior assays came from different vials.

Experiments took place in the “small” chambers using an 8-LED array setup reported previously.^54,170^ LEDs delivered flashing 685 nm red light in photostimulation (PS) blocks with 10 ms pulse width, frequencies ranging from 1–40 Hz, and intensities from 10–260 μW/mm^2^, calibrated with a photodiode power sensor (S130VC, Thorlabs, Newton, NJ) placed at the location of the behavior chambers. Exact PS conditions (timing, frequency, intensity) for each experiment are indicated in figures and described in figure legends.

Delayed interaction (“sliding door”) experiments testing persistent behavioral responses after PS were performed in 8-well chambers fitted with vertical slits to allow a metal barrier to divide each chamber in half. Barriers remained in place during the isolation/PS phase of the experiment, which upon completion of the 10 min delay were manually removed during a ~30 s period while video recordings were paused. Video recordings resumed for the social interaction phase after the barriers were removed.

#### Behavior annotations and analysis

Annotations of aggressive (A) and courtship (C) behaviors in male-male pairs from each species for the primary screen were performed manually using action definitions from Eyjolfsdottir et al.^53^ (threat (A), charge (A), lunge (A), hold (A), tussle (A), unilateral wing extension (C), circle (C), copulation attempt (C)), Duistermars et al.^58^ (pump (A)), Cobb et al.^14^ (bilateral wing extension (C), row (C)), and Nilsen et al.^174^ (headbutt (A)). One additional action observed exclusively in *D. pseudoobscura* males, “*barrage*,” is defined as follows: “*the attacking fly pushes into and quickly chases the opponent while both wings are fully extended to 90 degrees and vibrating rapidly*.” This action appears to have been described once previously without being named.^138^ Ethogram nodes were constructed from action bout frequencies and ethogram edges from transition matrices using a 1 s cutoff.

For most experiments, video recordings were analyzed in an automated fashion by first tracking the absolute and relative positions of flies (including the body, legs, and wings) in the chamber at every frame (Caltech FlyTracker, https://kristinbranson.github.io/FlyTracker/),^53^ and using the tracking information as input to automated behavior classifiers developed previously for *D. melanogaster* social interactions^54^ implemented in JAABA (https://jaaba.sourceforge.net/).^52^ Two classifiers were used extensively after satisfactory performance evaluation for species within the *melanogaster* subgroup: “lunge” for consummatory, contact-mediated male aggression, and “unilateral wing extension” (UWE) for male courtship. Bouts from the UWE classifier were filtered to retain only those lasting at least 0.5 s (15 frames).^175^ Recordings analyzed in this way are listed as “automated” under the “Behavioral analysis” heading in Tables S2 and S3. Manually annotated recordings, including ones analyzed for the female sexual behaviors ovipositor extrusion^126,127^ and wing spreading,^128^ are instead labeled “manual.” For experiments involving a male-female pair, analysis ended at the point of copulation (if it occurred) and the latency to copulation was used for normalization procedures rather than the total recording time.

#### Courtship song recordings and analysis

Flies (*D. santomea* wildtype strain 14021–0271.00) were raised in standard laboratory conditions at 23C and 12h:12h light cycle. Sexually naïve individuals were isolated within 3 h of eclosion and single-housed for 5–7 days prior to behavioral analysis. Synchronized audio and video song recordings were made within 4 h of lights-on in the morning using the Song Torrent system.^64^ Individual flies arranged in male-male or male-female pairs were loaded onto opposite sides of a divider in recording chambers and introduced to each other simultaneously at the beginning of 30 min recordings.

Videos of 16 male-male and 16 male-female pairs of *D. santomea* flies were visually scanned and 3 pairs of each type with ample courtship were selected for audio analysis. Song traces were visualized and annotated in Tempo^67^ until dozens to hundreds of both “pulse” and “clack” events had been recorded for each courting male (instead of to saturation). The exact amplitude and position of each event’s peak was determined by obtaining the maximum signal intensity within a 20 ms time window centered on the annotation point. Events were assigned to being part of a “train” when their separation from another event of the same type was less than 200 ms for pulse and 400 ms for clack.

#### Pheromone applications

(Z)-11-*cis*-vaccenyl acetate (cVA) and (Z)-7-tricosene (7-T) were acquired commercially from Pherobank B.V. (Wijk bij Duurstede, NL) as neat liquid formulations. For behavioral experiments with cVA on filter paper, 0.5–2 μl neat formulation was applied just prior to introduction of flies into the chamber. For direct treatment of live flies, cVA neat formulation was diluted to a 10% working solution in acetone (179124, Millipore Sigma), from which 0.2 μl was pipetted onto the abdomen of target flies under anesthesia 1–2 hours prior to behavioral testing, as previously.^90^ Flies that did not rouse were discarded. For direct treatment of dead flies, 0.2 μl of 10% cVA (acetone) or 7-T (hexane) working solutions was directly pipetted onto the abdomen of flies freshly killed by freezing at −80C and glued to the chamber floor using melted agarose of the same “food” composition.

#### Odor place preference tests

The olfactory y-maze was constructed by laser cutting multiple layers of 1/8th inch 3143 infrared (IR)-transmitting acrylic (ACRY31430.125PM, ePlastics, San Diego, CA) to shape. The bottom floor layer was finely wet sanded for texture. Chambers at the end of each arm meant for odor reservoirs and a center port for the vacuum line were blocked off with fine mesh. Top layers consisted of a ceiling of static dissipating acrylic (8774K11, McMaster-Carr, Santa Fe Springs, CA) with a rim of IR acrylic to block ambient light, and a final roof layer with small holes above the chambers to allow air to be drawn into and down the arena by the vacuum line. The layers were supported and sealed with three threaded rods and bolts. Acrylic layers were periodically cleaned with 1% SDS, water, and isopropanol. The arena was illuminated from below using a custom-built IR 810 nm LED lighting board and diffused with semi-opaque white acrylic. Videos were collected with a machine vision camera (BFS-U3–04S2M-C: 0.4 MP, Teledyne FLIR) running at 45 Hz and a Pentax 12mm 1:1.2 TV lens (FL-HC1212B-VG, Ricoh).

Flies were collected on the day of eclosion, separated by sex, and single- or group-housed for one week until testing. For each trial, 5 μl odor (10% cVA in acetone or acetone control) was first loaded at the top of each arm, then 2–4 cold anesthetized males or females were aspirated into the central bowl and the lid quickly fastened. 10 min recordings initiated once the flies roused. Cumulative position traces were obtained by first median filtering on a subset of frames to construct a background image. OpenCV (4.6.0) was used to perform thresholding on background-subtracted frames. The resulting “blob detection” indicated animal position per frame and positions across the entire trial were plotted using Matplotlib (3.4.3). Normalized occupancy in the left vs. right arm was calculated from cumulative position traces using cutoffs including the two arms but excluding the central bowl.

#### Gas chromatography – mass spectrometry (GC-MS)

Flies were collected on the day of eclosion, separated by sex, and group-housed in sets of 10 for one week until analysis. Flies were anesthetized and transferred to 2 ml glass screw top vials (Thermo Fisher) containing 100 μl hexane (139386, Millipore Sigma) with 10 ng/μl octadecane (C18) internal standard (O652, Millipore Sigma). Hexane extraction was allowed to proceed for 15 min at room temperature, after which extracts were transferred to glass inserts (Ibis Scientific, Clark County, NV) positioned in new screw top vials for either immediate analysis or storage at −80C.

An AOC-2i autoinjector (Shimadzu, Kyoto, Japan) was used to inject 2 μl of each sample into a Shimadzu QP-2020 GC-MS system (Shimadzu, Kyoto, Japan) equipped with a ZB-5MS fused silica capillary column (30 m × 0.25 mmID, df=0.25) (Phenomenex, Torrance, CA) and helium as the carrier gas. The injection port was maintained at a temperature of 310C and was operated in splitless mode, with a column flow rate of 2.15 ml/min. The temperature program consisted of a 40C hold for 1 min, followed by a 20C/min ramp to 250C, a 5C/min ramp to 320C, and a 320C hold for 7.5 min. The transfer line to the mass spectrometer was held at 320C and the ion source temperature at 230C. Electron ionization was performed at 70 eV and MS scans were collected at 2 Hz, spanning 40–650 m/z. Identification of cuticular hydrocarbon (CHC) and ester compounds was determined by retention index, diagnostic ions, and comparison to previous GC-MS results from *Drosophila*.^68,69,176,177^ GC-MS spectra were integrated in python using the pyteomics package (v4.4.0).

#### Immunostaining and imaging

Whole brains were dissected in ice-cold phosphate-buffered saline (PBS) and fixed in 4% paraformaldehyde for 20 min at room temperature (RT). Samples were washed three times in 0.5% PBST (PBS containing 0.5% (v/v) Triton X-100) for 10 min at RT, then permeabilized in 2% PBST for 30 min at RT. Samples were blocked in 0.5% PBST with 5% normal goat serum (NGS) for 1 h at RT or overnight at 4C. Samples were incubated with primary antibodies in blocking solution for 48–72 h at 4C (rabbit anti-DsRed 1:1000, mouse anti-Bruchpilot 1:50) followed by three 10 min washes at RT in 0.5% PBST. Samples were incubated in secondary antibodies (anti-rabbit IgG AF568 and anti-mouse IgG AF633, 1:1000) in blocking solution for 36–48 h at 4C, then again washed three times in 0.5% PBST for 10 min at RT before mounting.

Samples were mounted in VectaShield antifade medium (Vector Laboratories, Newark, CA) and imaged on an Olympus Fluoview FV3000 confocal microscope (Tokyo, JP) with inverted 30X/1.05 or 60X/1.30 oil immersion objective. Image stacks were acquired at 1024 × 1024 px^2^ spatial resolution with 1.0–1.5 μm z-step intervals. Images throughout (referred to as “photomicrographs”) are maximum intensity projections (MIPs) of image stacks generated in Fiji. Fiji was also used to pseudocolor, overlay, adjust brightness and contrast, and add scale bars to images.

### QUANTIFICATION AND STATISTICAL ANALYSIS

Fly positional tracking, automated social behavior annotations, and song analyses were performed using software implemented in MATLAB (MathWorks, Natick, MA). Odor place preference and GC-MS experiments were analyzed in python using Jupyter Notebooks (https://jupyter.org/). The tabulated outputs of these analyses were saved and exported as text files for final analyses, statistical testing, and plotting in R (R Project for Statistical Computing) using RStudio (Posit PBC, Boston, MA). Illustrator 2025 (Adobe, San Jose, CA) was used to adjust aesthetic features of the plots and assemble figures.

Measurement distributions are often shown as boxplots highlighting the median and overlaid data points. Outliers are identified in the standard way (if > Q3+1.5*QR or if < Q1−1.5*QR) and visually excluded from box whiskers, but are retained in all quantifications (including statistical tests) and still shown as overlaid dots. Violin plots show full distribution ranges as vertically mirrored kernel density plots with the mean highlighted. Statistical tests consisted mostly of non-parametric comparisons between pairs or multiple distributions and were implemented in R. Mann-Whitney *U* tests (R base *stats* package: *wilcox.test* function) were used to compare pairs of distributions (two-sample) or an empirical distribution to a null median (one-sample), and could be paired or unpaired depending on whether the comparison involved the same individual or interacting pair. Two-sample Kolmogorov–Smirnov tests (*stats: ks.test*) were used to compare pairs of time-evolving distributions or cumulative distributions. Kruskal-Wallis tests (*stats: kruskal.test*) and post-hoc Dunn’s tests (*FSA: dunnTest*) were used to compare multiple distributions and assign lettered groupings. Binomial tests (*stats: binom.test*) were used to compare pairs of frequencies for binary outcomes (two-sample) or an empirical frequency to a null probability (one-sample). Linear fits and Pearson correlations were calculated using the *lm* and *cor* functions (*stats*), respectively. Benjamini-Hochberg multiple test correction was used to adjust p-values whenever appropriate (*stats: p.adjust: method=‘BH’*). Standardized effect sizes were calculated using Cohen’s *d* (*effsize: cohen.d*). Tests shown in each figure can be referenced in Table S4 for details including sample sizes, sample units, test used, whether correction was applied, adjusted p-value, and effect size. Significance levels are reported in figures as * <0.05, ** <0.01, and *** <0.001.

## Supplementary Material

1

2

3

4

SUPPLEMENTAL INFORMATION

Supplemental information can be found online at https://doi.org/10.1016/j.cub.2026.02.046.

## Figures and Tables

**KEY RESOURCES TABLE:** 

REAGENT or RESOURCE	SOURCE	IDENTIFIER

Antibodies		

anti-DsRed (rabbit)	Takara Bio	Cat#632496; RRID: AB_10013483
anti-Bruchpilot (mouse)	Developmental Studies Hybridoma Bank	Cat#nc82; RRID: AB_2314866
Goat anti-rabbit IgG (H+L) Alexa Fluor 568	Thermo Fisher Scientific	Cat#A-11011; RRID: AB_143157
Goat anti-mouse IgG (H+L) Alexa Fluor 633	Thermo Fisher Scientific	Cat#A-21050; RRID: AB_2535718

Biological samples		

Normal Goat Serum	Jackson ImmunoResearch Labs	Cat#005-000-121; RRID: AB_2336990
Tropicana apple juice	Amazon	Cat#B007SNJDXM
Virgin noni juice	Amazon	Cat#B082S4Q6C7
Caramelized fig syrup	Amazon	Cat#B0DHY7K9RP
Marula (*Sclerocarya birrea*)	Waimea Botanical Garden (O’ahu, HI)	N/A
Pandanus (*Pandanus furcatus*)	Huntington Botanical Garden (San Marino, CA)	N/A

Chemicals, peptides, and recombinant proteins		

Paraformaldehyde, 16% solution, EM grade	Electron Microscopy Sciences	Cat#15710 (30525-89-4)
Triton X-100	Millipore Sigma	Cat#X100 (9036-19-5)
VectaShield Antifade Mounting Medium	Vector Laboratories	Cat#H-1000; RRID: AB_2336789
all *trans*-Retinal	Millipore Sigma	Cat#R2500 (116-31-4)
Hydrocarbon Soluble Siliconizing Fluid	Thermo Fisher Scientific	Cat#TS-42800 (474-02-4)
Fluon (PTFE-30)	Tar Heel Ants	9002-84-0
Acetone	Millipore Sigma	Cat#179124 (67-64-1)
Hexane	Millipore Sigma	Cat#139386 (110-54-3)
Octadecane (C18)	Millipore Sigma	Cat#O652 (593-45-3)
(Z)-11-*cis*-vaccenyl acetate (cVA)	Pherobank B.V.	Cat#10421 (6186-98-7)
(Z)-7-Tricosene (7-T)	Pherobank B.V.	Cat#12457 (52078-57-6)
Cas9 with dual NLS	PNA Bio	Cat#CP01

Critical commercial assays		

Gateway LR Clonase II Enzyme mix	Thermo Fisher Scientific	Cat#11791020
NucleoBond Xtra Maxi EF	Takara Bio	Cat#740424

Deposited data		

*Drosophila* cVA abundance measurements (TD-GC-MS)	Mohammed Khallaf, Markus Knaden	10.1038/s41467-021-24395-z
*D. santomea* genome assembly (Prin_Dsan_1.1)	NCBI RefSeq	GCF_016746245.2

Experimental models: Organisms/strains		

*D. melanogaster*	David Anderson lab	Canton-S (Martin Heisenberg)
*D. simulans*	National *Drosophila* Species Stock Center	14021-0251.006
*D. mauritiana*	National *Drosophila* Species Stock Center	14021-0241.151
*D. sechellia*	National *Drosophila* Species Stock Center	14021-0248.28
*D. santomea*	National *Drosophila* Species Stock Center	14021-0271.00
*D. santomea*	David Stern	146/STO-CAGO 1482
*D. santomea*	David Stern	148/STO.7
*D. santomea*	David Stern	151/STO.6
*D. yakuba*	National *Drosophila* Species Stock Center	14021-0261.00
*D. erecta*	National *Drosophila* Species Stock Center	14021-0224.01
*D. biarmipes*	National *Drosophila* Species Stock Center	14023-0361.03
*D. serrata*	National *Drosophila* Species Stock Center	14028-0681.05
*D. ananassae*	National *Drosophila* Species Stock Center	14024-0371.12
*D. persimilis*	National *Drosophila* Species Stock Center	14011-0111.01
*D. persimilis*	National *Drosophila* Species Stock Center	14011-0111.02
*D. persimilis*	National *Drosophila* Species Stock Center	14011-0111.17
*D. persimilis*	National *Drosophila* Species Stock Center	14011-0111.46
*D. pseudoobscura*	National *Drosophila* Species Stock Center	14011-0121.148
*D. willistoni*	National *Drosophila* Species Stock Center	14030-0811.00
*D. mojavensis*	National *Drosophila* Species Stock Center	15081-1352.22
*D. virilis*	National *Drosophila* Species Stock Center	15010-1051.00
*D. san BDPG4U-Gal4@2253 (mw)*	This study	N/A
*D. san 20E08-Gal4@2253 (mw)*	This study	N/A
*D. san Or67d-Gal4 (ie1-mCherry)*	This study	N/A
*D. san 71G01-Gal4DBD@2251 (mw)*	This study	N/A
*D. san dsx-p65AD (MHC-DsRed)*	This study	N/A
*D. san UAS-nlsTdT@2253 (mw)*	Yun Ding	https://doi.org/10.1101/2025.05.06.652433
*D. san UAS-GCaMP7s-2A-tdT@2283 (mw)* (introgressed 10 generations from *D. yak*)	Hiroshi Shiozaki, David Stern	N/A
*D. san UAS-CsChrimson-tdT@2252 (mw)*	Yun Ding, David Stern	N/A
*D. san UAS-CsChrimson-tdT@2253 (mw)*	Yun Ding, David Stern	N/A
*D. san UAS-EGFP-Kir2.1@2174 (mw)*	Yun Ding, David Stern	https://doi.org/10.1016/j.cub.2019.02.019

Recombinant DNA		

pBPGUw	Addgene	Cat#17575 (Addgene_17575)
pBPZpGAL4DBDUw	Addgene	Cat#26233 (Addgene_26233)
pBPp65ADZpUw	Addgene	Cat#26234 (Addgene_26234)
pJAT32	Addgene	Cat#204297 (Addgene_204297)

Software and algorithms		

Geneious Prime	https://www.geneious.com/	RRID: SCR_010519
Caltech FlyTracker	https://kristinbranson.github.io/FlyTracker/	RRID: SCR_027431
JAABA	https://jaaba.sourceforge.net/	RRID: SCR_027430
Tempo	https://github.com/JaneliaSciComp/tempo	RRID: SCR_027432
Fiji	https://fiji.sc/	RRID: SCR_002285
Jupyter Notebook	https://jupyter.org/	RRID: SCR_018315
MATLAB	https://www.mathworks.com/products/matlab.html	RRID: SCR_001622
R	http://www.r-project.org/	RRID: SCR_001905
RStudio	https://posit.co/	RRID: SCR_000432
Illustrator 2025	http://www.adobe.com/products/illustrator.html	RRID: SCR_010279

## Highlights

- Male-directed courtship is common in *D. santomea* despite working sex recognition
- *D. santomea* males produce unusually low levels of the anti-aphrodisiac pheromone cVA
- *D. santomea* females show sexual aversion to cVA likely to avoid interspecies hybridization
- Similar social and pheromonal evolution has occurred convergently in *D. persimilis*
