# Supplementary material for: Evolutionary basis of male same-sex sexual behavior by multiple pheromone switches in Drosophila
Source: Curr Biol. Author manuscript; Available in PMC 2026 Apr 27. (PMC13120855; doi:10.1016/j.cub.2026.02.046)
Supplement: 1 [file NIHMS2152147-supplement-1.pdf]

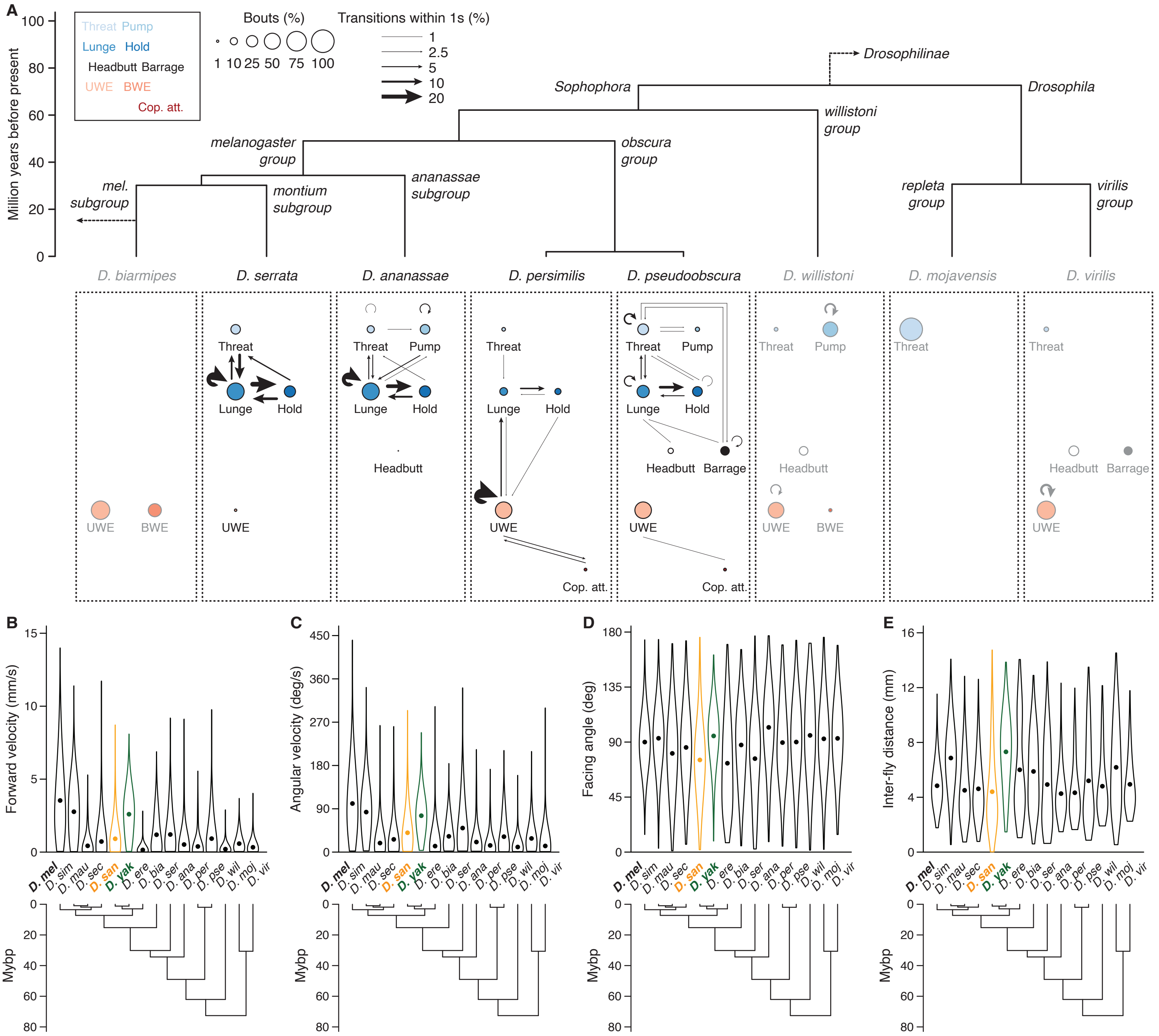

A
Million years before present
Threat Pump
Lunge Hold
Headbutt Barrage
UWE BWE
Cop. att.
Bouts (%)
1 10 25 50 75 100
Transitions within 1s (%)
1
2.5
5
10
20
Drosophilinae
Sophophora
Drosophila
willistoni group
melanogaster group
obscura group
mel. subgroup
montium subgroup
ananassae subgroup
repleta group
virilis group
D. biarmipes
D. serrata
D. ananassae
D. persimilis
D. pseudoobscura
D. willistoni
D. mojavensis
D. virilis
B
Forward velocity (mm/s)
C
Angular velocity (deg/s)
D
Facing angle (deg)
E
Inter-fly distance (mm)
D. mel D. sim D. mau D. sec D. san D. yak D. ere D. bia D. ser D. ana D. per D. pse D. wil D. moj D. vir
Mybp

Figure S1. Additional behavior and locomotor analyses of *Drosophila* intermale social interactions. Related to Figure 1.

(A) Ethograms from 20 min interactions depicting aggressive and courtship actions scored in three representative conspecific male-male pairs per species across various *Sophophora* and *Drosophila* species. Nodes are bout counts of a given action type, with sizes representing frequency normalized to summed bouts scored across all actions (expert annotation). Edges indicate action transitions, with weights representing the fraction of bouts of a given action (arrow origin) for which a second action (arrow destination) occurred within 1 s. Nine total actions in each ethogram are arranged in five rows: first, two aggressive threat actions (threat, pump)[S1]; second, two aggressive contact-mediated actions (lunge, hold)[S2]; third, two additional contact-mediated aggressive actions (headbutt, barrage)[S3]; fourth, two courtship actions utilizing the wings and typically directed toward females (UWE, BWE)[S4]; fifth, copulation attempt. Nodes indicating aggressive actions are filled with blue shades and courtship actions with reds, except for headbutt (white) and barrage (black). Species arrangement and divergence times according to compilation of available phylogenies[S5–S7]. Gray coloration indicates that very few social actions of any kind were observed. Abbreviations: UWE, unilateral wing extension; BWE, bilateral wing extension; Cop. att., copulation attempt. “Barrage” was likely described previously in *D. pseudoobscura*[S8] but is formally named here.

(B-E) Locomotor summaries for all 15 species included in the behavioral screen. Per-frame calculations of each fly’s forward velocity (B), angular velocity (C), facing angle relative to the partner (D), and inter-fly distance (E) are derived from automated tracking of both flies[S9] in all recorded male-male pairs. Distributions shown as violin plots with dotted means. *D. santomea,* orange; *D. yakuba*, green.

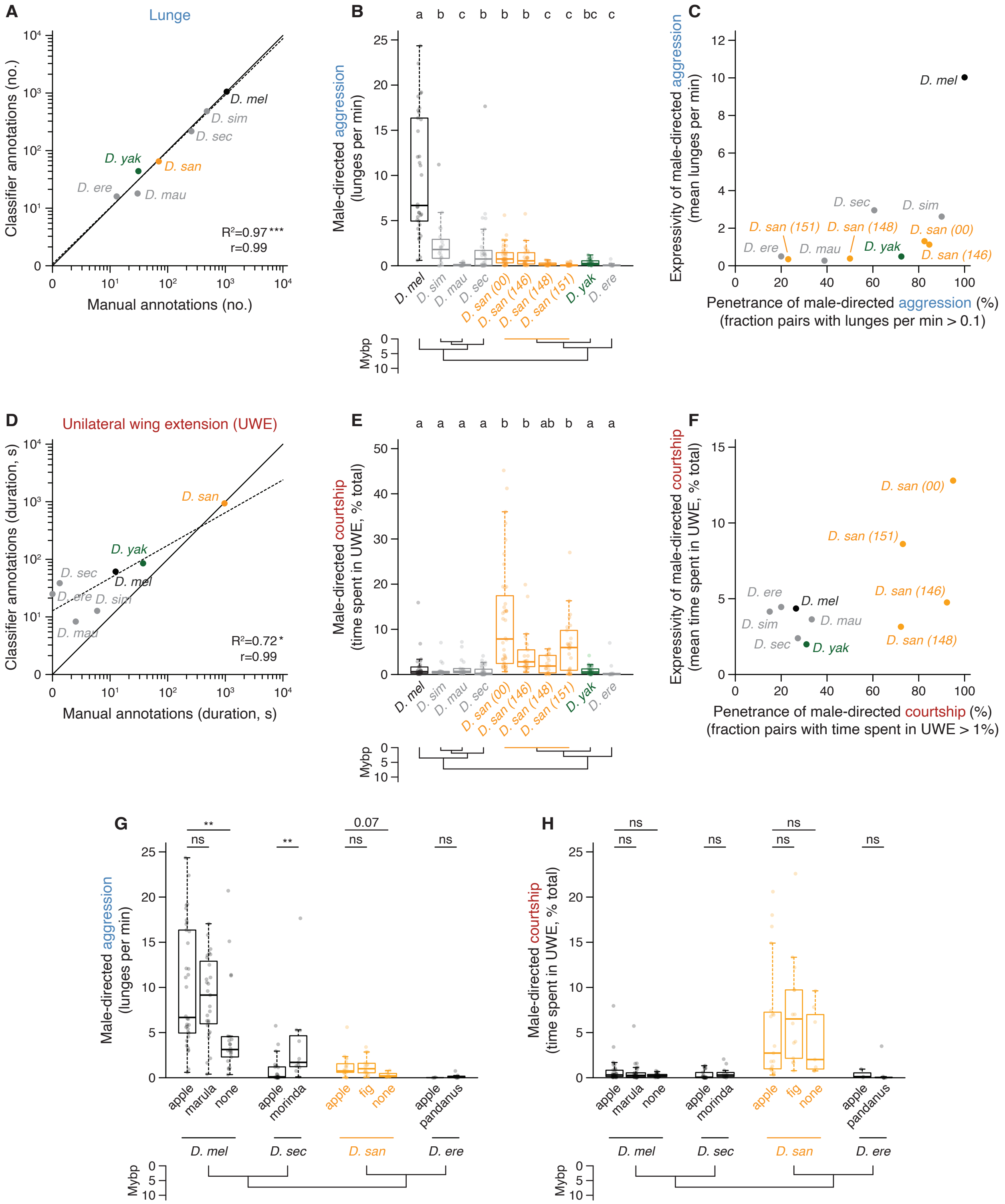

A
Lunge
Classifier annotations (no.)
Manual annotations (no.)
D. mel
D. sim
D. sec
D. yak
D. san
D. ere
D. mau
R^2=0.97***
r=0.99
B
a b c b b b c c bc c
Male-directed aggression (lunges per min)
D. mel
D. sim
D. mau
D. sec
D. san (00)
D. san (146)
D. san (148)
D. san (151)
D. yak
D. ere
Mybp
C
Expressivity of male-directed aggression (mean lunges per min)
Penetrance of male-directed aggression (%) (fraction pairs with lunges per min > 0.1)
D. mel
D. sec
D. sim
D. san (151)
D. san (148)
D. san (00)
D. ere
D. mau
D. yak
D. san (146)
D
Unilateral wing extension (UWE)
Classifier annotations (duration, s)
Manual annotations (duration, s)
D. san
D. yak
D. sec
D. mel
D. ere
D. sim
D. mau
R^2=0.72*
r=0.99
E
a a a a b b ab b a a
Male-directed courtship (time spent in UWE, % total)
F
Expressivity of male-directed courtship (mean time spent in UWE, % total)
Penetrance of male-directed courtship (%) (fraction pairs with time spent in UWE > 1%)
D. san (00)
D. san (151)
D. san (146)
D. san (148)
G
**
ns
**
0.07
ns
ns
Male-directed aggression (lunges per min)
apple
marula
none
morinda
fig
pandanus
D. mel
D. sec
D. san
D. ere
Mybp
H
ns
ns
ns
ns
ns
ns
Male-directed courtship (time spent in UWE, % total)

Figure S2. Male-directed courtship is a consistent trait in *D. santomea* across individuals, strains, and food conditions. Related to Figure 1.

(A,D) Automated behavior classifier performance for lunge (A) and unilateral wing extension (UWE, D) as indicators of male aggression and courtship, respectively. Three 20 min representative recordings of male-male pairs for each species were manually scored for both actions (expert annotation, same pairs as Figure 1A) and exposed to automated behavior classifiers trained previously for *D. melanogaster*[S10], implemented in JAABA[S11]. Manual (x-axis) vs. classifier-generated annotations (y-axis) are compared in $\log_{10}$ scale, using either the number of bouts (lunge) or their cumulative time duration (UWE). Solid lines, diagonals (perfect agreement); dashed lines, linear fits. Adjusted R^2, significance by F-tests for fits, and Pearson correlations (R) indicated at bottom right. *D. melanogaster*, black; *D. santomea,* orange; *D. yakuba*, green; *D. simulans*, *D. mauritiana*, *D. sechellia*, *D. erecta*, gray. Note near-perfect overlap with the diagonal for lunge and strong correlation for UWE despite classifier-generated false positives in cases with very few manual annotations (all subgroup species except *D. santomea* show little male-male courtship).

(B,E) Aggression (lunge, B) and courtship (UWE, E) measurements from automated classifiers in all recorded male-male pairs for *melanogaster* subgroup species and three additional *D. santomea* strains. *D. santomea* 00 (also called STO.4) is the strain included in the behavioral screen and used throughout as "wildtype" (National Drosophila Species Stock Center 14021.0271.00). Strains 146 (STO-CAGO 1482), 148 (STO.7), and 151 (STO.6) also derive from São Tomé from females collected between 1100 and 1500 m elevation[S12]. Boxplots show full distribution range within whiskers (excluding statistically identified outliers) and second and third quartiles within boxes, with medians in bold. Individual data points (including outliers) overlaid as gray dots. Outliers are retained in all summary metrics and statistical comparisons calculated here and throughout. Lettered statistical groupings assigned by post-hoc Dunn's tests following significant Kruskal-Wallis. Note low aggression and high courtship conserved in *D. santomea* strains (orange), low aggression and low courtship in the sibling species *D. yakuba* (green), and high aggression and low courtship in *D. melanogaster* (black).

(C,F) Summaries of penetrance (x-axes) and expressivity (y-axes) for aggression (C) and courtship (F) across subgroup species and *D. santomea* strains. Metrics derived from data in (B,E).

(G,H) Aggression (lunge, G) and courtship (UWE, H) in *D. melanogaster*, *D. sechellia*, *D. santomea* (orange), and *D. erecta* male-male pairs competing over the standard apple juice substrate and other fruits selected based on reported specializations[S13]. Significance for each fruit/species combination by pairwise Mann-Whitney *U* tests to apple. Removing the food source significantly reduces aggression in *D. melanogaster*[S14]. *Morinda* (also called noni) significantly increases aggression but not courtship among *D. sechellia* males. *D. santomea* aggression and courtship depend only weakly if at all on food presence and

source. *Morinda* and fig juices obtained commercially, *Marula* (*Sclerocarya birrea*) and *Pandanus* (*Pandanus furcatus*) obtained from botanical gardens (see Key Resources Table).

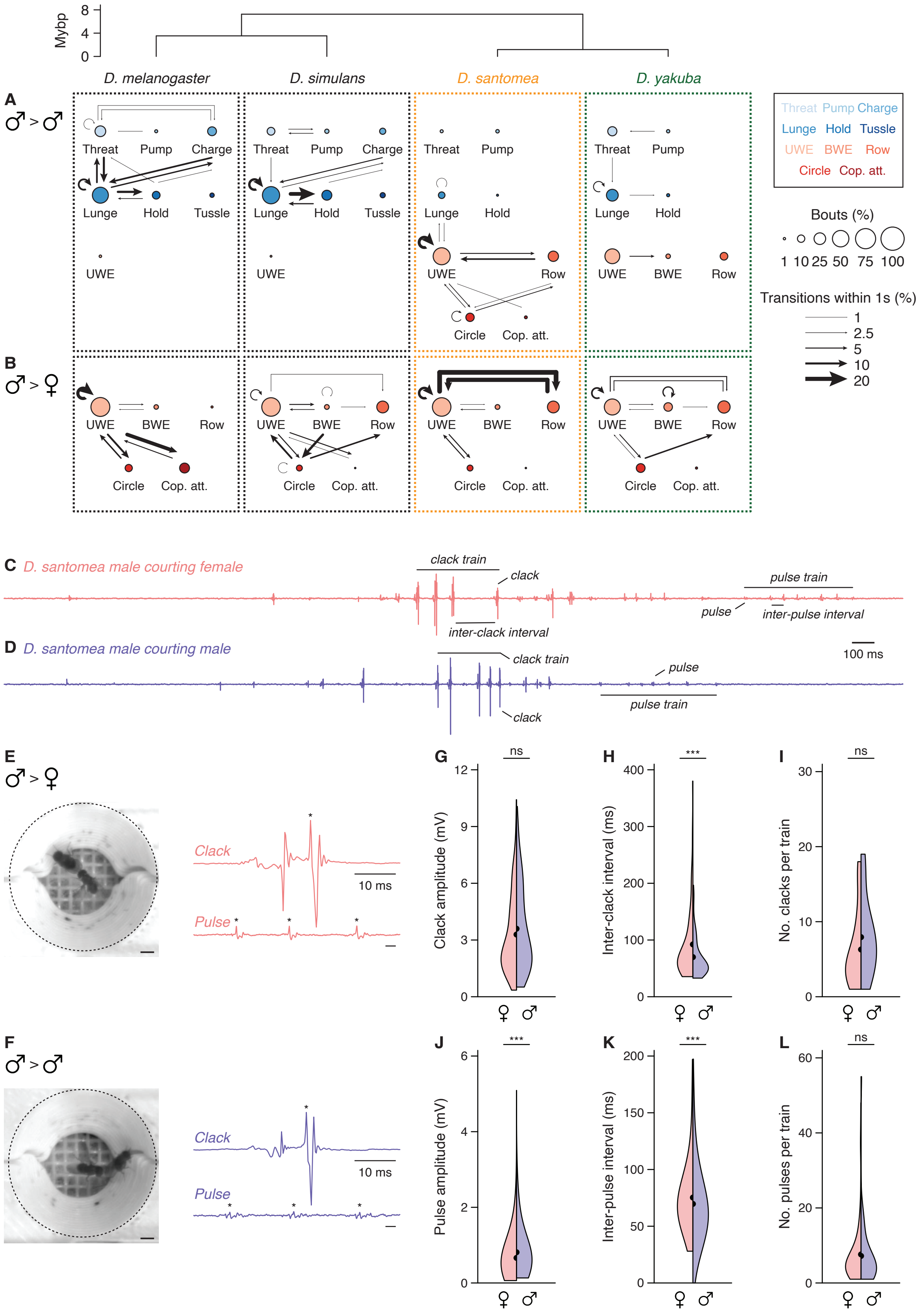

Mybp
D. melanogaster
D. simulans
D. santomea
D. yakuba
A
♂ > ♂
Threat Pump Charge
Lunge Hold Tussle
UWE BWE Row
Circle Cop. att.
Bouts (%)
1 10 25 50 75 100
Transitions within 1s (%)
1
2.5
5
10
20
B
♂ > ♀
C D. santomea male courting female
clack train
clack
pulse train
pulse
inter-pulse interval
inter-clack interval
D D. santomea male courting male
100 ms
E
F
Clack
Pulse
10 ms
G
Clack amplitude (mV)
H
Inter-clack interval (ms)
I
No. clacks per train
J
Pulse amplitude (mV)
K
Inter-pulse interval (ms)
L
No. pulses per train
ns
***

Figure S3. Visual and acoustic similarity of male- and female-directed courtship by *D. santomea*. Related to Figure 1.

(A,B) Ethograms representing social behaviors by *D. melanogaster*, *D. simulans*, *D. santomea*, and *D. yakuba* males in conspecific male-male (A) and male-female (B) pairings. Ethograms represent expert annotation from three male-male and three male-female pairs per species, with male tester flies prepared identically by single-housing and interactions taking place under identical conditions. Male-directed ethograms are reproduced from Figure 1A for visual comparison to female-directed counterparts. Note nearly exclusive male-directed aggressive actions (blue nodes) by *D. melanogaster* and *D. simulans* males but increased abundance of male-directed courtship actions (red nodes) by *D. yakuba* and, especially, *D. santomea* males, matching the actions taken when paired with females. Though not apparent here due to normalization by bout number, total *D. yakuba* male-directed actions are many fewer on average than in *D. santomea* (see Figure 1B).

(C,D) Representative courtship song traces by *D. santomea* males toward a female (pink, C) or male conspecific target fly (purple, D). Key acoustic features are indicated including pulse, pulse train, inter-pulse interval (IPI), clack, clack train, and inter-clack interval (ICI)[S15]. Traces reproduced and enlarged from Figure 1I,J.

(E,F) Video stills of song recording chambers[S16] containing *D. santomea* males courting either a female (E) or male (F). Isolated waveforms of single clacks and pulse trios shown below, with asterisks indicating event peaks. Scale bars, 1 mm.

(G-L) Quantitative acoustic analyses of female- (pink) and male-directed *D. santomea* courtship songs (purple). Clacks (G-I) and pulses (J-L) were identified by expert annotation from three recordings with each sex (clack, 32-103 events per recording; pulse, 140-346 events per recording). Data shown as "hemiviolin" plots in which female- and male-directed kernel densities are vertically mirrored. Distribution means are semicircles on the appropriate side. Note general similarity between female- and male-directed song features (significant differences based on Mann-Whitney *U* tests despite small difference magnitudes are often because so many data points make up the underlying distributions). Analyses of absolute event abundances were omitted since annotations captured only a fraction of events in each recording.

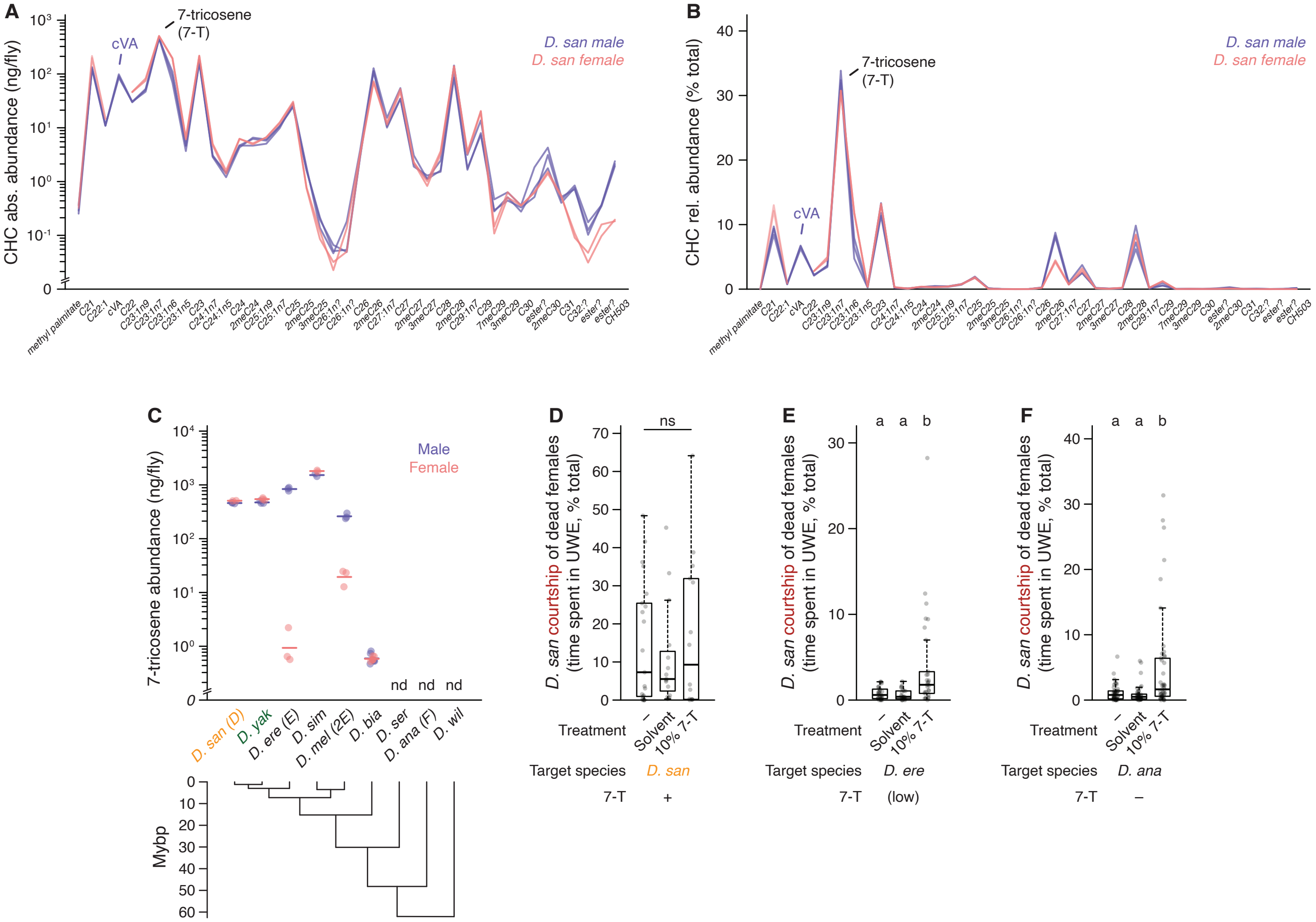

A
7-tricosene
(7-T)
cVA
CHC abs. abundance (ng/fly)
D. san male
D. san female
B
CHC rel. abundance (% total)
7-tricosene
(7-T)
cVA
D. san male
D. san female
C
7-tricosene abundance (ng/fly)
Male
Female
nd
nd
nd
D. san (D)
D. yak
D. ere (E)
D. sim
D. mel (2E)
D. bia
D. ser
D. ana (F)
D. wil
Mybp
D
D. san courtship of dead females (time spent in UWE, % total)
ns
Treatment
–
Solvent
10% 7-T
Target species
D. san
7-T
+
E
D. san courtship of dead females (time spent in UWE, % total)
a
a
b
Treatment
–
Solvent
10% 7-T
Target species
D. ere
7-T
(low)
F
D. san courtship of dead females (time spent in UWE, % total)
a
a
b
Treatment
–
Solvent
10% 7-T
Target species
D. ana
7-T
–

Figure S4. 7-tricosene (7-T) is sexually monomorphic in *D. santomea* and promotes courtship by *D. santomea* males when added onto dead heterospecific females. Related to Figure 2.

(A,B) Absolute (A) and relative abundances (B) of 41 compounds identifiable as peaks in *D. santomea* male (purple) and female hexane extracts (pink). Individual measurements are made on 10 pooled, age-matched adult flies (3 male, 2 female replicates). Calculation of absolute abundance uses known concentration of the internal standard (octadecane/C18, not shown) and normalizes for the number of flies included in the extract. Relative abundances normalize to the summed absolute abundance of all compounds excluding the standard. Male-specific (Z)-11-octadecenyl acetate (cVA, purple) and monomorphic (Z)-7-tricosene (7-T, black) are indicated. Question marks in compound names indicate uncertainty in chemical assignment. See Table S1 for retention times and diagnostic ions used to identify and assign CHCs.

(C) Absolute 7-T abundances on males (purple) and females (pink) of multiple *Drosophila* species measured by GC-MS on hexane extracts from 10 pooled, age-matched adult flies (2-8 replicates each). Monomorphic species (*D. santomea, D. yakuba*, *D. simulans*) show similar 7-T abundance between sexes while dimorphic species show large differences (*D. erecta*, *D. melanogaster*). nd, not detected in either sex (*D. serrata*, *D. ananassae*, *D. willistoni*). Species with females selected for 7-T addition experiments indicated by panel in parentheses.

(D-F) Fraction of time *D. santomea* males spend courting conspecific (D) or heterospecific dead females (E,F) perfumed with 10% 7-T (20 µg/fly) or solvent control (hexane). Heterospecific female targets selected for having either low (*D. erecta*) or undetectable (*D. ananassae*) endogenous 7-T levels (from GC-MS in C). *D. santomea* female targets as positive controls. 7-T addition elicits courtship from *D. santomea* males in both heterospecific cases. Significance by Dunn's tests. High quantities of 7-T were used since dead target flies are unable to provide moving visual cues that normally promote male courtship[S17–S20].

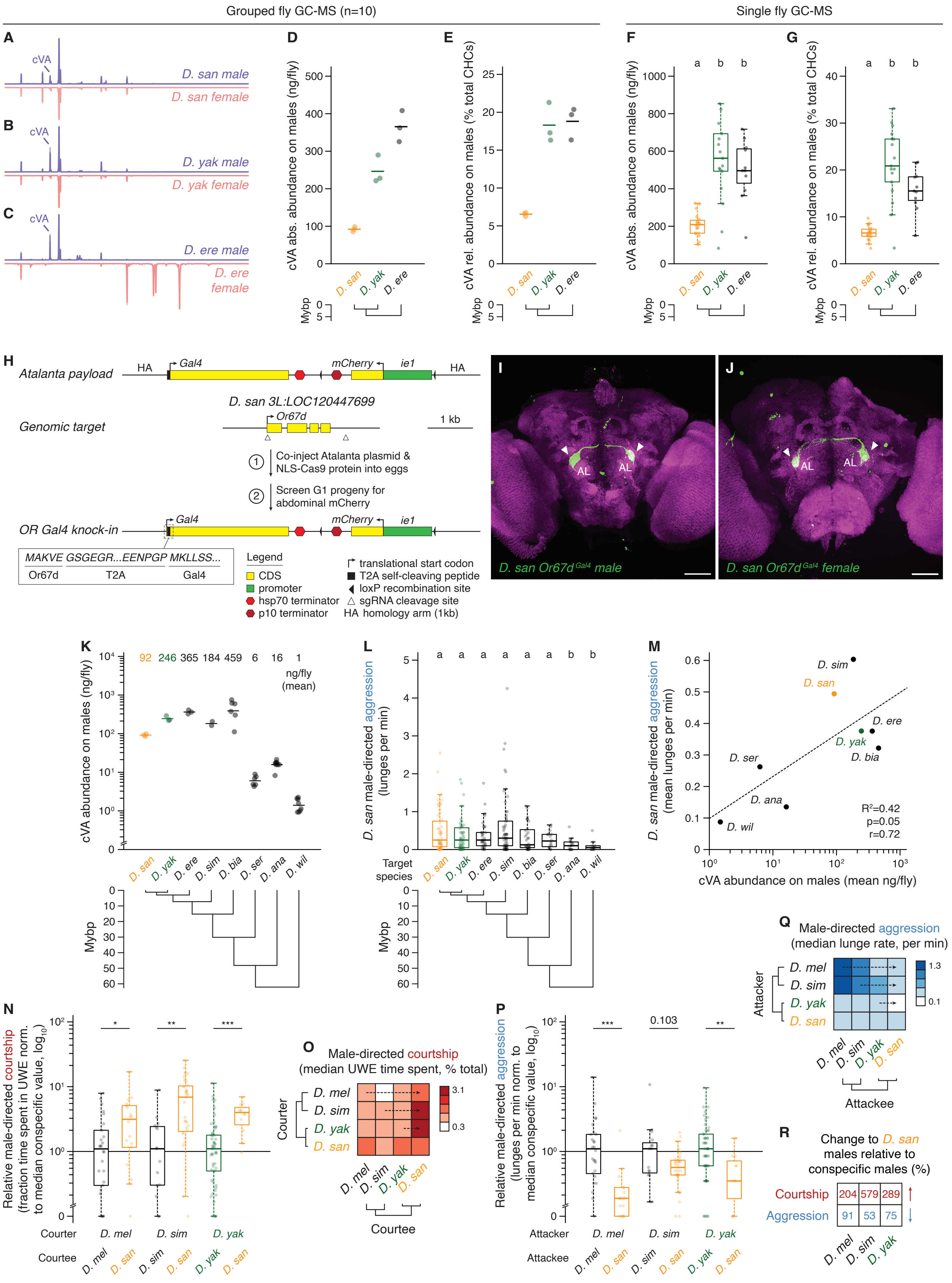

Grouped fly GC-MS (n=10)
Single fly GC-MS
A
cVA
D. san male
D. san female
B
cVA
D. yak male
D. yak female
C
cVA
D. ere male
D. ere female
D
cVA abs. abundance on males (ng/fly)
D. san
D. yak
D. ere
Mybp
E
cVA rel. abundance on males (% total CHCs)
F
cVA abs. abundance on males (ng/fly)
a b b
G
cVA rel. abundance on males (% total CHCs)
a b b
H
Atalanta payload
HA
Gal4
mCherry
ie1
D. san 3L:LOC120447699
Genomic target
Or67d
1 kb
Co-inject Atalanta plasmid & NLS-Cas9 protein into eggs
Screen G1 progeny for abdominal mCherry
OR Gal4 knock-in
MAKVE GSGEGR...EENPGP MKLLSS...
Or67d
T2A
Gal4
Legend
CDS
promoter
hsp70 terminator
p10 terminator
translational start codon
T2A self-cleaving peptide
loxP recombination site
sgRNA cleavage site
HA homology arm (1kb)
I
AL
D. san Or67d^Gal4 male
J
AL
D. san Or67d^Gal4 female
K
cVA abundance on males (ng/fly)
92 246 365 184 459 6 16 1
ng/fly (mean)
D. san
D. yak
D. ere
D. sim
D. bia
D. ser
D. ana
D. wil
Mybp
L
D. san male-directed aggression (lunges per min)
a a a a a a b b
Target species
M
D. san male-directed aggression (mean lunges per min)
cVA abundance on males (mean ng/fly)
D. sim
D. san
D. ere
D. yak
D. bia
D. ser
D. ana
D. wil
R²=0.42
p=0.05
r=0.72
N
Relative male-directed courtship (fraction time spent in UWE norm. to median conspecific value, log10)
Courter
Courtee
D. mel
D. sim
D. yak
D. san
O
Male-directed courtship (median UWE time spent, % total)
Courter
Courtee
3.1
0.3
P
Relative male-directed aggression (lunges per min norm. to median conspecific value, log10)
0.103
Attacker
Attackee
Q
Male-directed aggression (median lunge rate, per min)
Attacker
Attackee
1.3
0.1
R
Change to D. san males relative to conspecific males (%)
Courtship 204 579 289
Aggression 91 53 75

Figure S5. GC-MS confirmation of reduced cVA on *D. santomea* males, CRISPR-Cas9 HDR knock-in strategy used to generate *D. santomea Or67d*^{Gal4}, ineffectiveness of naturally varying cVA levels to promote aggression by *D. santomea* males, and social behavior changes elicited by *D. santomea* males from other *melanogaster* subgroup males. Related to Figure 3.

(A-C) Mirrored gas chromatograms representing male (purple, top) and female (pink, bottom) cuticular hydrocarbon (CHC) profiles of *D. santomea* (A), *D. yakuba* (B), and *D. erecta* (C). Individual measurements made on hexane extracts from 10 pooled, age-matched adult flies (2-3 replicates each). Peaks representing male-specific (Z)-11-octadecenyl acetate (cVA) indicated. *D. santomea* chromatograms reproduced from Figure 2A.

(D,E) Absolute (D) and relative abundances (E) of cVA on *D. santomea* (orange), *D. yakuba* (green), and *D. erecta* males from peaks in (A-C). *D. santomea* males show 63% and 75% reduced cVA comparing absolute abundance means, or 64% and 65% reductions comparing relative abundance means, to *D. yakuba* and *D. erecta*, respectively.

(F,G) Absolute (F) and relative abundances (G) of cVA on *D. santomea* (orange), *D. yakuba* (green), and *D. erecta* males from GC-MS measurements made and analyzed identically to (A-E) but using single flies as the input for hexane extracts (11-20 replicates each). Similarly to grouped flies, *D. santomea* males show 63% and 58% reduced cVA comparing absolute abundance medians, or 69% and 58% reductions comparing relative abundance medians, to *D. yakuba* and *D. erecta*, respectively. Significance by Dunn's tests. Note also little variation in cVA abundance among *D. santomea* males.

(H) Schematic diagrams of the Gal4 transgene and selection marker assembled into an Atalanta vector[S21] (pJAT32, addgene #204297) and *Or67d* genomic target locus shown with upstream and downstream sgRNA cleavage sites. Transgene (above) contains two cassettes: *T2A-Gal4-stop(hsp70)* oriented natively to the genomic target, and downstream *ie1-mCherry-stop(p10)* fluorescent integration marker[S22] with inverted orientation. 1 kb homology arms flank both ends. *Or67d* genomic locus (below) is targeted using sgRNAs directing double-strand breaks to 15 bp downstream of the translational start codon and 348 bp downstream of the stop, resulting in removal of 1152 of 1167 protein-coding base pairs (99%). Co-injection of the assembled Atalanta vector and nls-Cas9-nls protein into wildtype *D. santomea* eggs yields mosaic *ie1-mCherry* expression in G0 progeny carrying *T2A-Gal4* inserted in-frame with the 5 residual N-terminal residues of *Or67d*. G1 progeny resulting from G0 crosses to wildtype are re-screened for abdominal *ie1-mCherry* expression to ensure germline transmission. In this strategy the spatiotemporal pattern and magnitude of Gal4 expression are controlled entirely by *Or67d* endogenous genomic regulation.

(I,J) Photomicrographs showing labeling patterns of *Or67d*^{Gal4} in the whole brain of a *D. santomea* male (I, same fly from which a close-up of the antennal lobes (ALs) is shown in Figure 3F) and female (J).

Immunostaining for Gal4-dependent cytoplasmic tdTomato pseudocolored green with Bruchpilot synaptic counterstain (using nc82 monoclonal antibody) in magenta. Uniglomerular labeling pattern in each AL (arrowheads) matches the expected size and position of cVA-sensitive DA1 targeted by Or67d OSNs[S23,24]. Bifurcating OSN commissures (axon bundles) from the antennae leading into ALs can also be seen. Scale bars, 50 μm.

Absolute cVA abundance on *Drosophila* males measured by GC-MS on hexane extracts from 10 pooled, age-matched adult flies (2-8 replicates each). *D. santomea* (orange), *D. yakuba* (green), and *D. erecta* measurements reproduced from (D) for visual comparison to additional species. Means indicated above and phylogeny below.

Spontaneous aggression exhibited by *D. santomea* males toward conspecific (orange) or heterospecific males (*D. yakuba* green, all others black) in single-housed pairs during 20 min interactions. Statistical groupings by Dunn's tests shown above and phylogeny below. Note consistently low or even further decreased attack levels toward males of all species.

Correlation between cVA abundance measured by GC-MS (K) and aggression elicited from *D. santomea* males (L). Dashed line, linear fit. Adjusted R^2, significance by F-test for fit, and Pearson correlation (R) indicated at bottom right. Note marginally significant correlation driven mostly by low attack rates toward distantly related species (*D. serrata*, *D. ananassae*, *D. willistoni*) with many other pheromone changes in addition to lowered cVA. Three of four species with higher cVA abundance than *D. santomea* elicit less attack than observed among *D. santomea* males (*D. simulans* is the exception but the slight increase is non-significant).

Relative fraction of time *D. melanogaster*, *D. simulans*, and *D. yakuba* (green) tester males court conspecific males (first distribution of each pair) vs. *D. santomea* males (second distribution, orange). All measurements within each species are normalized to the conspecific median and $\log_{10}$ transformed. Males of all three *melanogaster* subgroup species show significantly increased courtship toward *D. santomea* males by Mann-Whitney *U* tests, including *D. melanogaster* where 7-T (abundant on *D. santomea* males) is male-specific and sexually aversive[S25–28].

Heatmap showing the median fraction of time spent courting by males in conspecific (diagonal) and heterospecific male-male pairs (off-diagonal) among the four *melanogaster* subgroup species. Rows ("courters") are tester males whose courtship is measured (UWE). Columns ("courtees") are males targeted by courtship. Color code shown on right. Data derived from (N) and additional pairings. Horizontal dashed arrows identify relevant comparisons between courtship directed to conspecific males vs. directed to *D. santomea* males for visual aid.

Similar boxplots to (N) but showing paired relative distributions for aggression within and between males of the three *melanogaster* subgroup species. Note decreases in aggression by males of all three

species toward *D. santomea* males (significant for *D. melanogaster* and *D. yakuba* by Mann-Whitney *U* tests).

(Q) Similar heatmap to (O) showing median aggression rates for male “attackers” (rows) paired with various conspecific and heterospecific “attackees” (columns). Data derived from (P) and additional pairings. Note consistently decreased aggression toward *D. santomea* males.

(R) Summarized changes in courtship (first row) and aggression rates (second row) by *melanogaster* subgroup males toward *D. santomea* compared to conspecific rates for each, derived from data in (N-Q). Percent increases in first row (courtship, red) and percent decreases in second (aggression, blue). *D. santomea* males attract courtship and deter attack.

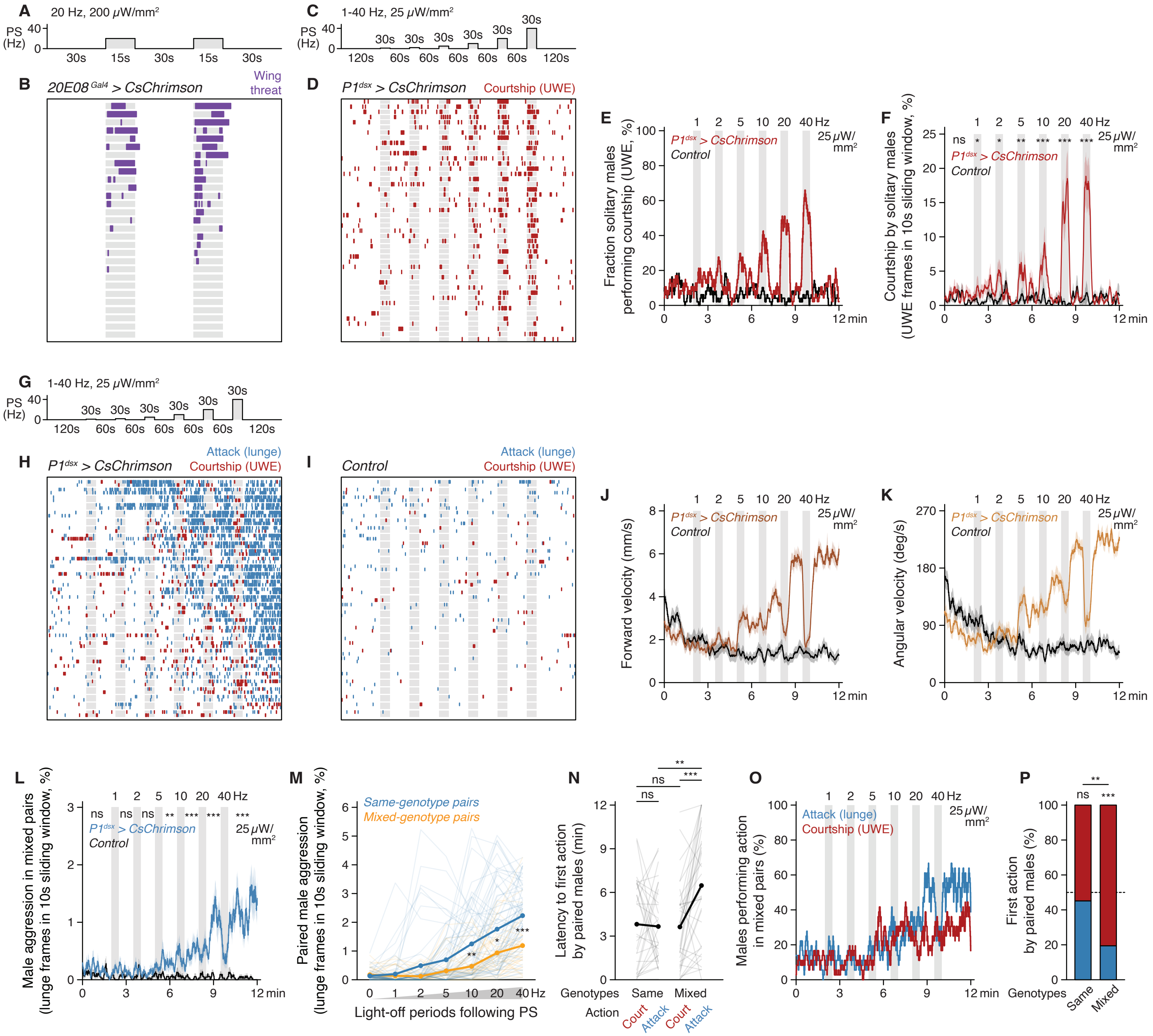

A
20 Hz, 200 μW/mm²
PS (Hz)
30s 15s 30s 15s 30s
B
20E08 Gal4 > CsChrimson
Wing threat
C
1-40 Hz, 25 μW/mm²
D
P1dsx > CsChrimson
Courtship (UWE)
E
Fraction solitary males performing courtship (UWE, %)
F
Courtship by solitary males (UWE frames in 10s sliding window, %)
Control
G
H
Attack (lunge)
I
J
Forward velocity (mm/s)
K
Angular velocity (deg/s)
L
Male aggression in mixed pairs (lunge frames in 10s sliding window, %)
M
Same-genotype pairs
Mixed-genotype pairs
Paired male aggression (lunge frames in 10s sliding window, %)
Light-off periods following PS
N
Latency to first action by paired males (min)
Genotypes
Same
Mixed
Action
Court
Attack
O
Males performing action in mixed pairs (%)
P
First action by paired males (%)

Figure S6. Behavioral and locomotor effects evoked by photoactivation of AIP and P1^{dsx} neurons in *D. santomea* males. Related to Figure 4.

(A) Optogenetic photoactivation scheme for solitary *D. santomea* males carrying *20E08^{Gal4}* and Gal4-dependent CsChrimson. Group-housed *20E08^{Gal4}* tester males or genetic controls (homozygous parental lines) are exposed to two 15 s photostimulation (PS) blocks with a 30 s baseline and interblock interval (IBI).

(B) Manually scored wing threat rasters (purple) for solitary *20E08^{Gal4}* males during the 2 min photoactivation trial. Note threat induction time-locked to PS in most flies. Solitary genetic controls showed no threat.

(C) Optogenetic photoactivation scheme for solitary group-housed *D. santomea* males carrying *P1^{dsx}* split-Gal4 (*71G01^{DBD};dsx^{AD}*) and Gal4-dependent CsChrimson. *P1^{dsx}* tester males or a genetic control (males possessing *71G01^{DBD}* and CsChrimson but missing the *dsx^{AD}* hemidriver) are exposed to six 30 s PS blocks with fixed intensity and monotonically increasing frequency as indicated, with a 2 min baseline and 1 min IBIs.

(D) Courtship rasters (UWE, red) for *P1^{dsx}* tester males during the 12 min trial. Note early sporadic courtship bouts that time-lock to PS blocks and increase in penetrance starting at 5 and then 10 Hz. Solitary genetic controls showed no courtship.

(E,F) Penetrance (E) and expressivity (F) of courtship elicited by *P1^{dsx}* PS. Significant courtship expression during PS blocks compared to the genetic control in (F) by Mann-Whitney *U* tests.

(G) Optogenetic photoactivation scheme for same-genotype pairs of group-housed *D. santomea* males carrying *P1^{dsx}* split-Gal4 and Gal4-dependent CsChrimson. Identical trial structure and PS conditions as in (C).

(H,I) Behavior rasters for *P1^{dsx}* male pairs (H) and genetic controls (I, males missing *dsx^{AD}*) during the 12 min trial. Blue, attack (lunge); red, courtship (UWE). Note early induction of attack which increases in penetrance and expressivity during the trial. Attacks interrupted by time-locked courtship during later PS blocks (>5 Hz). Genetic controls show little courtship or attack.

(J,K) Locomotor dynamics in *P1^{dsx}* males (browns) and genetic controls (black). Per-frame calculations of each fly's forward velocity (J) and angular velocity (K) derived from automated tracking[9]. Data smoothed by averaging within a ten-second sliding window. Envelopes represent s.e.m. PS time-locked locomotor arrest as observed previously in *D. melanogaster*[S10].

(L) Expressivity and temporal dynamics of attack (lunge) elicited by PS in mixed-genotype pairs between a *P1^{dsx}* split-Gal4 tester male carrying Gal4-dependent CsChrimson (or a genetic control male missing *dsx^{AD}*) and group-housed wildtype *D. santomea* target male. Traces show the mean fraction of frames containing lunge in a 10 s sliding window with s.e.m. envelopes for *P1^{dsx}* (blue) and control (black). PS

block frequency, intensity, and timing indicated above for the 12 min trial (same structure as G). Note significant attack induction (Mann-Whitney *U* tests) in $P1^{dsx}$ starting after 5 Hz.

(M) Attack elicited during IBIs and after final PS in same-genotype $P1^{dsx}$ male pairs (blue, data from Figure 4I) vs. mixed-genotype pairs between a $P1^{dsx}$ tester and group-housed wildtype male (orange, from L). Period means connected with solid lines and data from individual flies shown as thin lines underneath. Note significant right shift in mixed pairs (Mann-Whitney *U* tests) indicating higher PS frequencies required to induce intense attack.

(N) Action latencies for courtship and attack exhibited by $P1^{dsx}$ tester males in same- and mixed-genotype pairs using identical PS trial structures. Data points derived from the same fly pair (or tester fly for mixed pairs) are connected as lines and distribution means shown as paired dots. Attack and courtship latencies are similar on average in same-genotype pairs whereas attack is significantly delayed in mixed pairs. Significance by paired or unpaired Mann-Whitney *U* tests (for within- or between-genotype group comparisons, respectively).

(O) Fraction of flies showing attack (lunge, blue) and courtship (UWE, red) in $P1^{dsx}$ mixed-genotype pairs.

(P) Relative frequencies of $P1^{dsx}$ same- and mixed-genotype pairs showing courtship (red) or attack (blue) first. Significance between distributions and between each distribution and a null probability of 50% (random chance) determined by binomial tests. Note increased courtship precedence in mixed pairs.

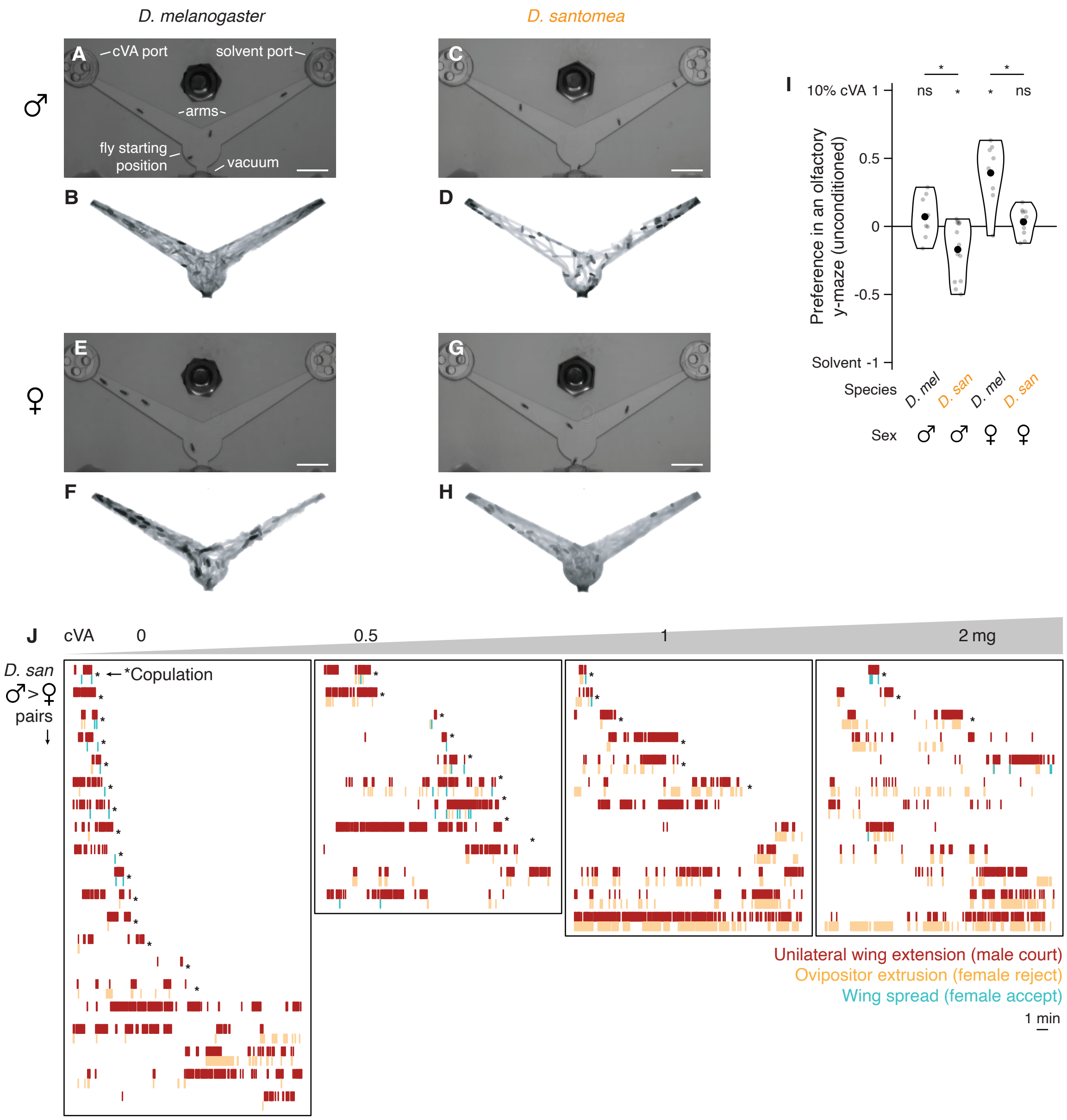
D. melanogaster
D. santomea
A
cVA port
solvent port
arms
fly starting position
vacuum
B
C
D
E
F
G
H
♂
♀
I
10% cVA 1
0.5
0
-0.5
Solvent -1
Preference in an olfactory y-maze (unconditioned)
ns
*
*
ns
Species
D. mel
D. san
D. mel
D. san
Sex
♂
♂
♀
♀
J
cVA
0
0.5
1
2 mg
D. san
♂>♀
pairs
*Copulation
Unilateral wing extension (male court)
Ovipositor extrusion (female reject)
Wing spread (female accept)
1 min

Figure S7. cVA is innately and sexually aversive to *D. santomea* females. Related to Figure 5.

(A) Video still showing the y-maze arena used for odor preference testing. Four group-housed *D. melanogaster* males are introduced into the central bowl at the bottom and odors (10% cVA in acetone or acetone control) into ports at the head of each arm. Vacuum pressure continuously pulls air down the arms through an outlet below the bowl. Flies can walk freely within the bowl and arms during 10 min trials and odors are replaced before each trial. Scale bar, 10 mm.

(B) Cumulative position trace representing movement of all four flies throughout the duration of the trial. Position density in each of multiple trials is used to calculate the odor preference index.

(C-H) Video stills and cumulative position traces as in (A,B) for *D. santomea* males (C,D), *D. melanogaster* females (E,F), and *D. santomea* females (G,H). Scale bars, 10 mm.

(I) Odor preference indices by species and sex. Index calculated as the difference in fly occupancy between the two arms normalized by the sum. Small gray dots represent single trials with 2-4 flies each (8-14 trials and 26-44 flies per species/sex). Distributions represented as violin plots shown overlaid with individual data points (small dots) and means (large dots). Note significantly reduced attraction (or increased aversion) to 10% cVA in both *D. santomea* males and females (orange) compared to *D. melanogaster* (black). Significance by Mann-Whitney *U* tests.

(J) Rasters of *D. santomea* male and female sexual behaviors during dyadic courtship interactions with increasing cVA on a nearby piece of filter paper. For each pair, male courtship (UWE, red) rasters are shown above and female rejection (ovipositor extrusion, orange) or acceptance (wing spreading, cyan) rasters below. Timing of copulations indicated with asterisks. cVA doses indicated at top. Note increased rejection and decreased copulation rates with 1 and 2 mg cVA.

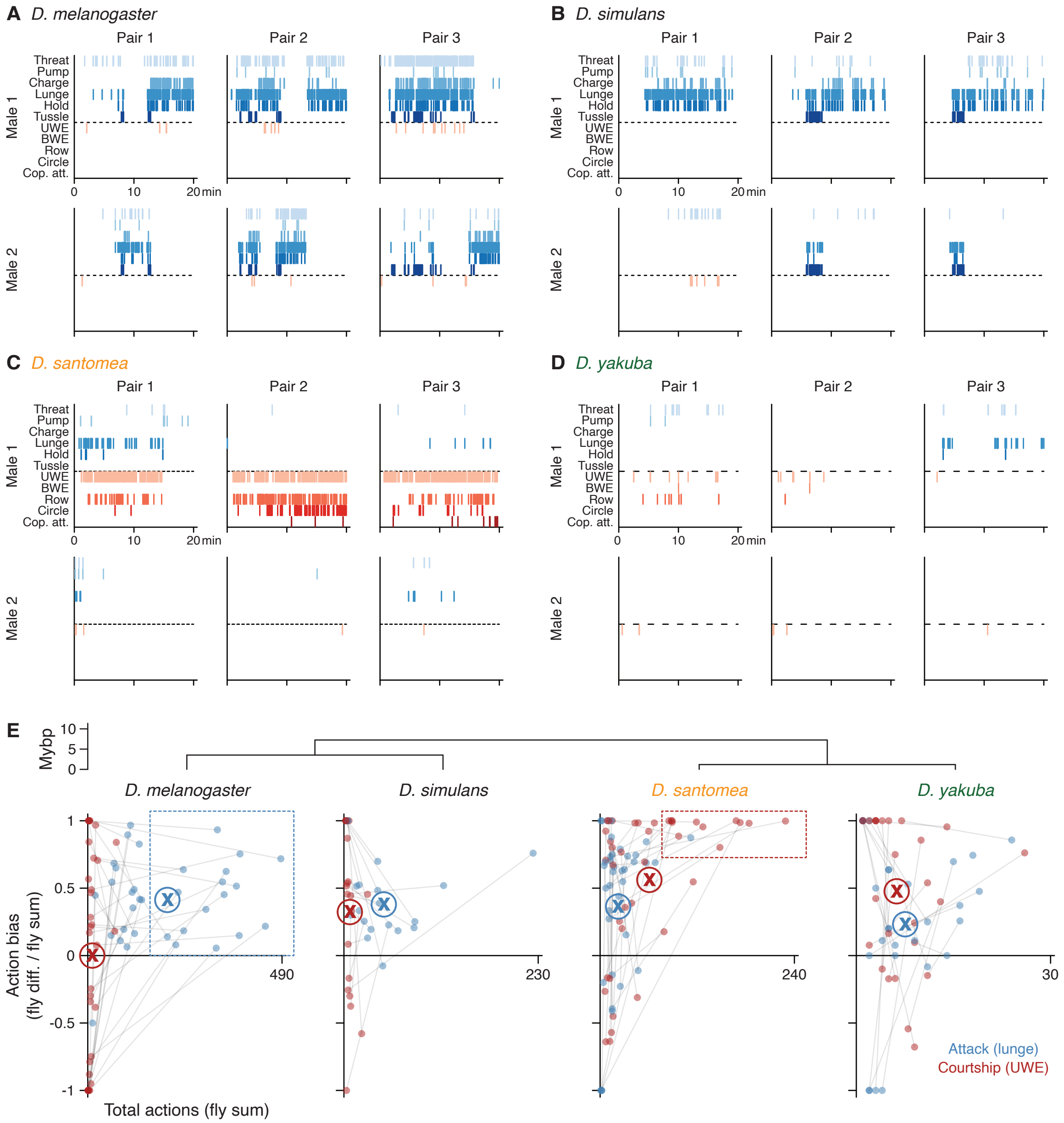
A D. melanogaster
B D. simulans
C D. santomea
D D. yakuba
Pair 1
Pair 2
Pair 3
Male 1
Male 2
Threat
Pump
Charge
Lunge
Hold
Tussle
UWE
BWE
Row
Circle
Cop. att.
0
10
20 min
E
Mybp
10
5
0
Action bias
(fly diff. / fly sum)
Total actions (fly sum)
490
230
240
30
1
0.5
0
-0.5
-1
Attack (lunge)
Courtship (UWE)

Figure S8. Aggression and courtship biases in intermale social interactions within the *melanogaster* subgroup. Related to Figure 6.

(A-D) Behavior rasters for three 20 min conspecific male-male pairs each of wildtype *D. melanogaster* (A), *D. simulans* (B), *D. santomea* (C), and *D. yakuba* (D) (same pairs as Figure 1A). Annotations of six aggressive interactions by manual scoring (blues) are shown above the horizontal dashed lines and five courtship actions (reds) beneath. Actions split by identity of the male to which they are attributed with the one exhibiting the greater total number of actions assigned as "Male 1." *D. melanogaster* and *D. simulans* males fight with some characteristic bias between flies, *D. yakuba* males are mostly passive but can either fight or court, and *D. santomea* males show frequent and elaborate courtship often with strong bias between flies.

(E) Spontaneous action biases observed between flies in male-male pairs. Male-directed courtship (red dots) and attack (blue dots) are plotted as a function of their bout abundance (x-axis) and bias indices (y-axis). Measurements from the same pair connected by thin lines. Cases where the same fly is dominant for both actions show connections confined to the sector above the x-axis. Circled "X" indicates mean value for the corresponding action by color. Boxed regions indicate pairs with high attack expression and wide range of inter-fly biases in *D. melanogaster* or high courtship expression and consistently strong bias in *D. santomea,* for visual aid.

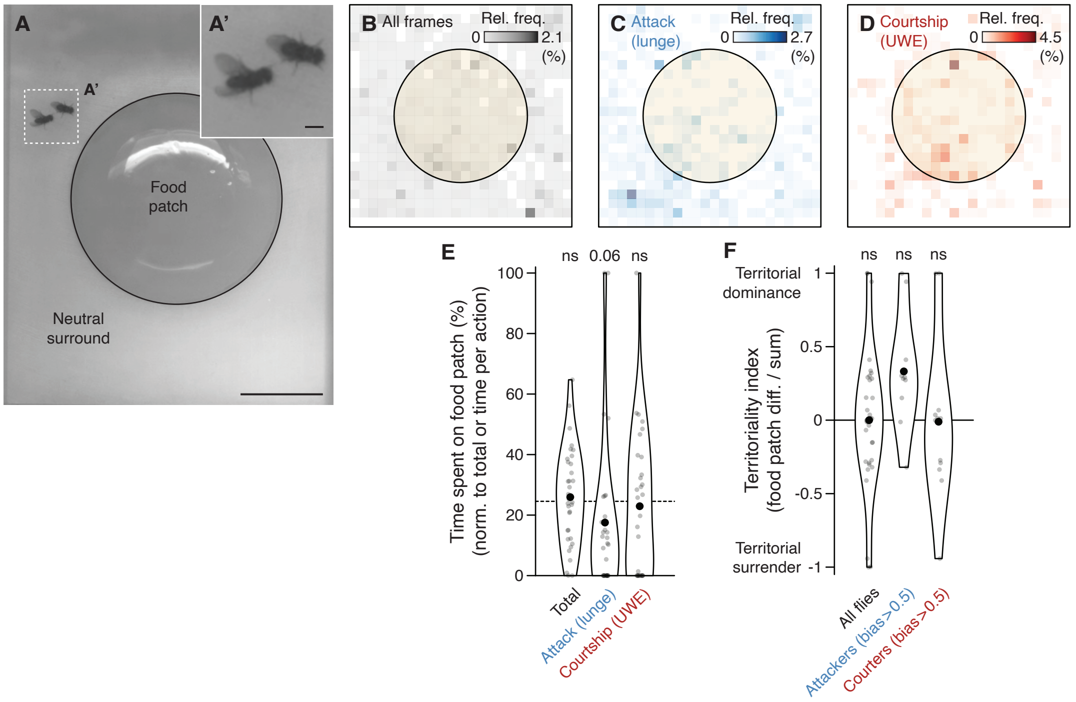
A
A'
A'
Food patch
Neutral surround
B All frames
Rel. freq.
0
2.1
(%)
C Attack (lunge)
Rel. freq.
0
2.7
(%)
D Courtship (UWE)
Rel. freq.
0
4.5
(%)
E
ns
0.06
ns
Time spent on food patch (%) (norm. to total or time per action)
100
80
60
40
20
0
Total
Attack (lunge)
Courtship (UWE)
F
ns
ns
ns
Territorial dominance
Territorial surrender
Territoriality index (food patch diff. / sum)
1
0.5
0
-0.5
-1
All flies
Attackers (bias>0.5)
Courters (bias>0.5)

Figure S9. Weak supporting evidence for food territoriality in *D. santomea* male pairs. Related to Figure 6.

(A) Video still of a pair of single-housed, wildtype *D. santomea* males in a large behavior chamber (40 mm wide x 50 mm long x 60 mm tall) containing an apple juice-based “food patch” (25 mm diameter) and neutral surround used to test territoriality. (A’) Close-up of intermale courtship from boxed region. Scale bars, 10 mm (A), 1 mm (A’).

(B-D) Heatmaps representing cumulative spatial patterns of all fly positions (B), attack (lunge) positions (C), and courtship (UWE) positions (D) for 17 *D. santomea* male-male pairs recorded during 10 min dyadic interactions. Normalization in (B) to all frames, in (C) to attack frames, and in (D) to courtship frames. Note little sign of increased density on or around the food patch in any case, unlike previously observed for *D. melanogaster* aggression in similar assays[S14].

(E) Quantification of (B-D) showing the fraction of time spent on the food patch in total, during attack, and during courtship for each individual *D. santomea* male. Normalizations as in heatmaps above. None are significantly different from a null median of 25% (random chance, calculated as surface area of the food patch relative to the full arena) by one-sample Mann-Whitney *U* tests. Attack frames show near-significant deviation from chance, but in the opposite direction from that expected for territoriality (i.e., enrichment off the food patch).

(F) “Territoriality index” for *D. santomea* males calculated as the difference in time spent on the food patch between flies normalized by the sum. First distribution represents all pairs, second filtered for pairs where one fly showed attack dominance, and third filtered for pairs where one fly showed courtship dominance. None are significantly different from a null median of zero (no territorial advantage) by one-sample Mann-Whitney *U* tests.

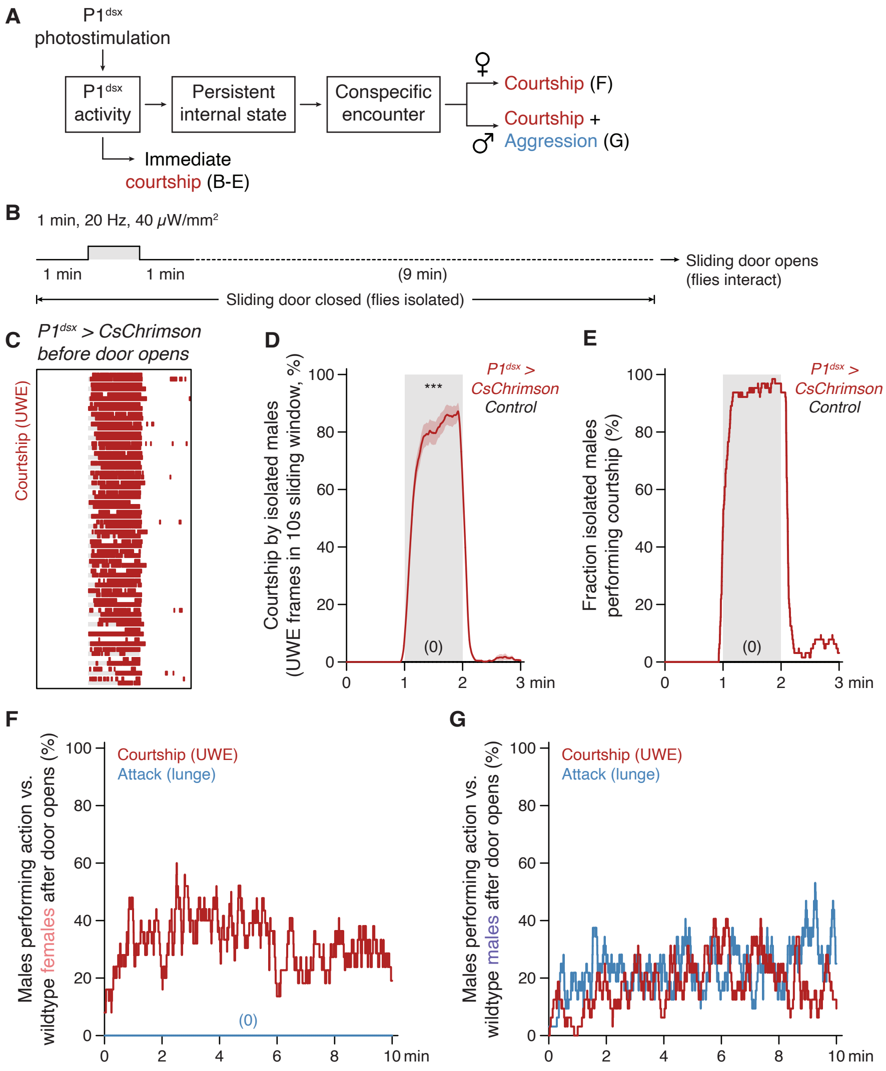
A
P1dsx photostimulation
P1dsx activity
Persistent internal state
Conspecific encounter
Courtship (F)
Courtship +
Aggression (G)
Immediate courtship (B-E)
B
1 min, 20 Hz, 40 μW/mm2
1 min
1 min
(9 min)
Sliding door opens (flies interact)
Sliding door closed (flies isolated)
C
P1dsx > CsChrimson before door opens
Courtship (UWE)
D
Courtship by isolated males (UWE frames in 10s sliding window, %)
P1dsx > CsChrimson
Control
***
(0)
3 min
E
Fraction isolated males performing courtship (%)
P1dsx > CsChrimson
Control
(0)
3 min
F
Males performing action vs. wildtype females after door opens (%)
Courtship (UWE)
Attack (lunge)
(0)
10 min
G
Males performing action vs. wildtype males after door opens (%)
Courtship (UWE)
Attack (lunge)
10 min

Figure S10. Additional behavioral characterizations for the *D. santomea* P1^{dsx} sliding door assay. Related to Figure 6

(A) Summary of immediate and delayed behavioral outcomes in *D. santomea* males following brief P1^{dsx} photostimulation (PS) in the delayed interaction ("sliding door") assay, with evidence below. P1^{dsx} PS evokes immediate, time-locked courtship during the early isolation phase and in parallel generates a persistent internal state of social arousal. Subsequent encounters with conspecific females or males during the interaction phase elicit pure courtship or mixed courtship and aggression, respectively. Panels containing relevant data for each behavioral outcome are indicated. Figure modified from ref. [S29].

(B) Optogenetic photoactivation scheme for isolated *D. santomea* males carrying $P1^{dsx}$ split-Gal4 ($71G01^{DBD};dsx^{AD}$) and Gal4-dependent CsChrimson during the early isolation phase of the trial. After a 1 min baseline, two group-housed $P1^{dsx}$ tester males on either side of a removable divider are exposed to a 1 min PS block, followed by a 10 min delay before doors are opened and flies allowed to interact. Delay phase shown as split into 1 min and 9 min subphases to reflect cessation of detailed courtship annotations 1 min after PS offset.

(C) Manually scored courtship rasters (UWE, red) for isolated $P1^{dsx}$ tester males during the first 3 min of the trial.

(D,E) Expressivity (D) and penetrance (E) of courtship elicited by PS. Traces in (D) show the mean fraction of frames containing UWE in a 10 s sliding window with s.e.m. envelopes for $P1^{dsx}$ (red) and a genetic control (black, males missing dsx^{AD}). (E) shows the fraction of flies performing courtship. Note extremely high penetrance and expressivity of evoked courtship time-locked to PS in $P1^{dsx}$ testers.

(F,G) Fraction of $P1^{dsx}$ tester males showing courtship (red) and attack (blue) toward wildtype conspecific females (F) or males (G) during the 10 min interaction phase after doors open. Exclusive courtship is observed toward females and a mix of courtship and attack toward males.

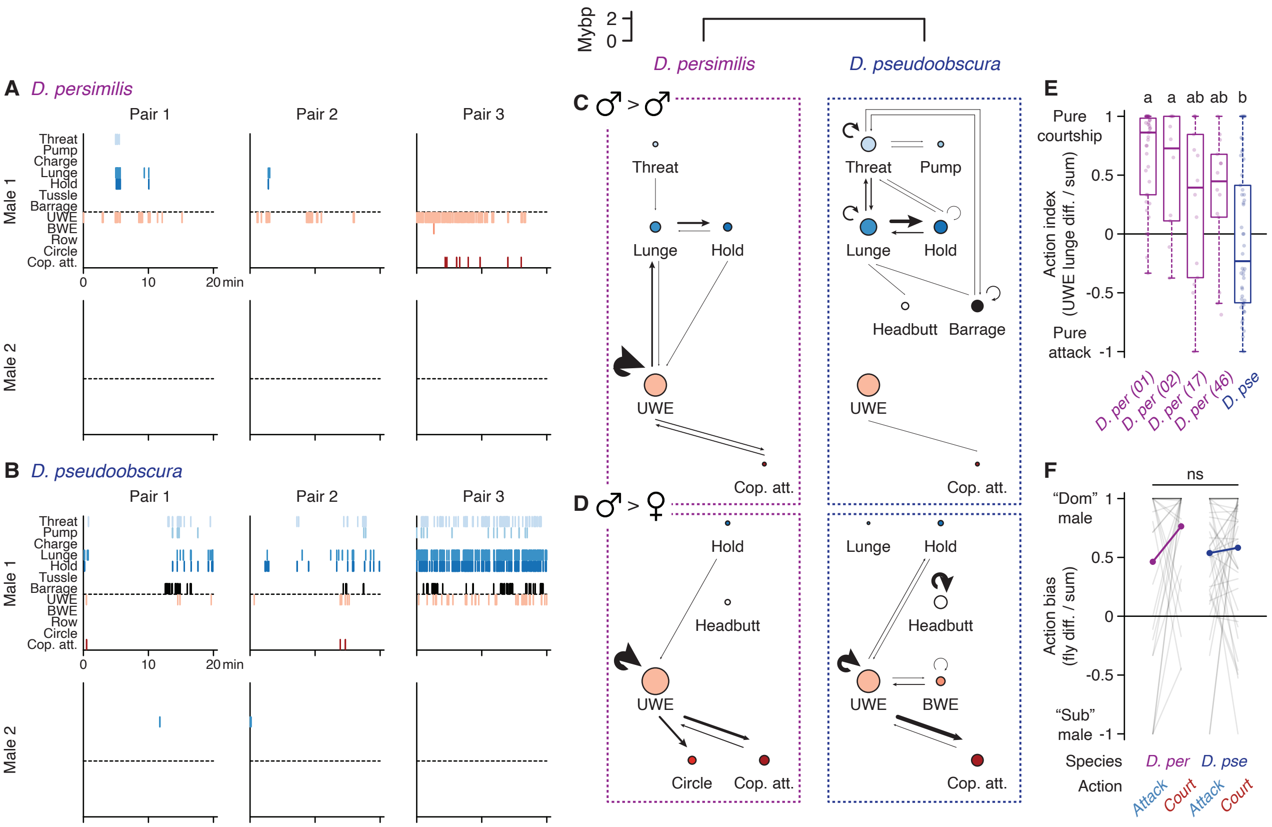

Mybp
2
0
D. persimilis
D. pseudoobscura
A D. persimilis
Pair 1
Pair 2
Pair 3
Threat
Pump
Charge
Lunge
Hold
Tussle
Barrage
UWE
BWE
Row
Circle
Cop. att.
Male 1
Male 2
0
10
20 min
B D. pseudoobscura
C ♂ > ♂
Threat
Pump
Lunge
Hold
Headbutt
Barrage
UWE
Cop. att.
D ♂ > ♀
Hold
Headbutt
UWE
BWE
Circle
Cop. att.
Lunge
E
a
a
ab
ab
b
Pure courtship
Pure attack
Action index (UWE lunge diff. / sum)
1
0.5
0
-0.5
-1
D. per (01)
D. per (02)
D. per (17)
D. per (46)
D. pse
F
ns
"Dom" male
"Sub" male
Action bias (fly diff. / sum)
Species
Action
D. per
D. pse
Attack
Court

Figure S11. Courtship and aggression by *D. persimilis* and *D. pseudoobscura* males. Related to Figure 7.

(A,B) Behavior rasters for three 20 min conspecific male-male pairs each of wildtype *D. persimilis* (A) and *D. pseudoobscura* (B). Annotations of seven aggressive interactions by manual scoring (blues and black) are shown above the horizontal dashed lines and five courtship actions (reds) beneath. Actions split by identity of the male to which they are attributed with the one exhibiting the greater total number of actions assigned as "Male 1." *D. persimilis* males court males frequently, including making copulation attempts (e.g., Pair 3). *D. pseudoobscura* also show some male-directed courtship but attack more intensely and often.

(C,D) Ethograms representing social behaviors by *D. persimilis* and *D. pseudoobscura* males in conspecific male-male (C) and male-female (D) pairings. Ethograms represent expert annotation from three male-male and three male-female pairs per species, with male tester flies prepared identically by single-housing and interactions taking place under identical conditions. Male-directed ethograms are reproduced from Figure S1A for visual comparison to female-directed counterparts. Note similarity between interactions with males and females in *D. persimilis*.

(E) Action index observed by flies in male-male *D. persimilis* and *D. pseudoobscura* pairs, with comparison to additional *D. persimilis* wildtype strains. The index is calculated as the difference in the number of observed courtship (UWE) and attack (lunge) bouts within each fly pair normalized by the sum (pairs showing more courtship than attack deflect upward). *D. per (00)* is the *D. persimilis* strain used elsewhere in the figure and as the representative *D. persimilis* wildtype strain otherwise throughout. *D. persimilis* strains consistently show high relative courtship expression, with some inter-strain variability. Significance by Dunn's tests.

(F) Spontaneous social action biases observed between flies in male-male *D. persimilis* and *D. pseudoobscura* pairs for attack and courtship. Indices derived from the same fly pair are connected as lines and distribution means shown as paired purple (*D. persimilis*) or blue dots (*D. pseudoobscura*). Both species show strong inter-fly behavioral biases.

Compound	Retention time start	Retention time end	Diagnostic ions
C18	11.433	11.5	254
methyl palmitate	12.192	12.258	74,87,270
C21	13.317	13.458	296
C22:1	13.992	14.058	308
cVA	14.067	14.142	250,310
C22	14.15	14.217	310
C23:1n9	14.783	14.858	322
C23:1n7	14.858	14.92	322
C23:1n6	14.92	14.96	322
C23:1n5	14.96	15	322
C23	15.008	15.075	324
C24:1n7	15.75	15.833	336
C24:1n5	15.842	15.908	336
C24	15.917	16.017	338
2meC24	16.533	16.633	309,337
C25:1n9	16.675	16.767	350
C25:1n7	16.783	16.85	350
C25	16.925	17.017	352
2meC25	17.6	17.675	323,351
3meC25?	17.733	17.792	56,337
C26:1_1	17.867	17.908	364
C26:1_2	17.917	17.967	364
C26	17.983	18.108	366
2meC26	18.633	18.817	337,365,380
C27:1n7	18.967	19.05	378
C27	19.133	19.217	380
2meC27	19.858	19.942	351,379,394
3meC27	19.983	20.075	337,365
C28	20.275	20.375	394
2meC28	20.917	21.167	365,393
C29:1n7	21.325	21.408	406
C29	21.467	21.575	408
7meC29	21.958	22.075	112,309,337
3meC29	22.367	22.467	365,393
C30	22.667	22.783	422
ester_2	23.383	23.45	406
2meC30	23.458	23.525	393,421
C31	23.867	23.992	436
C32:?	24.425	24.575	(not determined)
ester_4	24.617	24.717	448
ester_5	26.575	26.758	386,404
CH503	27.017	27.242	386,404,446,464

Table S1. *D. santomea* cuticular hydrocarbons assigned by GC-MS. Related to Figure 2 and Figure S4.

Supplemental References

S1. Duistermars, B.J., Pfeiffer, B.D., Hoopfer, E.D., and Anderson, D.J. (2018). A Brain Module for Scalable Control of Complex, Multi-motor Threat Displays. Neuron *100*, 1474-1490.e4. https://doi.org/10.1016/j.neuron.2018.10.027.

S2. Chen, S., Lee, A.Y., Bowens, N.M., Huber, R., and Kravitz, E.A. (2002). Fighting fruit flies: A model system for the study of aggression. PNAS *99*, 5664–5668. https://doi.org/10.1073/pnas.082102599.

S3. Nilsen, S.P., Chan, Y.-B., Huber, R., and Kravitz, E.A. (2004). Gender-selective patterns of aggressive behavior in Drosophila melanogaster. PNAS *101*, 12342–12347. https://doi.org/10.1073/pnas.0404693101.

S4. Cobb, M., Connolly, K., and Burnet, B. (1985). Courtship Behaviour in the Melanogaster Species Sub-Group of Drosophila. Behaviour *95*, 203–230. https://doi.org/10.1163/156853985x00136.

S5. Gregg, T., and Hahn, M. (2017). Drosophila 25 species phylogeny. figshare. https://doi.org/10.6084/m9.figshare.5450602.v1.

S6. Turissini, D.A., and Matute, D.R. (2017). Fine scale mapping of genomic introgressions within the Drosophila yakuba clade. PLOS Genet *13*, e1006971. https://doi.org/10.1371/journal.pgen.1006971.

S7. Miller, D.E., Staber, C., Zeitlinger, J., and Hawley, R.S. (2018). Highly Contiguous Genome Assemblies of 15 Drosophila Species Generated Using Nanopore Sequencing. G3 *8*, 3131–3141. https://doi.org/10.1534/g3.118.200160.

S8. Partridge, L., Hoffmann, A., and Jones, J.S. (1987). Male size and mating success in Drosophila melanogaster and D. pseudoobscura under field conditions. Anim Behav *35*, 468–476. https://doi.org/10.1016/s0003-3472(87)80272-5.

S9. Eyjolfsdottir, E., Branson, S., Burgos-Artizzu, X.P., Hoopfer, E.D., Schor, J., Anderson, D.J., and Perona, P. (2014). Detecting Social Actions of Fruit Flies. ECCV, 772–787. https://doi.org/10.1007/978-3-319-10605-2_50.

S10. Hoopfer, E.D., Jung, Y., Inagaki, H.K., Rubin, G.M., and Anderson, D.J. (2015). P1 interneurons promote a persistent internal state that enhances inter-male aggression in Drosophila. eLife *4*, e11346. https://doi.org/10.7554/elife.11346.

S11. Kabra, M., Robie, A.A., Rivera-Alba, M., Branson, S., and Branson, K. (2013). JAABA: interactive machine learning for automatic annotation of animal behavior. Nat Methods *10*, 64–67. https://doi.org/10.1038/nmeth.2281.

S12. Cariou, M. -L., Silvain, J. -F., Daubin, V., Lage, J. -L. D., and Lachaise, D. (2001). Divergence between Drosophila santomea and allopatric or sympatric populations of D. yakuba using paralogous amylase genes and migration scenarios along the Cameroon volcanic line. Mol Ecol *10*, 649–660. https://doi.org/10.1046/j.1365-294x.2001.01225.x.

S13. Auer, T.O., Shahandeh, M.P., and Benton, R. (2021). Drosophila sechellia: A Genetic Model for Behavioral Evolution and Neuroecology. Annu Rev Genet *55*, 1–28. https://doi.org/10.1146/annurev-genet-071719-020719.

S14. Lim, R.S., Eyjólfsdóttir, E., Shin, E., Perona, P., and Anderson, D.J. (2014). How Food Controls Aggression in Drosophila. PLOS One 9, e105626. https://doi.org/10.1371/journal.pone.0105626 .

S15. Ding, Y., Lillvis, J.L., Cande, J., Berman, G.J., Arthur, B.J., Long, X., Xu, M., Dickson, B.J., and Stern, D.L. (2019). Neural Evolution of Context-Dependent Fly Song. Curr Biol *29*, 1089-1099.e7. https://doi.org/10.1016/j.cub.2019.02.019.

S16. Sawtelle, S., Narayan, L., Ding, Y., Kim, E., Behrman, E.L., Lillvis, J.L., Kawase, T., and Stern, D.L. (2024). Song Torrent: A modular, open-source 96-chamber audio and video recording apparatus with optogenetic activation and inactivation capabilities for Drosophila. bioRxiv, 2024.01.09.574712. https://doi.org/10.1101/2024.01.09.574712.

S17. Tompkins, L., Gross, A.C., Hall, J.C., Gailey, D.A., and Siegel, R.W. (1982). The role of female movement in the sexual behavior of Drosophila melanogaster. Behav Genet *12*, 295–307. https://doi.org/10.1007/bf01067849.

S18. Pan, Y., Meissner, G.W., and Baker, B.S. (2012). Joint control of Drosophila male courtship behavior by motion cues and activation of male-specific P1 neurons. PNAS *109*, 10065–10070. https://doi.org/10.1073/pnas.1207107109.

S19. Agrawal, S., Safarik, S., and Dickinson, M. (2014). The relative roles of vision and chemosensation in mate recognition of Drosophila melanogaster. J Exp Biol *217*, 2796–2805. https://doi.org/10.1242/jeb.105817.

S20. Ribeiro, I.M.A., Drews, M., Bahl, A., Machacek, C., Borst, A., and Dickson, B.J. (2018). Visual Projection Neurons Mediating Directed Courtship in Drosophila. Cell *174*, 607-621.e18. https://doi.org/10.1016/j.cell.2018.06.020.

S21. Stern, D.L., Kim, E., and Behrman, E.L. (2024). The Janelia Atalanta plasmids provide a simple and efficient CRISPR/Cas9-mediated homology directed repair platform for Drosophila. bioRxiv, 2023.06.17.545412. https://doi.org/10.1101/2023.06.17.545412.

S22. Coleman, R.T., Morantte, I., Koreman, G.T., Cheng, M.L., Ding, Y., and Ruta, V. (2024). A modular circuit coordinates the diversification of courtship strategies. Nature *635*, 142–150. https://doi.org/10.1038/s41586-024-08028-1.

S23. Kurtovic, A., Widmer, A., and Dickson, B.J. (2007). A single class of olfactory neurons mediates behavioural responses to a Drosophila sex pheromone. Nature *446*, 542–546. https://doi.org/10.1038/nature05672.

S24. Depetris-Chauvin, A., Galagovsky, D., Keesey, I.W., Hansson, B.S., Sachse, S., and Knaden, M. (2023). Evolution at multiple processing levels underlies odor-guided behavior in the genus Drosophila. Curr Biol *33*, 4771-4785.e7. https://doi.org/10.1016/j.cub.2023.09.039.

S25. Jallon, J., and David, J.R. (1987). Variations in Cuticular Hydrocarbons Among the Eight Species of the Drosophila melanogaster Subgroup. Evolution *41*, 294–302. https://doi.org/10.1111/j.1558-5646.1987.tb05798.x.

S26. Lacaille, F., Hiroi, M., Twele, R., Inoshita, T., Umemoto, D., Manière, G., Marion-Poll, F., Ozaki, M., Francke, W., Cobb, M., et al. (2007). An Inhibitory Sex Pheromone Tastes Bitter for Drosophila Males. PLOS One *2*, e661. https://doi.org/10.1371/journal.pone.0000661.

S27. Billeter, J.-C., Atallah, J., Krupp, J.J., Millar, J.G., and Levine, J.D. (2009). Specialized cells tag sexual and species identity in Drosophila melanogaster. Nature 461, 987–991. https://doi.org/10.1038/nature08495.

S28. Wang, L., Han, X., Mehren, J., Hiroi, M., Billeter, J.-C., Miyamoto, T., Amrein, H., Levine, J.D., and Anderson, D.J. (2011). Hierarchical chemosensory regulation of male-male social interactions in Drosophila. Nat Neurosci *14*, 757–762. https://doi.org/10.1038/nn.2800.

S29. Jung, Y., Kennedy, A., Chiu, H., Mohammad, F., Claridge-Chang, A., and Anderson, D.J. (2020). Neurons that Function within an Integrator to Promote a Persistent Behavioral State in Drosophila. Neuron *105*, 322-333.e5. https://doi.org/10.1016/j.neuron.2019.10.028.
